# Supplementary material for: Population-based genome-wide association study of plasma complex lipid species
Source: Nat Commun. 2026 May 2;17:3984. doi: 10.1038/s41467-026-72542-1 (PMC13135507; doi:10.1038/s41467-026-72542-1)
Supplement: Supplementary file 1 — Supplementary Information [file 41467_2026_72542_MOESM1_ESM.pdf]

Supplementary Table 1: Overview of the total lipid concentrations per lipid class,

Lipid category	Lipid class	n= 6,096
Neutral lipids	Triacylglycerols (TAGs)	1,099 (680,6)
	Diacylglycerols (DAGs)	28,2 (23,6)
	Monoacylglycerols (MAGs)	17,1 (62,3)
	Cholesteryl esters (CEs)	2778,8 (593,4)
Phospholipids	Phosphatidylcholines (PCs)	2,016,3 (387,5)
	Phosphatidylethanolamines* (PEs)	147,6 (39,3)
	Phosphatidylinositols (PIs)	23,4 (10,7)
	Lysophosphatidylcholines (LPCs)	178,9 (41,7)
	Lysophosphatidylethanolamines (LPEs)	6,2 (1,9)
Sphingolipids	Ceramides (CERs)	5,6 (1,4)
	Dihydrosylceramides (DCERs)	1,5 (0,5)
	Hexosylceramides (HCERs)	4,3 (1,1)
	Lactosylceramides (LCERs)	3,3 (0,7)
	Sphingomyelins (SMs)	485,6 (88,2)

All concentrations are displayed as mean (standard deviation) in nmol/ml

**Includes PE esters, PE plasmalogens and PE ethers*

Abbreviations used: Ceramides (CER), Dihydrosylceramides (DCER), Hexosylceramides (HCER), Lactosylceramides (LCER), Sphingomyelin (SM), Phosphatidylcholine (PC), Phosphatidylethanolamine (PE), Phosphatidylinositol (PI), Lysophosphatidylcholine (LPC), Lysophosphatidylethanolamine (LPE)

Supplementary Table 2: Overview of the FinnGen and EPIC-Potsdam sample characteristics

**Characteristic**	**FinnGen (n= 7,266)**	**EPIC-Potsdam participants (n= 1,188)**
**Age (years)**	55,8 (5,8)	50,3 (9,0)
**Sex (women)**	4,642 (63,9)	716 (60)
**LDL-C (mg/dL)**	129,93 (35,96)	122,0 (35,2)
**HDL-C (mg/dL)**	64,20 (18,56)	55,8 (14,7)
**Triglycerides (mg/dL)**	112,48 (74,40)	135,0 (100,0)
**Cholesterol (mg/dL)**	215,78 (39,83)	204,0 (42,4)
**Use of lipid-lowering medication (yes)**	805 (11,1)	68 (5,7)

Data displayed represent the number of participants (percentages) for categorical variables and the mean (standard deviation) for continuous variables, Abbreviations: HDL-C = high-density lipoprotein cholesterol, LDL-C = low-density lipoprotein cholesterol,

Supplementary Table 3: An overview of the lipid nomenclature and number of categories, classes, species and fatty acid composite measures

**Lipid categories (n= 3)**	**Lipid classes (n= 14)**	**Species (n= 970)***		**Fatty acid composite (n= 267)***	
		Number (%)	*Example*	*Number (%)*	*Example*
Neutral lipids **(n= 628)**	Triacylglycerols (TAGs)	518 (53,1%)	TAG58:6(FA22:4))	21 (7,8%)	TAG(FA12:0)
	Diacylglycerols (DAGs)	58 (5,9%)	DAG(14:0/14:0)	19 (7,0%)	DAG(FA18:3)
	Monoacylglycerols (MAGs)	26 (2,7%)	MAG(20:1)	Same as species	
	Cholesteryl esters (CEs)	26 (2,7%)	CE(24:1)	Same as species	
Phospholipids **(n= 286)**	Phosphatidylcholines (PCs)	104 (10,7%)	PC(16:0/20:3)	23 (8,5%)	PC(FA20:1)
	Phosphatidylethanolamines (PEs)**	122 (127,5%)	PE(18:1/18:2)	18 (7,0%)	PE(FA17:0)
	Phosphatidylinositols (PIs)	22 (2,7%)	PI(16:0/20:3)	12 (4,8%)	PI(FA22:6)
	Lysophosphatidylcholines (LPCs)	18 (1,8%)	LPC(20:0)	Same as species	
	Lysophosphatidylethanolamines (LPEs)	15 (1,6%)	LPE(15:0)	Same as species	
Sphingolipids **(n= 61)**	Sphingomyelins (SMs)	12 (1,2%)	SM(18:1)	Same as species	
	Ceramides (CERs)	12 (1,2%)	CER(22:1)	Same as species	
	Dihydrosylceramides (DCERs)	13 (1,3%)	DCER(24:0)	Same as species	
	Hexosylceramides (HCERs)	12 (1,2%)	HCER(26:0)	Same as species	
	Lactosylceramides (LCERs)	12 (1,2%)	LCER(14:0)	Same as species	

**Lipid species are defined by complete information on the total number of carbons and double bonds, whereas fatty acid composite measures sum up the concentration of all lipids within a class of a specific length and degree of saturation, In case of one-tailed lipids, the fatty acid composite measure is thus equal to the species, In addition, we also have information on the total fatty acid composition (n= 28), which sums up the concentration of a specific fatty acid tail across all lipid classes,*

***Containing PE esters, PE ethers, and PE plasmalogens*

Supplementary Table 4: GWAS replication results per lipid class in the EPIC-Potsdam cohort,

Lipid class		Model 1			Model 2		
	No, of lipid species in Rhineland Study	Metabolome-wide significant lipid species in Rhineland Study	Replicated number of species per lipid class in EPIC-Potsdam	Replication (%) per lipid class	Metabolome-wide significant lipid species in Rhineland Study	Replicated number of species per lipid class in EPIC-Potsdam	Replication (%) per lipid class
TAG	518	405	387	95,5	230	228	99,1
PE	58	24	22	91,6	25	25	100
PEP	44	12	10	83,3	11	11	100
PC	104	41	38	92,7	44	44	100
DAG	58	44	42	95,4	34	33	97
LPC	18	8	5	62,5	11	10	90,9
HCER	11	9	8	88,8	10	8	80
LCER	12	8	4	50,0	10	6	60
PI	22	15	13	86,6	15	15	100
CER	12	7	4	57,1	8	5	62,5
MAG	26	9	5	55,5	9	7	77,7
CE	26	21	11	52,4	17	15	88,2
SM	12	12	5	41,6	10	7	70,0
PEO	20	6	3	50	6	6	100
LPE	15	9	4	44,4	9	7	77,7
DCER	13	8	2	25,0	7	5	71,5

Supplementary Figure 1: Heatmap of correlations among concentrations of 970 lipid species, Lipid species are grouped based on hierarchical clustering

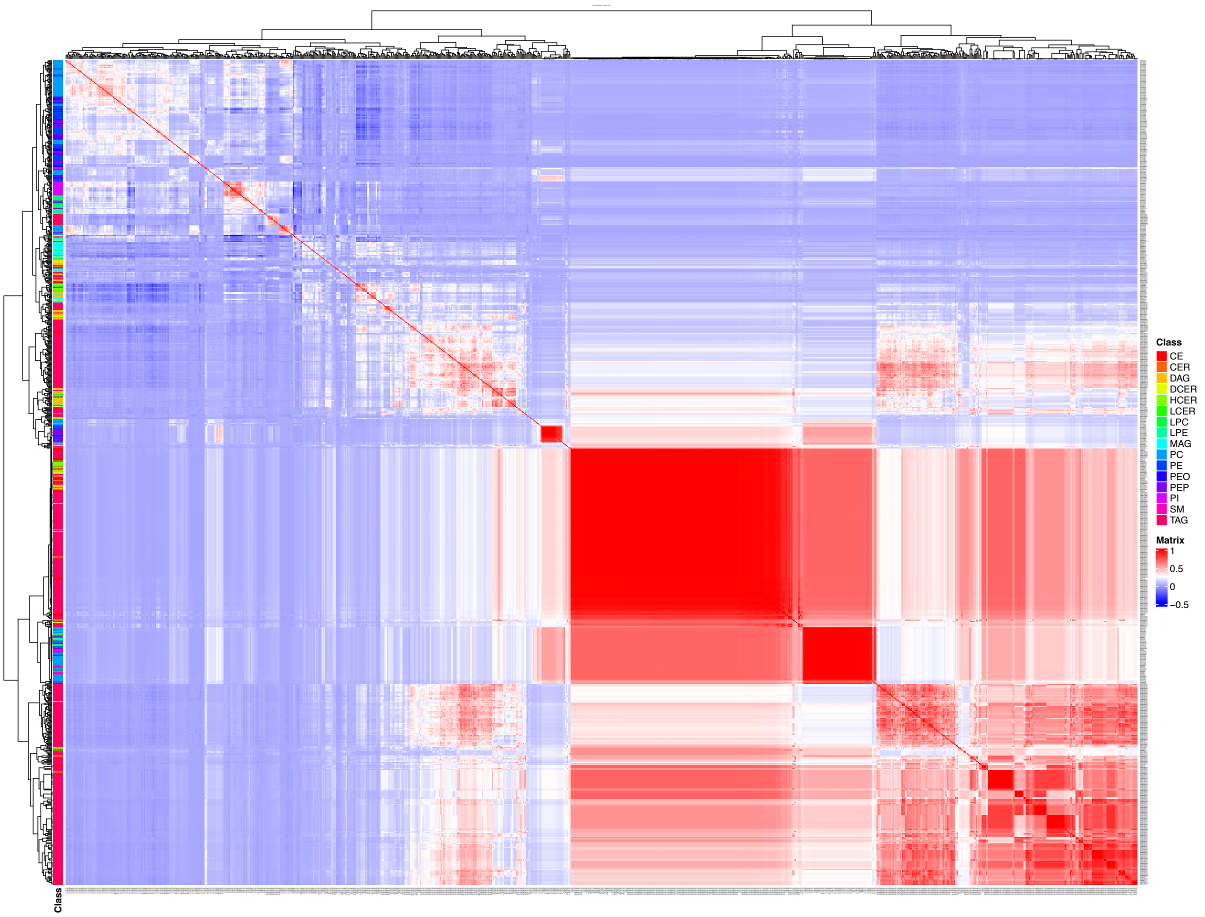

Supplementary Figure 2: Locus zoom plots for genomic loci identified in model 1 (a) and model 2 (b)

(a)

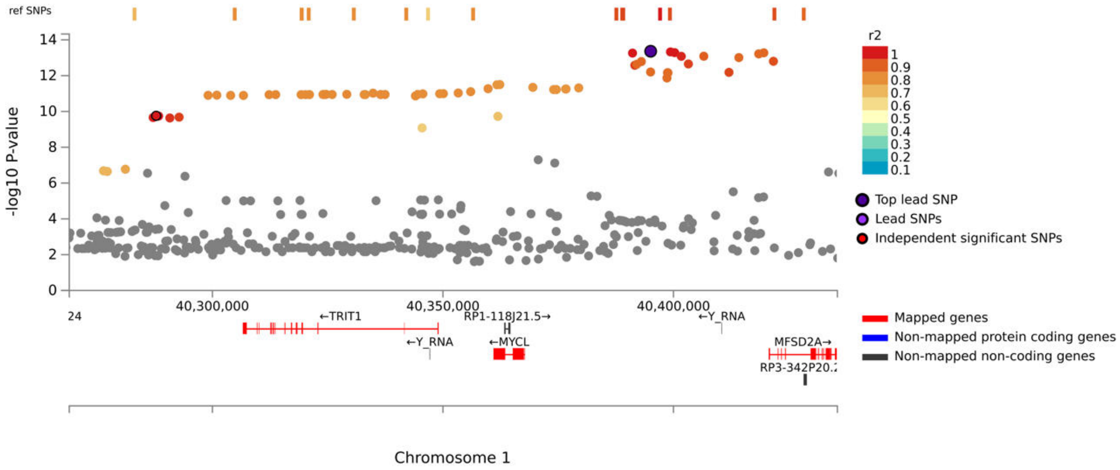

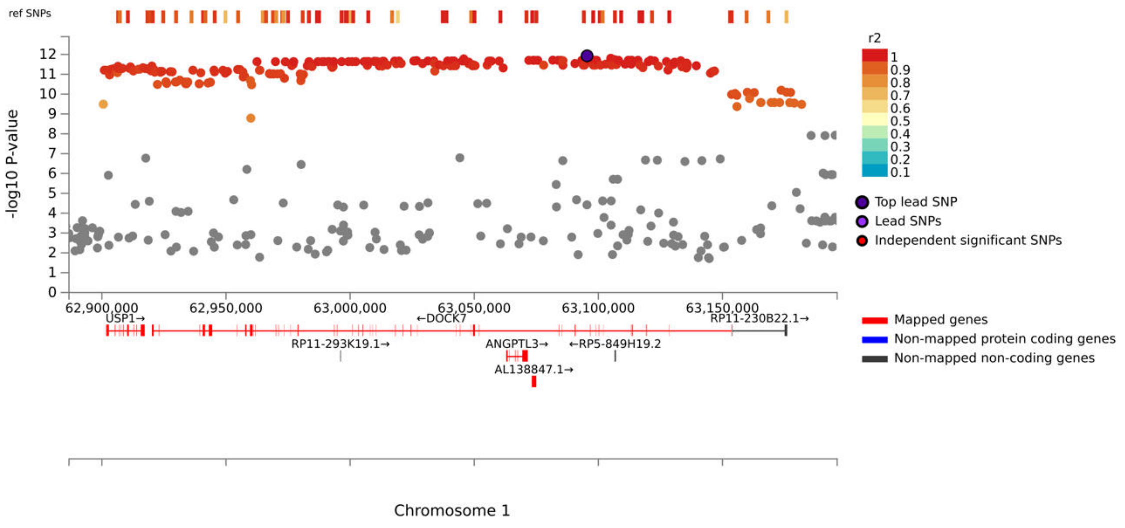
ref SNPs
-log10 P-value
62,900,000
62,950,000
63,000,000
63,050,000
63,100,000
63,150,000
USP1→
←DOCK7
RP11-230B22.1→
RP11-293K19.1→
ANGPTL3→
←RP5-849H19.2
AL138847.1→
Chromosome 1
r2
Top lead SNP
Lead SNPs
Independent significant SNPs
Mapped genes
Non-mapped protein coding genes
Non-mapped non-coding genes

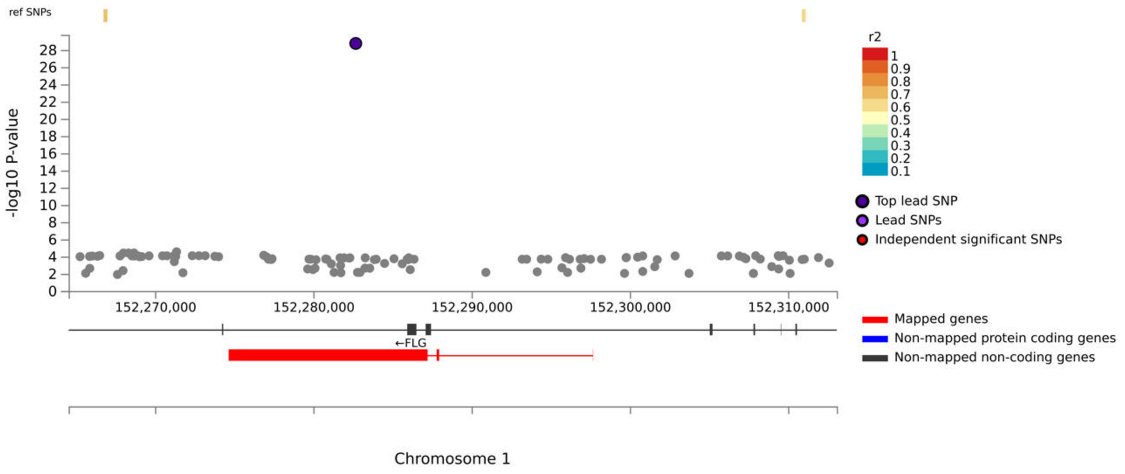
ref SNPs
-log10 P-value
152,270,000
152,280,000
152,290,000
152,300,000
152,310,000
←FLG
Chromosome 1
r2
Top lead SNP
Lead SNPs
Independent significant SNPs
Mapped genes
Non-mapped protein coding genes
Non-mapped non-coding genes

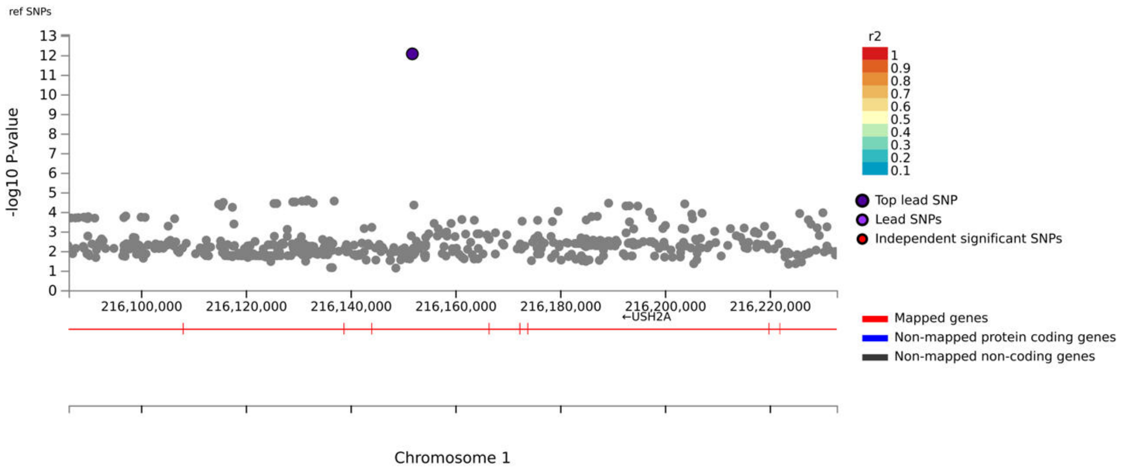
ref SNPs
-log10 P-value
216,100,000
216,120,000
216,140,000
216,160,000
216,180,000
216,200,000
216,220,000
←USH2A
Chromosome 1
r2
Top lead SNP
Lead SNPs
Independent significant SNPs
Mapped genes
Non-mapped protein coding genes
Non-mapped non-coding genes

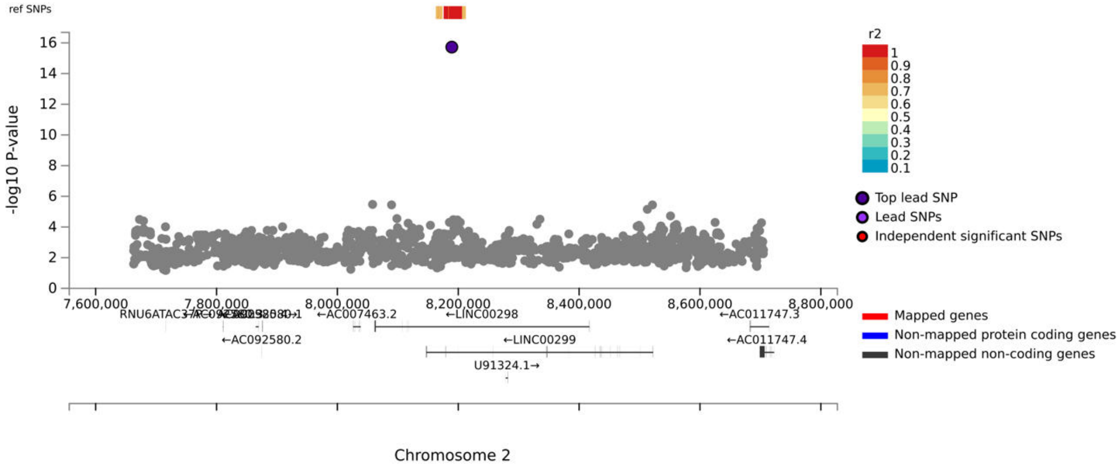
ref SNPs
-log10 P-value
r2
Top lead SNP
Lead SNPs
Independent significant SNPs
←AC007463.2
←LINC00298
←AC092580.2
←LINC00299
U91324.1→
←AC011747.3
←AC011747.4
Mapped genes
Non-mapped protein coding genes
Non-mapped non-coding genes
Chromosome 2

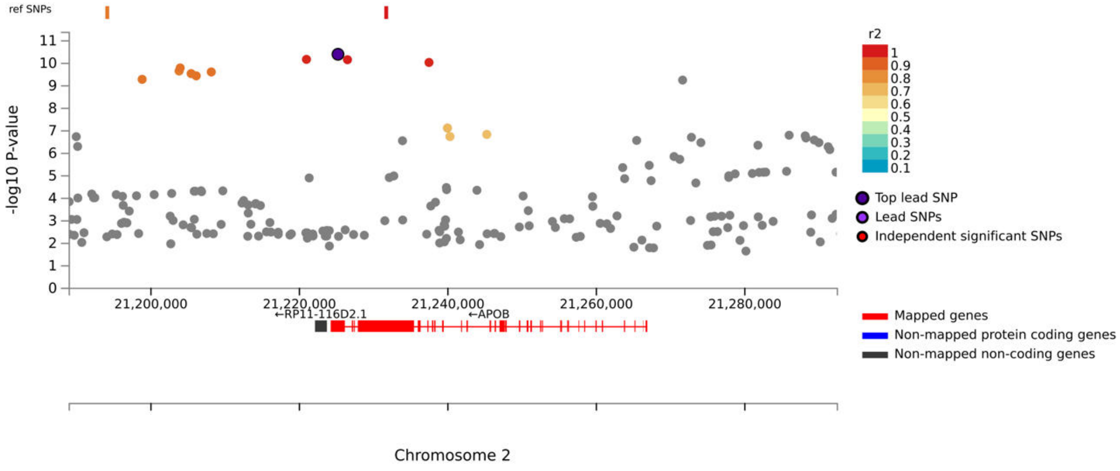
ref SNPs
-log10 P-value
r2
Top lead SNP
Lead SNPs
Independent significant SNPs
←RP11-116D2.1
←APOB
Mapped genes
Non-mapped protein coding genes
Non-mapped non-coding genes
Chromosome 2

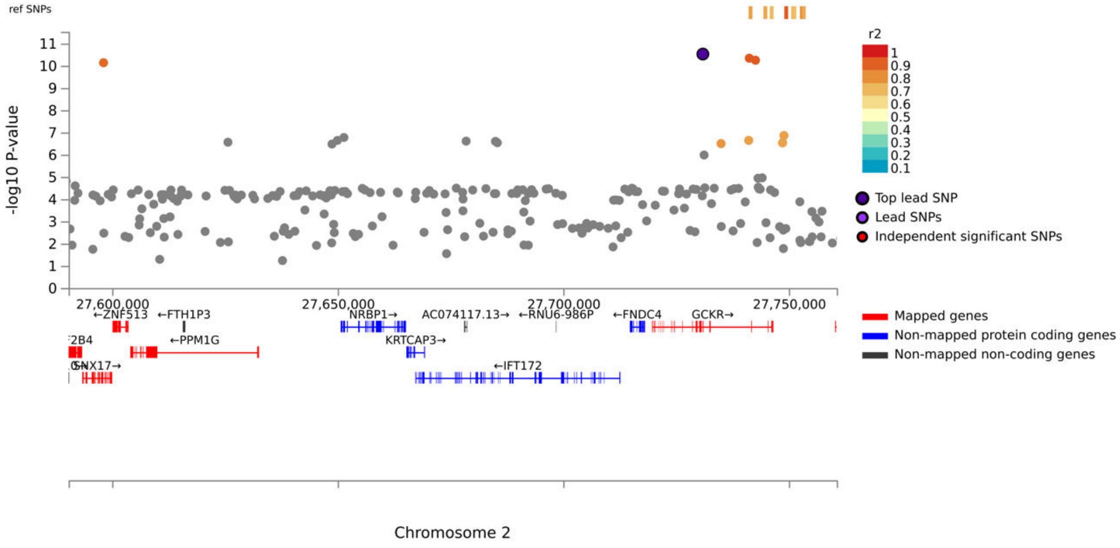
ref SNPs
-log10 P-value
r2
Top lead SNP
Lead SNPs
Independent significant SNPs
←ZNF513
←FTH1P3
NRBP1→
AC074117.13→
←RNU6-986P
←FNDC4
GCKR→
←PPM1G
KRTCAP3→
←IFT172
Mapped genes
Non-mapped protein coding genes
Non-mapped non-coding genes
Chromosome 2

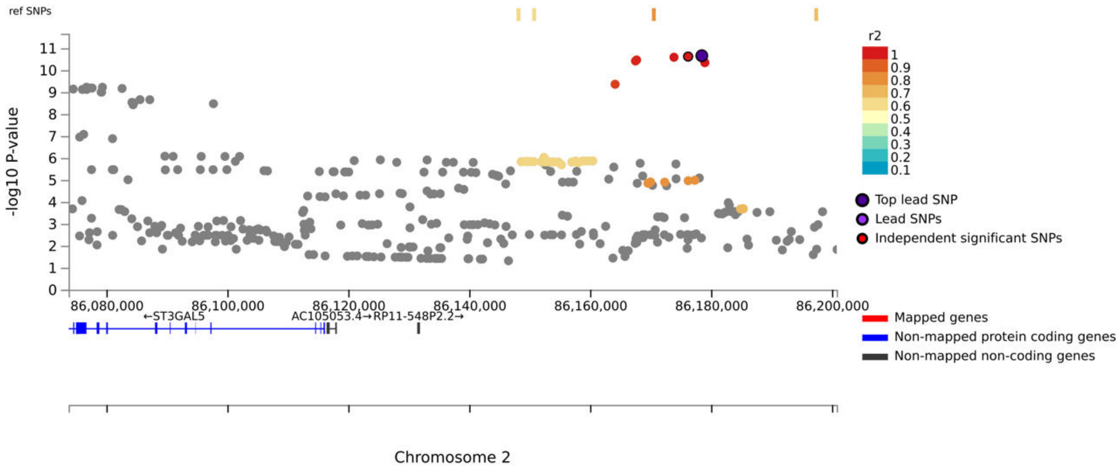
ref SNPs
-log10 P-value
r2
1
0.9
0.8
0.7
0.6
0.5
0.4
0.3
0.2
0.1
Top lead SNP
Lead SNPs
Independent significant SNPs
86,080,000
86,100,000
86,120,000
86,140,000
86,160,000
86,180,000
86,200,000
←ST3GAL5
AC105053.4→RP11-548P2.2→
Mapped genes
Non-mapped protein coding genes
Non-mapped non-coding genes
Chromosome 2

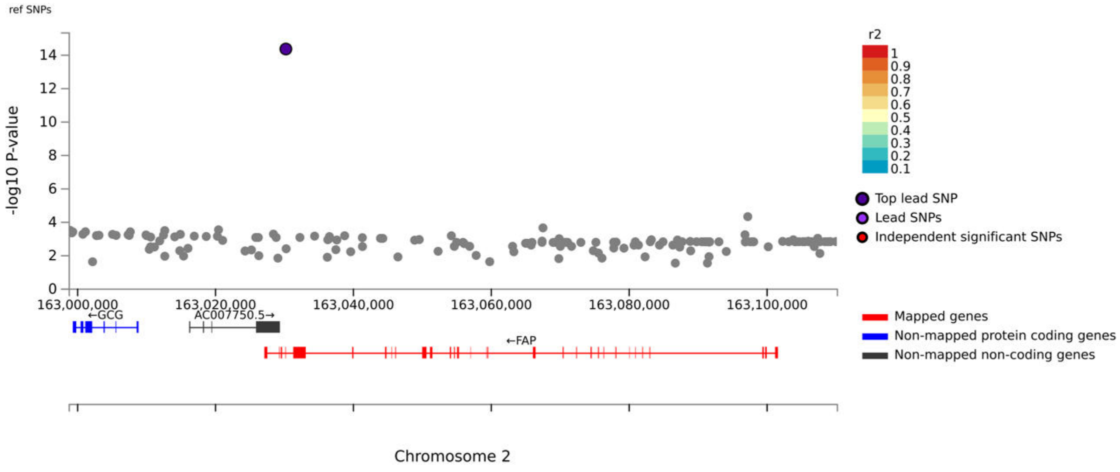
ref SNPs
-log10 P-value
r2
1
0.9
0.8
0.7
0.6
0.5
0.4
0.3
0.2
0.1
Top lead SNP
Lead SNPs
Independent significant SNPs
163,000,000
163,020,000
163,040,000
163,060,000
163,080,000
163,100,000
←GCG
AC007750.5→
←FAP
Mapped genes
Non-mapped protein coding genes
Non-mapped non-coding genes
Chromosome 2

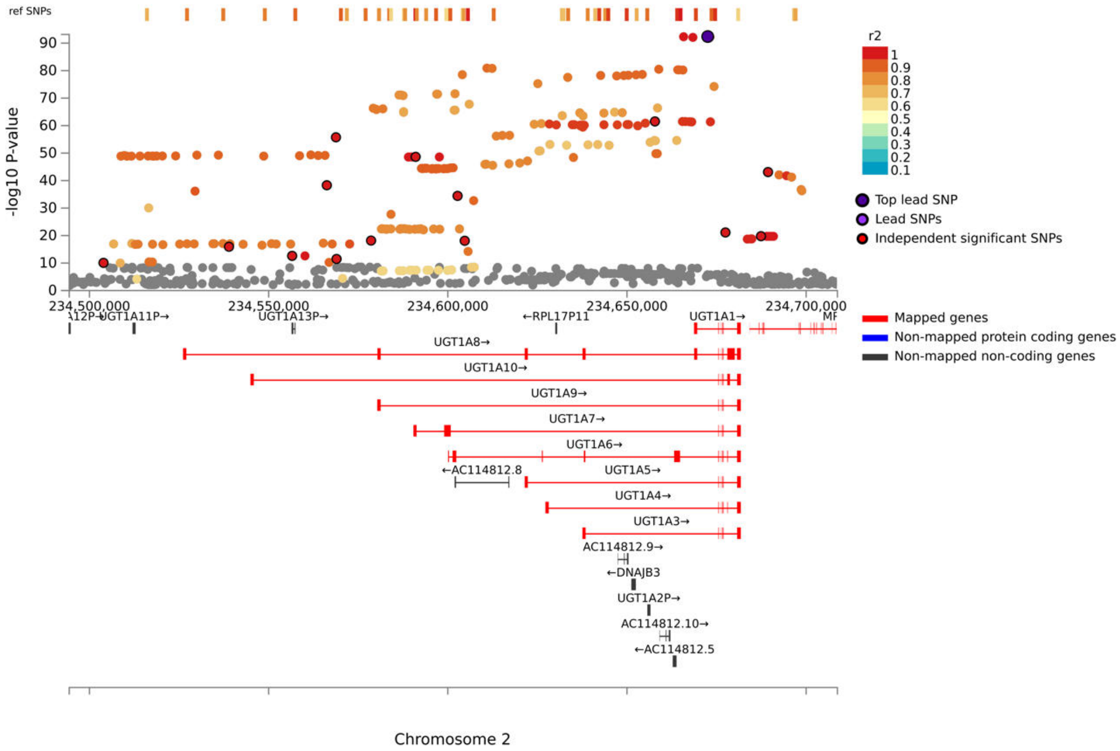
ref SNPs
-log10 P-value
r2
1
0.9
0.8
0.7
0.6
0.5
0.4
0.3
0.2
0.1
Top lead SNP
Lead SNPs
Independent significant SNPs
234,500,000
234,550,000
234,600,000
234,650,000
234,700,000
UGT1A11P→
UGT1A13P→
←RPL17P11
UGT1A1→
UGT1A8→
UGT1A10→
UGT1A9→
UGT1A7→
UGT1A6→
←AC114812.8
UGT1A5→
UGT1A4→
UGT1A3→
AC114812.9→
←DNAJB3
UGT1A2P→
AC114812.10→
←AC114812.5
Mapped genes
Non-mapped protein coding genes
Non-mapped non-coding genes
Chromosome 2

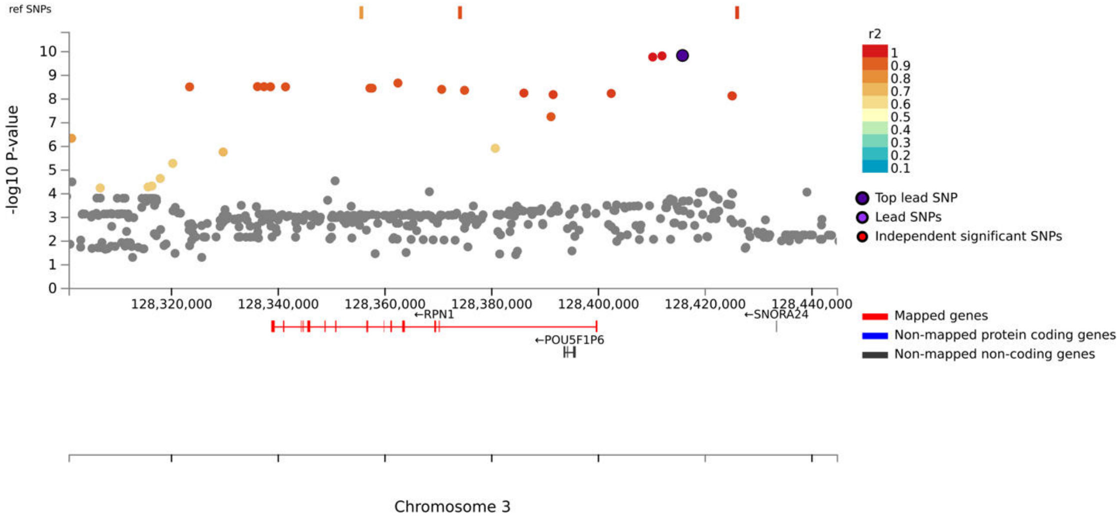
ref SNPs
-log10 P-value
r2
1
0.9
0.8
0.7
0.6
0.5
0.4
0.3
0.2
0.1
Top lead SNP
Lead SNPs
Independent significant SNPs
128,320,000
128,340,000
128,360,000
128,380,000
128,400,000
128,420,000
128,440,000
←RPN1
←SNORA24
←POU5F1P6
Mapped genes
Non-mapped protein coding genes
Non-mapped non-coding genes
Chromosome 3

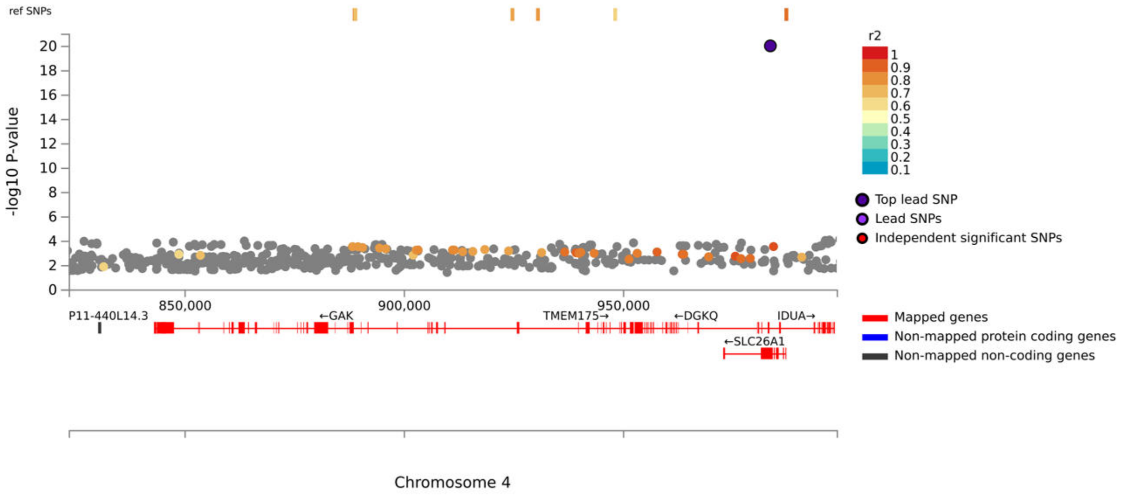
ref SNPs
-log10 P-value
r2
1
0.9
0.8
0.7
0.6
0.5
0.4
0.3
0.2
0.1
Top lead SNP
Lead SNPs
Independent significant SNPs
850,000
900,000
950,000
P11-440L14.3
←GAK
TMEM175→
←DGKQ
IDUA→
←SLC26A1
Mapped genes
Non-mapped protein coding genes
Non-mapped non-coding genes
Chromosome 4

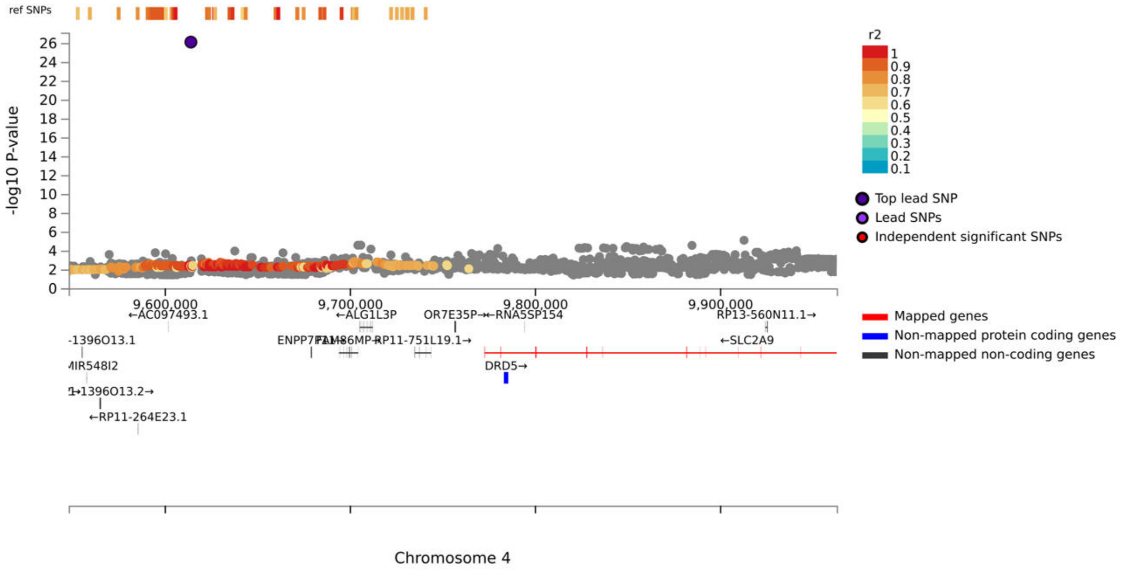
ref SNPs
-log10 P-value
r2
1
0.9
0.8
0.7
0.6
0.5
0.4
0.3
0.2
0.1
Top lead SNP
Lead SNPs
Independent significant SNPs
9,600,000
9,700,000
9,800,000
9,900,000
←AC097493.1
←ALG1L3P
OR7E35P→
←RNA5SP154
RP13-560N11.1→
←SLC2A9
DRD5→
←RP11-264E23.1
Mapped genes
Non-mapped protein coding genes
Non-mapped non-coding genes
Chromosome 4

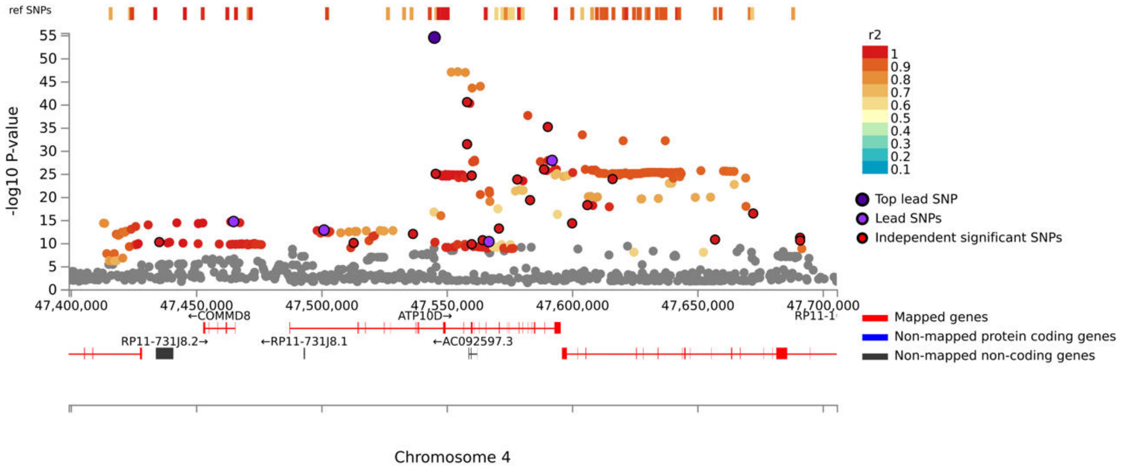
ref SNPs
-log10 P-value
r2
1
0.9
0.8
0.7
0.6
0.5
0.4
0.3
0.2
0.1
Top lead SNP
Lead SNPs
Independent significant SNPs
47,400,000
47,450,000
47,500,000
47,550,000
47,600,000
47,650,000
47,700,000
←COMMD8
ATP10D→
RP11-731J8.2→
←RP11-731J8.1
←AC092597.3
Mapped genes
Non-mapped protein coding genes
Non-mapped non-coding genes
Chromosome 4

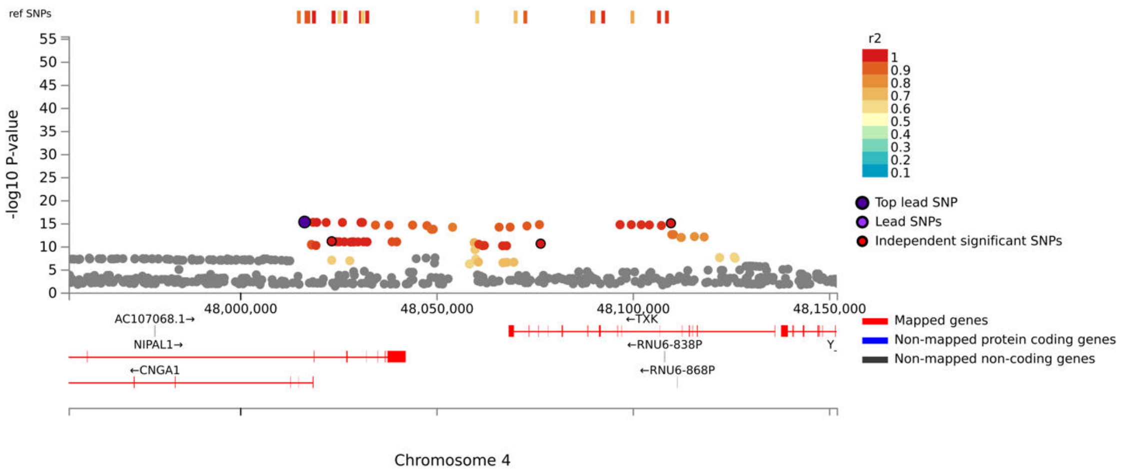
ref SNPs
-log10 P-value
r2
Top lead SNP
Lead SNPs
Independent significant SNPs
48,000,000
48,050,000
48,100,000
48,150,000
AC107068.1→
NIPAL1→
←CNGA1
←TXK
←RNU6-838P
←RNU6-868P
Mapped genes
Non-mapped protein coding genes
Non-mapped non-coding genes
Chromosome 4

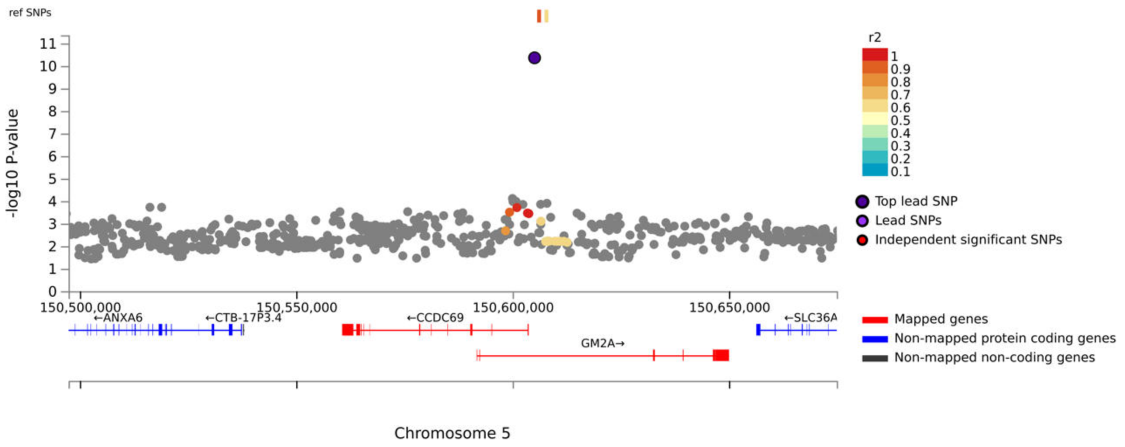
ref SNPs
-log10 P-value
r2
Top lead SNP
Lead SNPs
Independent significant SNPs
150,500,000
150,550,000
150,600,000
150,650,000
←ANXA6
←CTB-17P3.4
←CCDC69
GM2A→
←SLC36A
Mapped genes
Non-mapped protein coding genes
Non-mapped non-coding genes
Chromosome 5

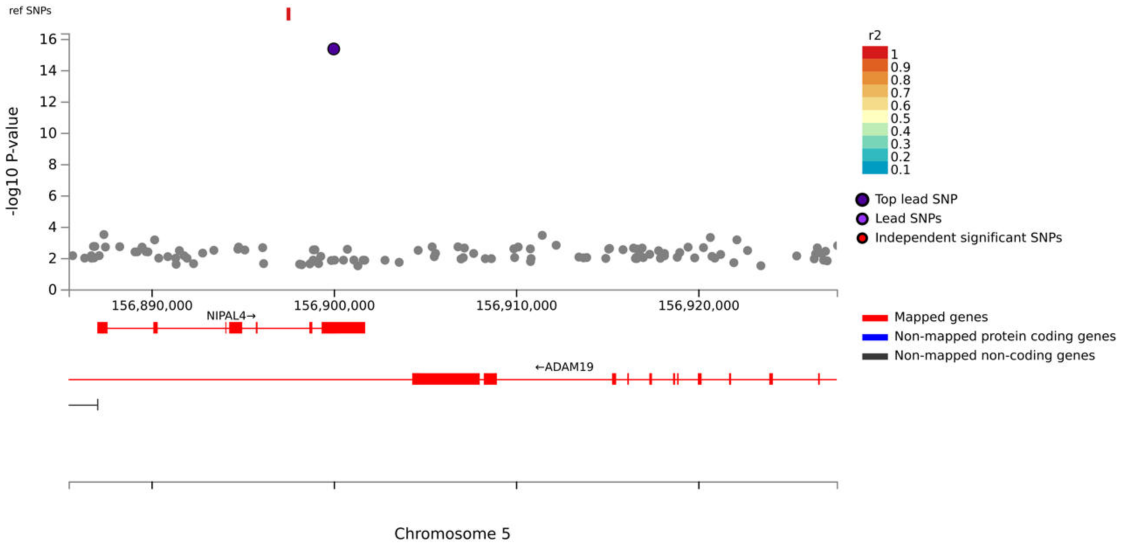
ref SNPs
-log10 P-value
r2
Top lead SNP
Lead SNPs
Independent significant SNPs
156,890,000
156,900,000
156,910,000
156,920,000
NIPAL4→
←ADAM19
Mapped genes
Non-mapped protein coding genes
Non-mapped non-coding genes
Chromosome 5

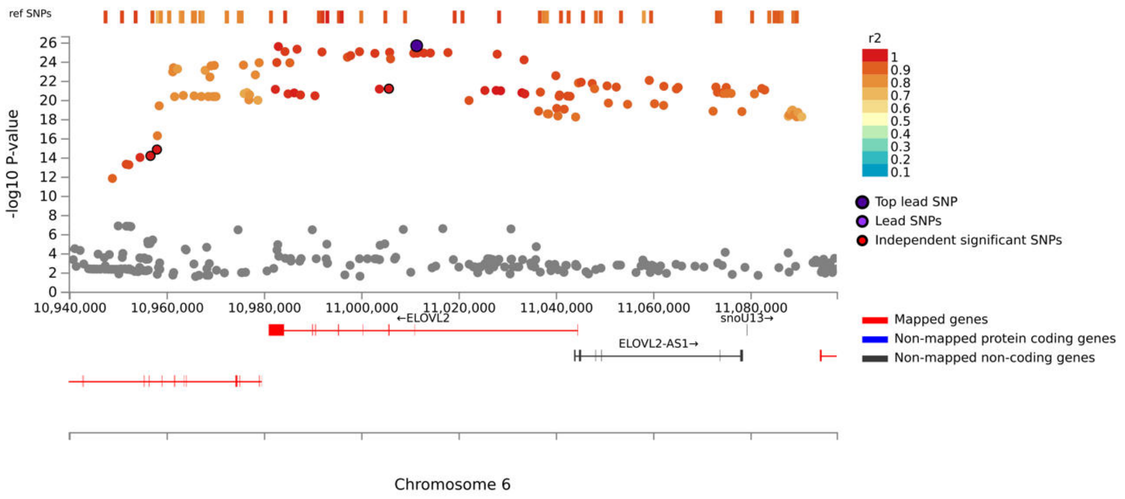
ref SNPs
-log10 P-value
r2
1
0.9
0.8
0.7
0.6
0.5
0.4
0.3
0.2
0.1
Top lead SNP
Lead SNPs
Independent significant SNPs
10,940,000
10,960,000
10,980,000
11,000,000
11,020,000
11,040,000
11,060,000
11,080,000
←ELOVL2
snoU13→
ELOVL2-AS1→
Mapped genes
Non-mapped protein coding genes
Non-mapped non-coding genes
Chromosome 6

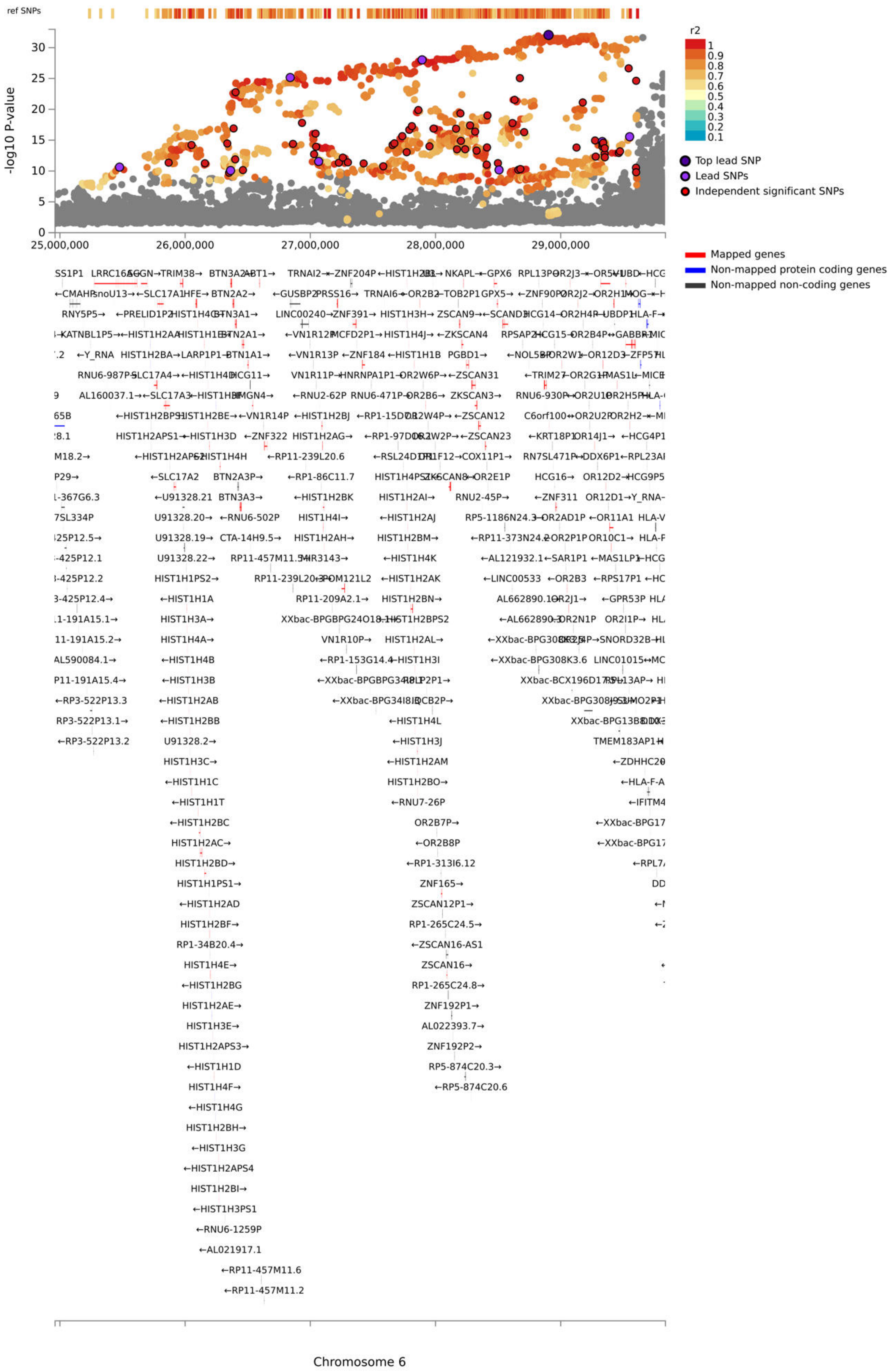

ref SNPs
-log10 P-value
0
5
10
15
20
25
30
25,000,000
26,000,000
27,000,000
28,000,000
29,000,000
r2
1
0.9
0.8
0.7
0.6
0.5
0.4
0.3
0.2
0.1
Top lead SNP
Lead SNPs
Independent significant SNPs
Mapped genes
Non-mapped protein coding genes
Non-mapped non-coding genes
Chromosome 6

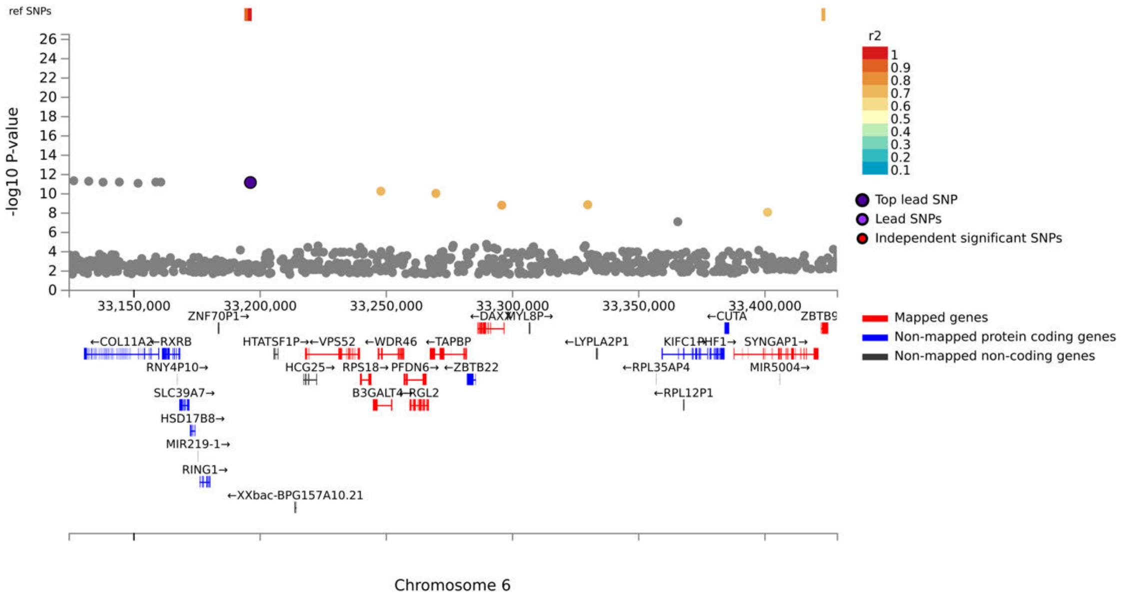
ref SNPs
-log10 P-value
26
24
22
20
18
16
14
12
10
8
6
4
2
0
33,150,000
33,200,000
33,250,000
33,300,000
33,350,000
33,400,000
ZNF70P1→
←DAXX
MYL8P→
←CUTA
←COL11A2
←RXRB
HTATSF1P→
←VPS52
←WDR46
←TAPBP
←LYPLA2P1
KIFC1
PHF1→
SYNGAP1→
RNY4P10→
HCG25→
RPS18→
PFDN6→
←ZBTB22
←RPL35AP4
MIR5004→
SLC39A7→
B3GALT4
←RGL2
←RPL12P1
HSD17B8→
MIR219-1→
RING1→
←XXbac-BPG157A10.21
Chromosome 6
r2
1
0.9
0.8
0.7
0.6
0.5
0.4
0.3
0.2
0.1
Top lead SNP
Lead SNPs
Independent significant SNPs
Mapped genes
Non-mapped protein coding genes
Non-mapped non-coding genes

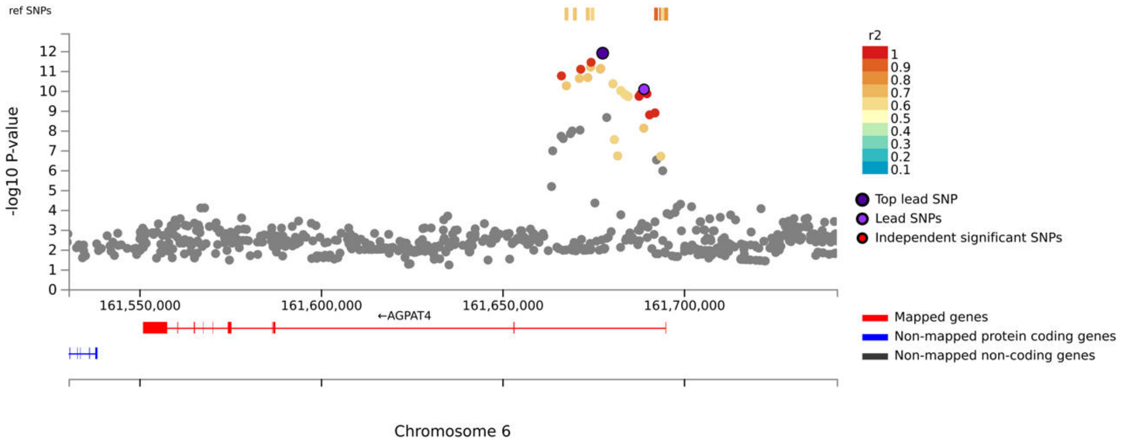
ref SNPs
-log10 P-value
12
11
10
9
8
7
6
5
4
3
2
1
0
161,550,000
161,600,000
161,650,000
161,700,000
←AGPAT4
Chromosome 6
r2
1
0.9
0.8
0.7
0.6
0.5
0.4
0.3
0.2
0.1
Top lead SNP
Lead SNPs
Independent significant SNPs
Mapped genes
Non-mapped protein coding genes
Non-mapped non-coding genes

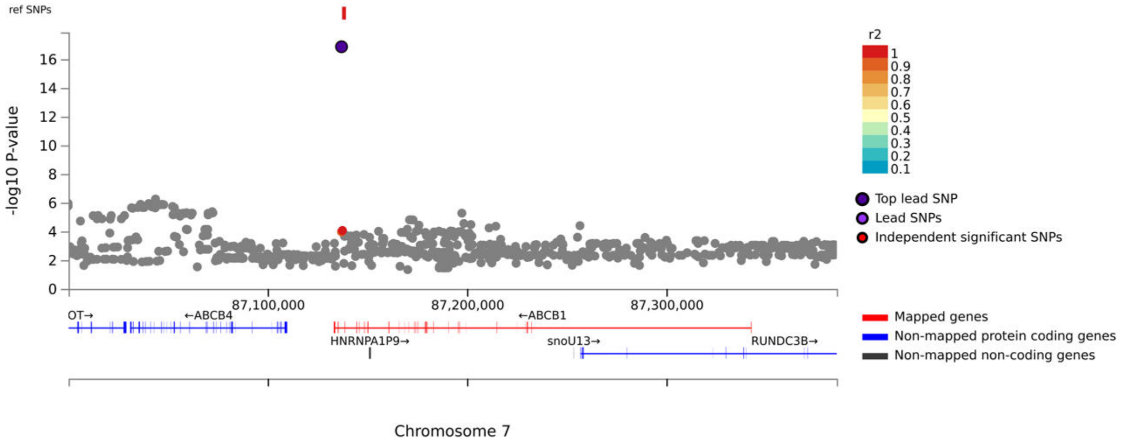
ref SNPs
-log10 P-value
16
14
12
10
8
6
4
2
0
87,100,000
87,200,000
87,300,000
OT→
←ABCB4
←ABCB1
HNRNPA1P9→
snoU13→
RUNDC3B→
Chromosome 7
r2
1
0.9
0.8
0.7
0.6
0.5
0.4
0.3
0.2
0.1
Top lead SNP
Lead SNPs
Independent significant SNPs
Mapped genes
Non-mapped protein coding genes
Non-mapped non-coding genes

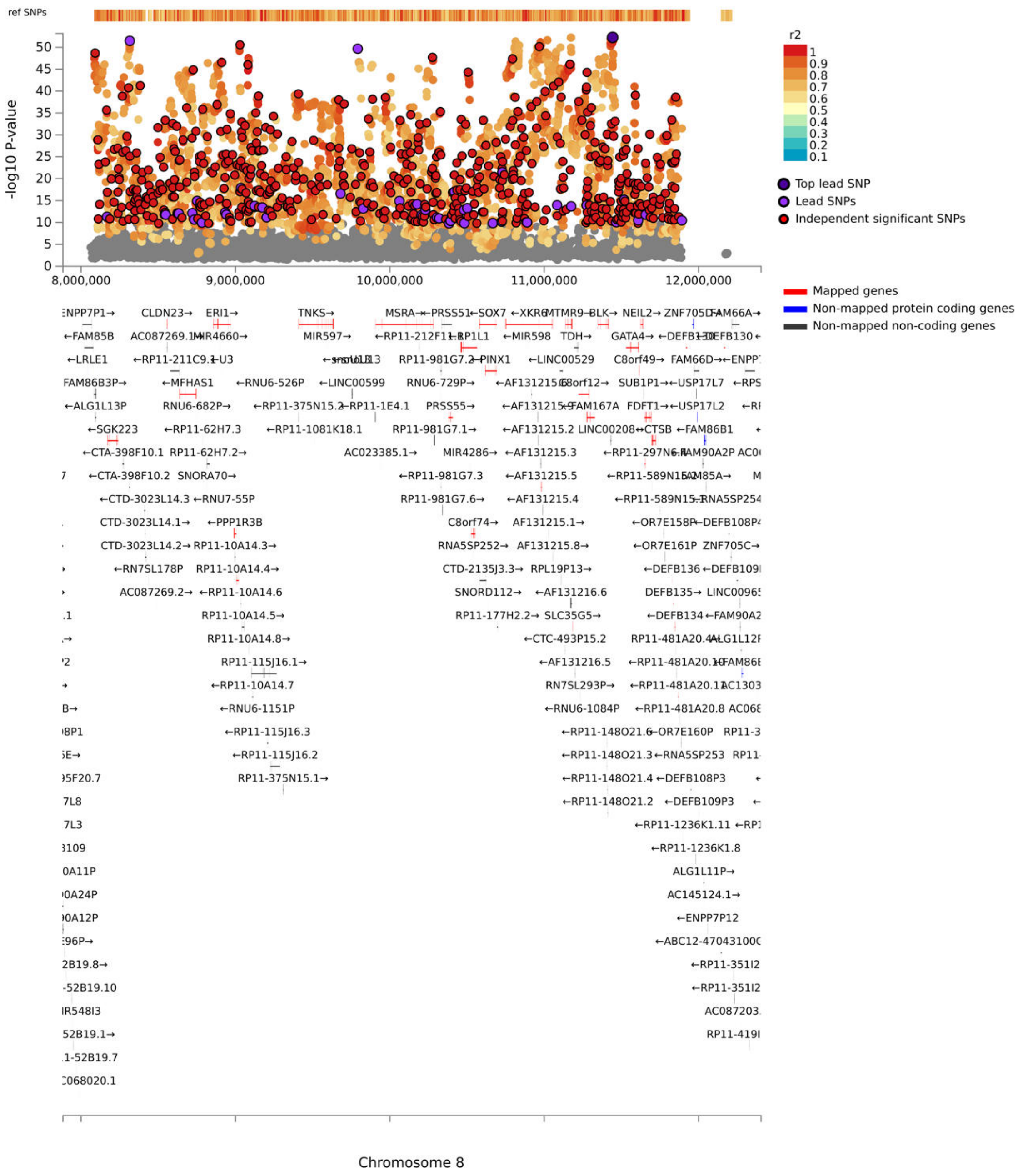
ref SNPs
-log10 P-value
0
5
10
15
20
25
30
35
40
45
50
8,000,000
9,000,000
10,000,000
11,000,000
12,000,000
r2
1
0.9
0.8
0.7
0.6
0.5
0.4
0.3
0.2
0.1
Top lead SNP
Lead SNPs
Independent significant SNPs
Mapped genes
Non-mapped protein coding genes
Non-mapped non-coding genes
Chromosome 8

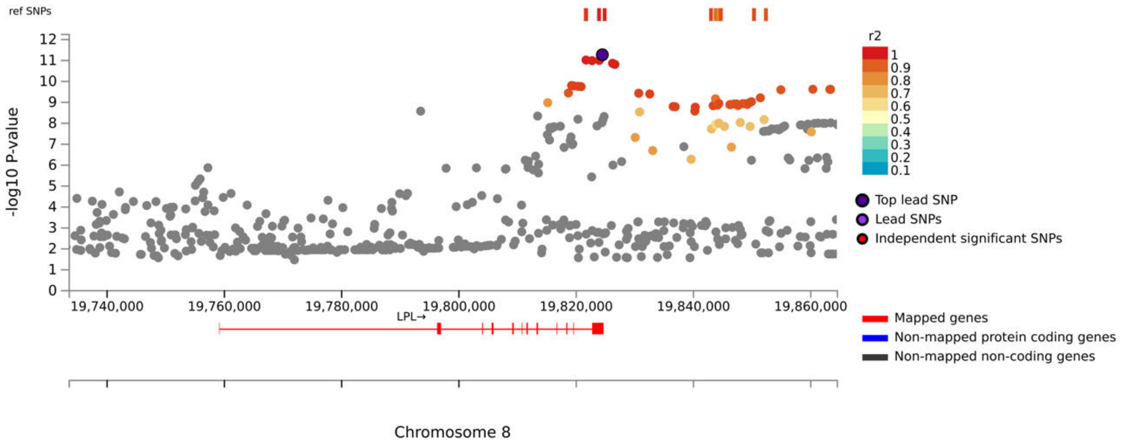
ref SNPs
-log10 P-value
r2
Top lead SNP
Lead SNPs
Independent significant SNPs
LPL→
Mapped genes
Non-mapped protein coding genes
Non-mapped non-coding genes
Chromosome 8

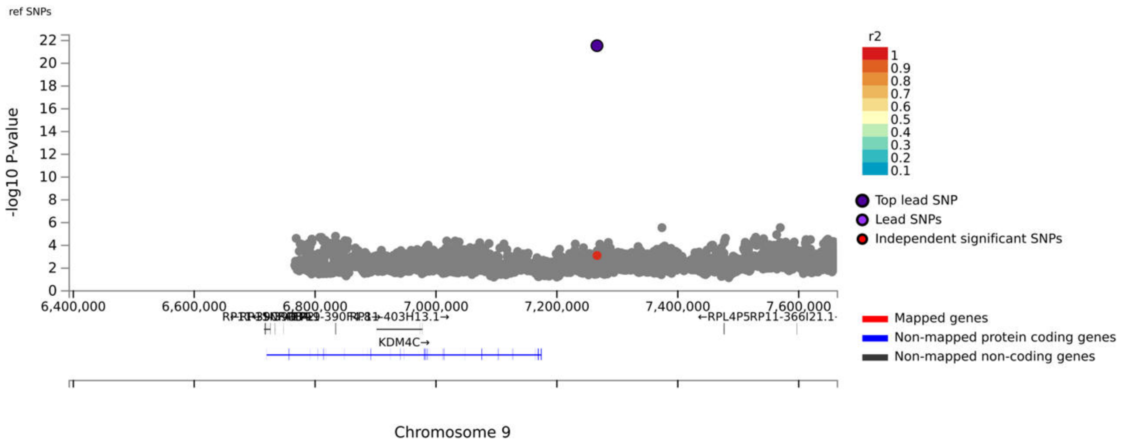
ref SNPs
-log10 P-value
r2
Top lead SNP
Lead SNPs
Independent significant SNPs
KDM4C→
←RPL4P5
Mapped genes
Non-mapped protein coding genes
Non-mapped non-coding genes
Chromosome 9

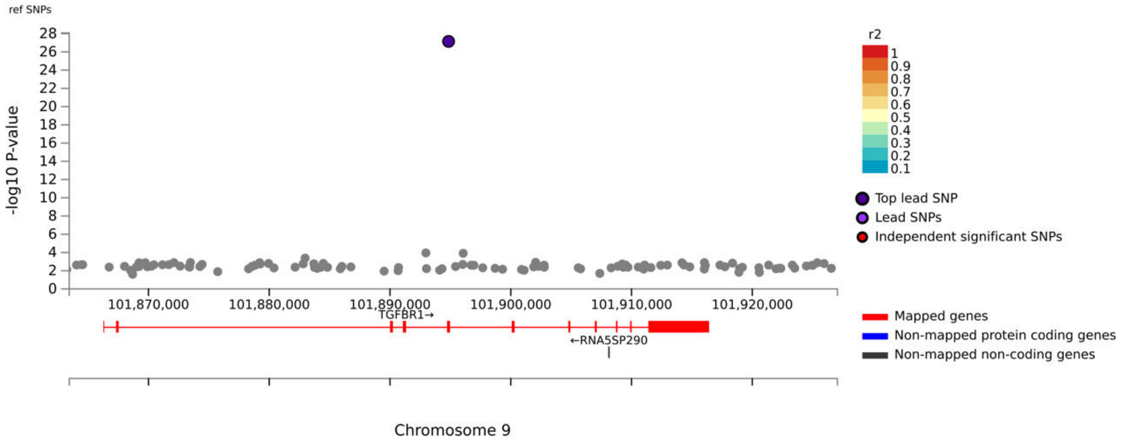
ref SNPs
-log10 P-value
r2
Top lead SNP
Lead SNPs
Independent significant SNPs
TGFBR1→
←RNA5SP290
Mapped genes
Non-mapped protein coding genes
Non-mapped non-coding genes
Chromosome 9

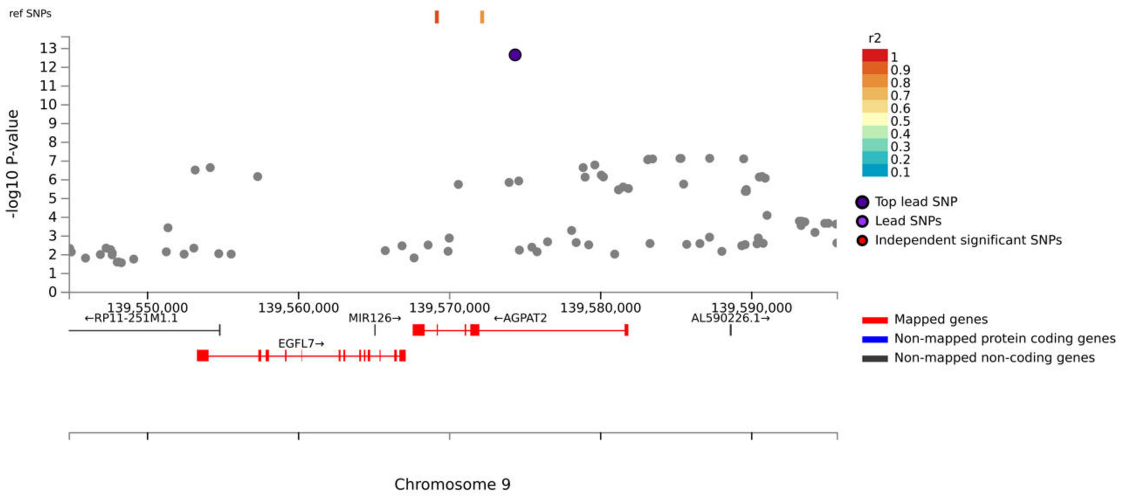
ref SNPs
-log10 P-value
r2
1
0.9
0.8
0.7
0.6
0.5
0.4
0.3
0.2
0.1
Top lead SNP
Lead SNPs
Independent significant SNPs
139,550,000
139,560,000
139,570,000
139,580,000
139,590,000
←RP11-251M1.1
MIR126→
←AGPAT2
AL590226.1→
EGFL7→
Mapped genes
Non-mapped protein coding genes
Non-mapped non-coding genes
Chromosome 9

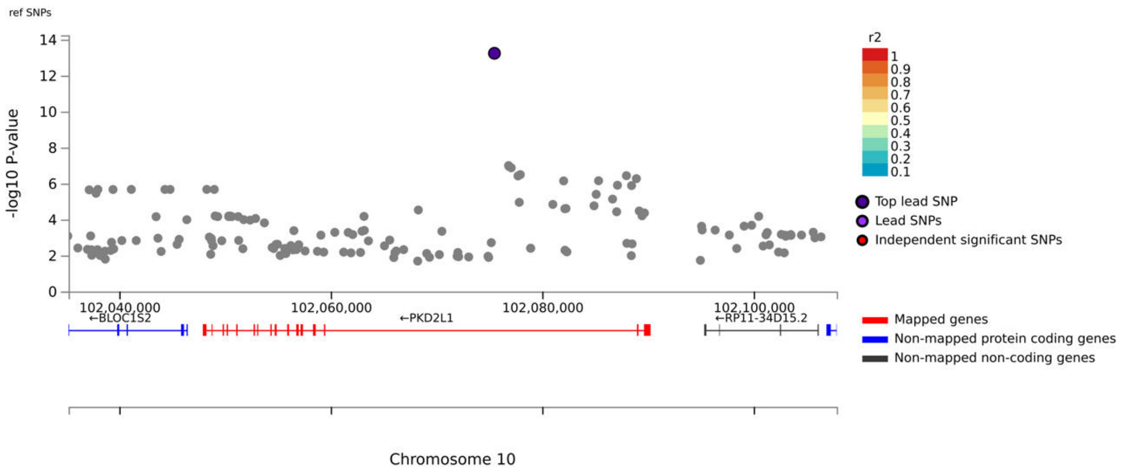
ref SNPs
-log10 P-value
r2
1
0.9
0.8
0.7
0.6
0.5
0.4
0.3
0.2
0.1
Top lead SNP
Lead SNPs
Independent significant SNPs
102,040,000
102,060,000
102,080,000
102,100,000
←BLOC1S2
←PKD2L1
←RP11-34D15.2
Mapped genes
Non-mapped protein coding genes
Non-mapped non-coding genes
Chromosome 10

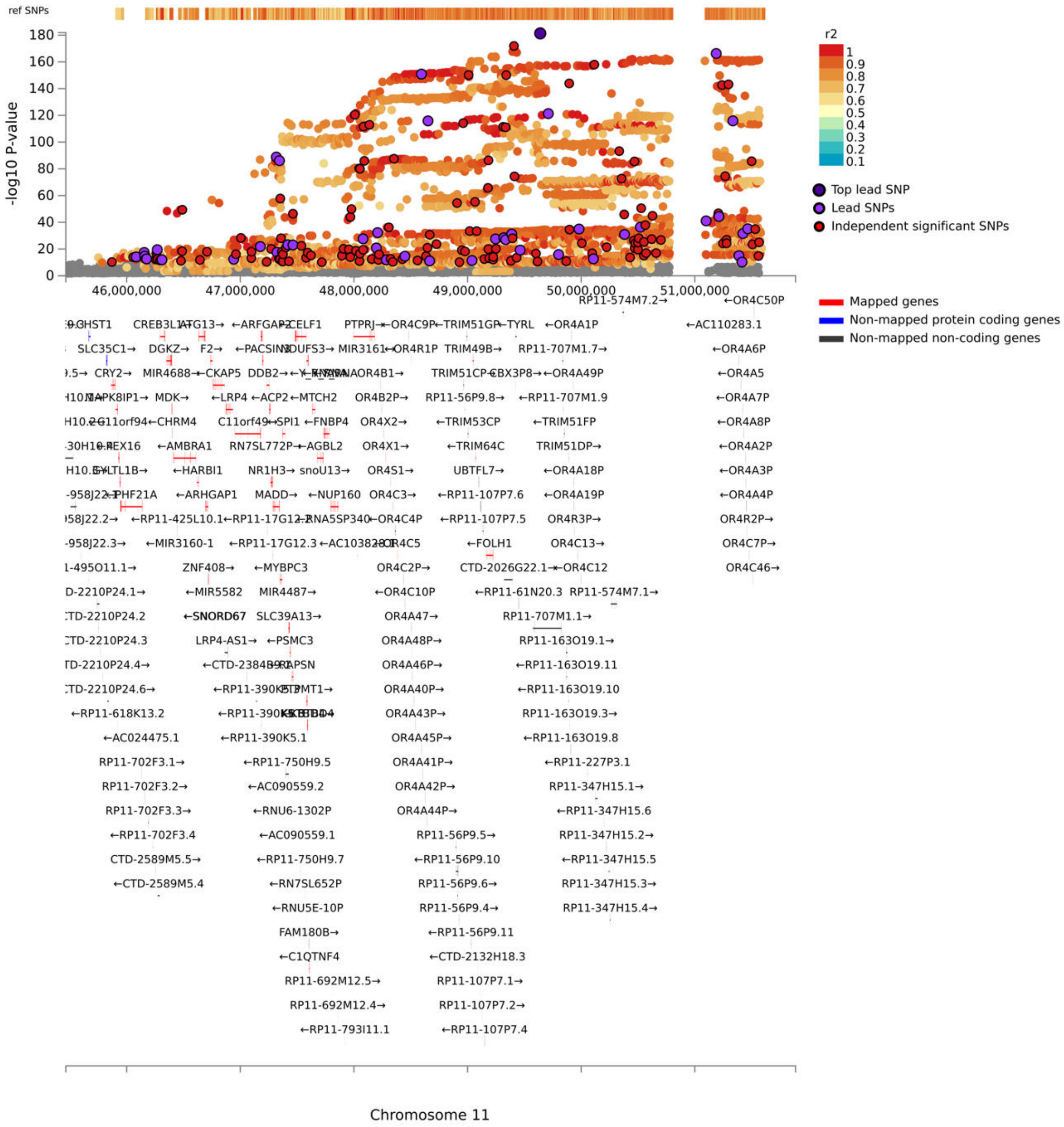

ref SNPs
-log10 P-value
0
20
40
60
80
100
120
140
160
180
46,000,000
47,000,000
48,000,000
49,000,000
50,000,000
51,000,000
r2
1
0.9
0.8
0.7
0.6
0.5
0.4
0.3
0.2
0.1
Top lead SNP
Lead SNPs
Independent significant SNPs
Mapped genes
Non-mapped protein coding genes
Non-mapped non-coding genes
Chromosome 11

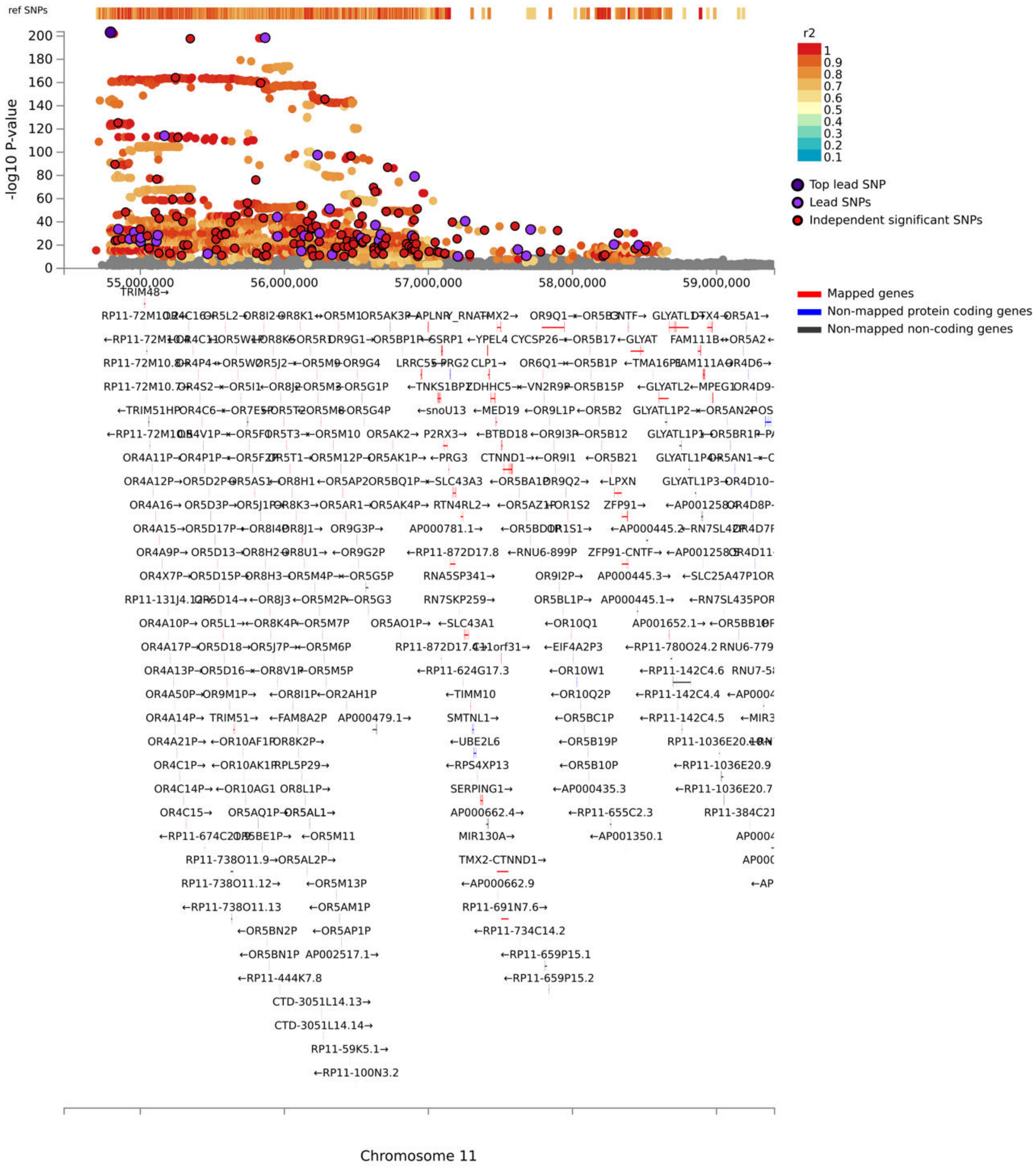

ref SNPs
-log10 P-value
0
20
40
60
80
100
120
140
160
180
200
55,000,000
56,000,000
57,000,000
58,000,000
59,000,000
Chromosome 11
r2
1
0.9
0.8
0.7
0.6
0.5
0.4
0.3
0.2
0.1
Top lead SNP
Lead SNPs
Independent significant SNPs
Mapped genes
Non-mapped protein coding genes
Non-mapped non-coding genes

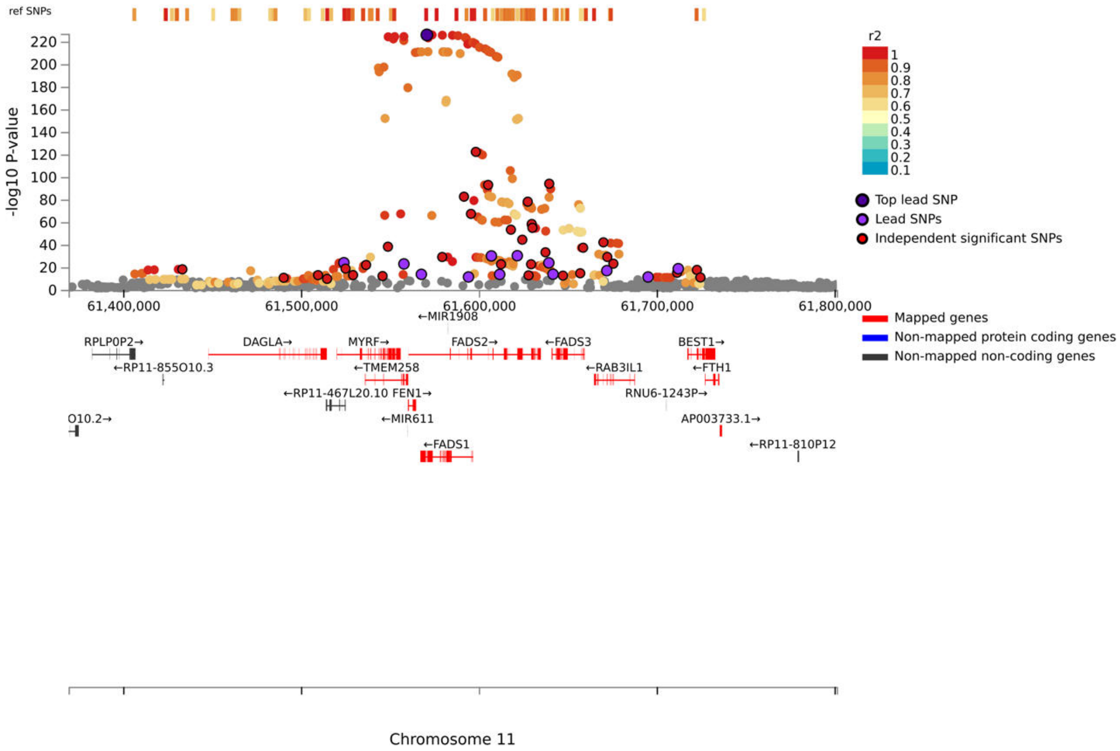
ref SNPs
-log10 P-value
r2
1
0.9
0.8
0.7
0.6
0.5
0.4
0.3
0.2
0.1
Top lead SNP
Lead SNPs
Independent significant SNPs
Mapped genes
Non-mapped protein coding genes
Non-mapped non-coding genes
61,400,000
61,500,000
61,600,000
61,700,000
61,800,000
←MIR1908
RPLP0P2→
DAGLA→
MYRF→
FADS2→
←FADS3
BEST1→
←RP11-855O10.3
←TMEM258
←RAB3IL1
←FTH1
←RP11-467L20.10
FEN1→
RNU6-1243P→
O10.2→
←MIR611
AP003733.1→
←FADS1
←RP11-810P12
Chromosome 11

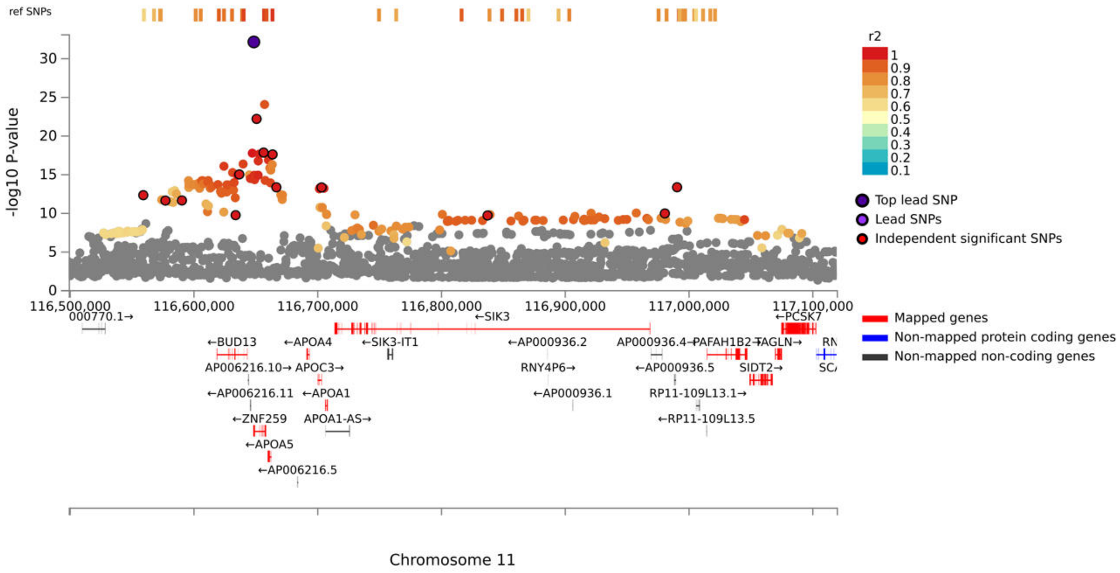
ref SNPs
-log10 P-value
r2
1
0.9
0.8
0.7
0.6
0.5
0.4
0.3
0.2
0.1
Top lead SNP
Lead SNPs
Independent significant SNPs
Mapped genes
Non-mapped protein coding genes
Non-mapped non-coding genes
116,500,000
116,600,000
116,700,000
116,800,000
116,900,000
117,000,000
117,100,000
000770.1→
←SIK3
←PCSK7
←BUD13
←APOA4
←SIK3-IT1
←AP000936.2
AP000936.4→
PAFAH1B2→
TAGLN→
AP006216.10→
APOC3→
RNY4P6→
←AP000936.5
SIDT2→
←AP006216.11
←APOA1
←AP000936.1
RP11-109L13.1→
←ZNF259
APOA1-AS→
←RP11-109L13.5
←APOA5
←AP006216.5
Chromosome 11

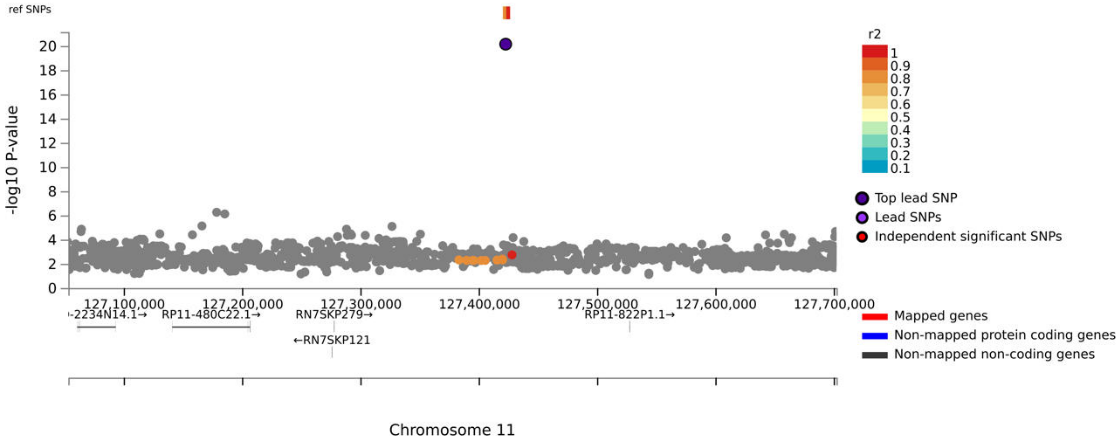
ref SNPs
-log10 P-value
r2
1
0.9
0.8
0.7
0.6
0.5
0.4
0.3
0.2
0.1
Top lead SNP
Lead SNPs
Independent significant SNPs
127,100,000
127,200,000
127,300,000
127,400,000
127,500,000
127,600,000
127,700,000
RP11-480C22.1→
RN7SKP279→
←RN7SKP121
RP11-822P1.1→
Mapped genes
Non-mapped protein coding genes
Non-mapped non-coding genes
Chromosome 11

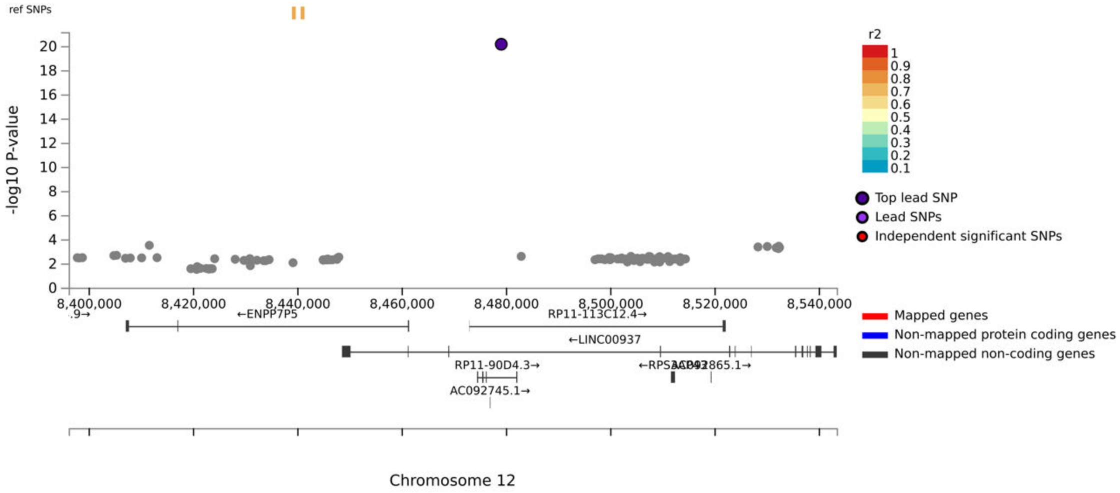
ref SNPs
-log10 P-value
r2
Top lead SNP
Lead SNPs
Independent significant SNPs
8,400,000
8,420,000
8,440,000
8,460,000
8,480,000
8,500,000
8,520,000
8,540,000
←ENPP7P5
RP11-113C12.4→
←LINC00937
RP11-90D4.3→
AC092745.1→
Mapped genes
Non-mapped protein coding genes
Non-mapped non-coding genes
Chromosome 12

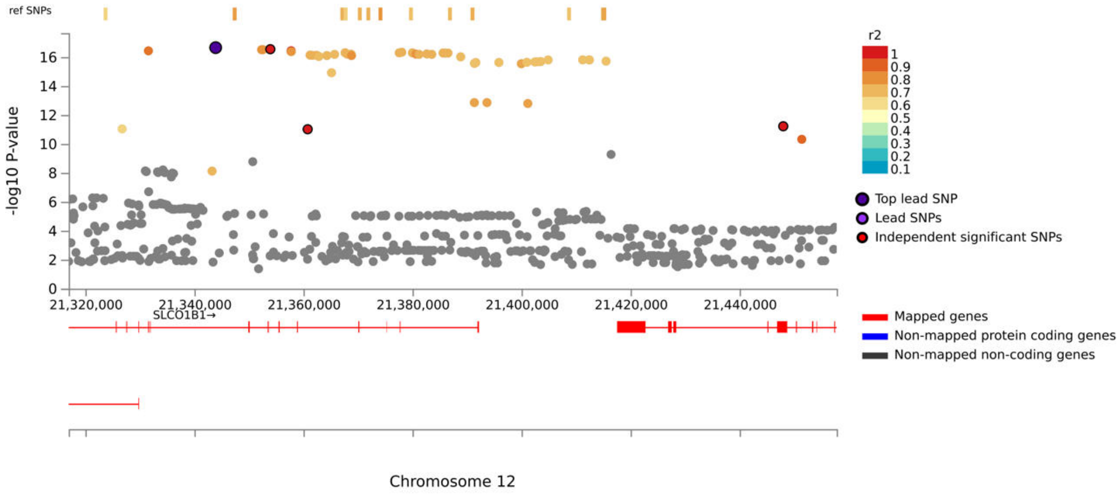
ref SNPs
-log10 P-value
r2
Top lead SNP
Lead SNPs
Independent significant SNPs
21,320,000
21,340,000
21,360,000
21,380,000
21,400,000
21,420,000
21,440,000
SLCO1B1→
Mapped genes
Non-mapped protein coding genes
Non-mapped non-coding genes
Chromosome 12

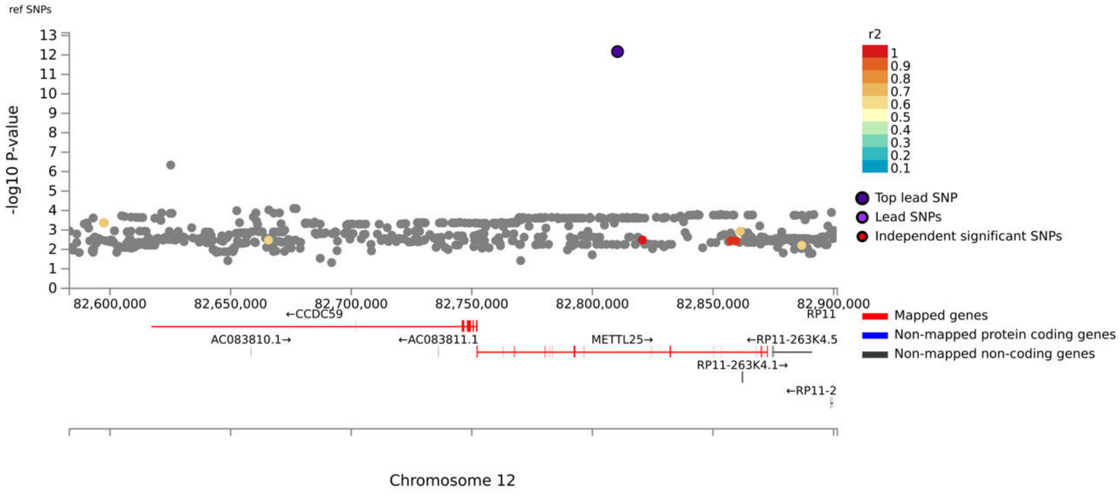
ref SNPs
-log10 P-value
0
1
2
3
4
5
6
7
8
9
10
11
12
13
82,600,000
82,650,000
82,700,000
82,750,000
82,800,000
82,850,000
82,900,000
r2
1
0.9
0.8
0.7
0.6
0.5
0.4
0.3
0.2
0.1
Top lead SNP
Lead SNPs
Independent significant SNPs
←CCDC59
AC083810.1→
←AC083811.1
METTL25→
←RP11-263K4.5
RP11-263K4.1→
←RP11-2
RP11
Mapped genes
Non-mapped protein coding genes
Non-mapped non-coding genes
Chromosome 12

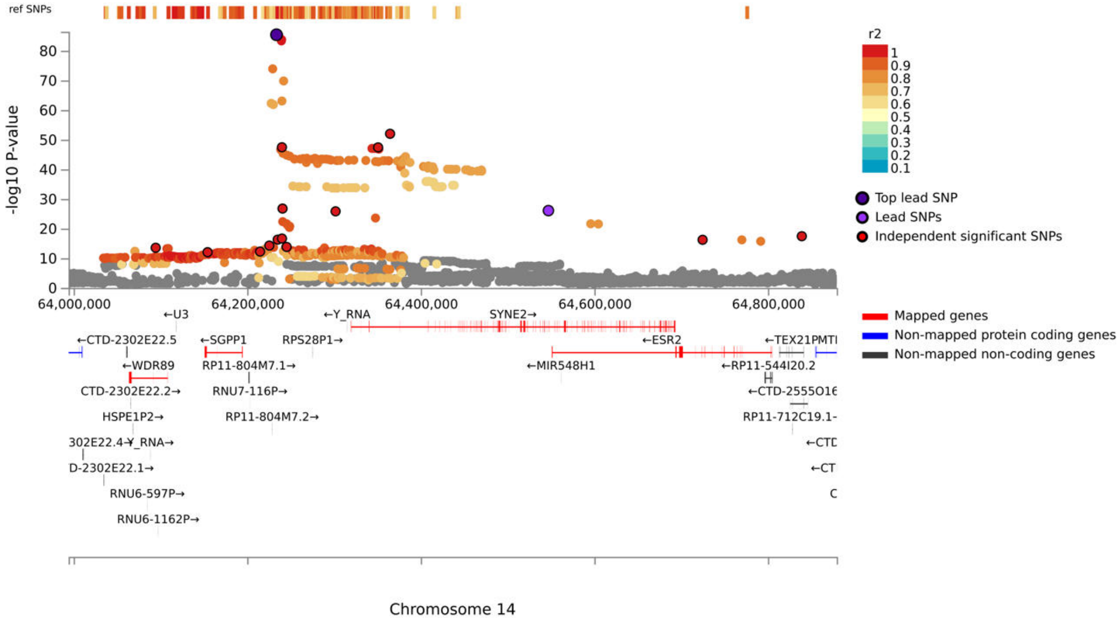
ref SNPs
-log10 P-value
0
10
20
30
40
50
60
70
80
64,000,000
64,200,000
64,400,000
64,600,000
64,800,000
r2
1
0.9
0.8
0.7
0.6
0.5
0.4
0.3
0.2
0.1
Top lead SNP
Lead SNPs
Independent significant SNPs
←U3
←Y_RNA
SYNE2→
←CTD-2302E22.5
←SGPP1
RPS28P1→
←ESR2
←TEX21PMTI
←WDR89
RP11-804M7.1→
←MIR548H1
←RP11-544I20.2
CTD-2302E22.2→
RNU7-116P→
←CTD-2555O16
HSPE1P2→
RP11-804M7.2→
RP11-712C19.1-
302E22.4→
Y_RNA→
←CTD
D-2302E22.1→
←CT
RNU6-597P→
C
RNU6-1162P→
Mapped genes
Non-mapped protein coding genes
Non-mapped non-coding genes
Chromosome 14

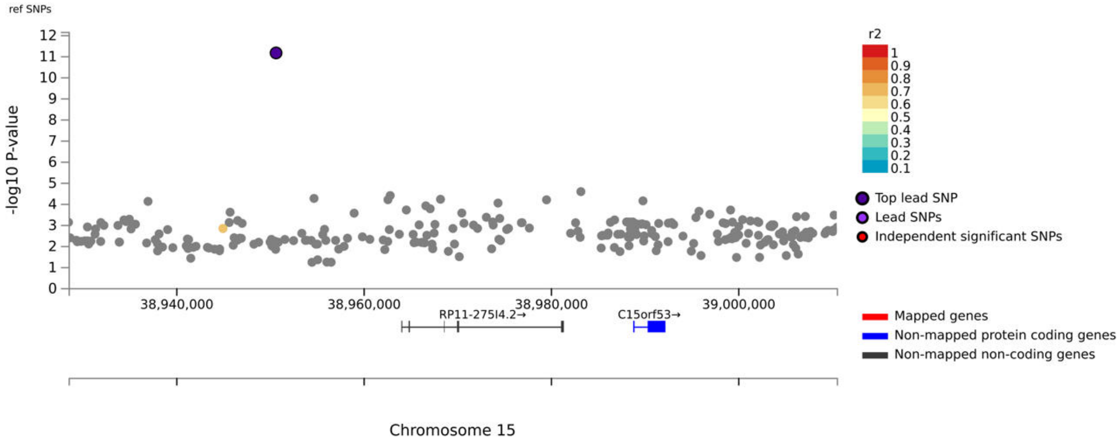
ref SNPs
-log10 P-value
38,940,000
38,960,000
38,980,000
39,000,000
RP11-275I4.2→
C15orf53→
r2
Top lead SNP
Lead SNPs
Independent significant SNPs
Mapped genes
Non-mapped protein coding genes
Non-mapped non-coding genes
Chromosome 15

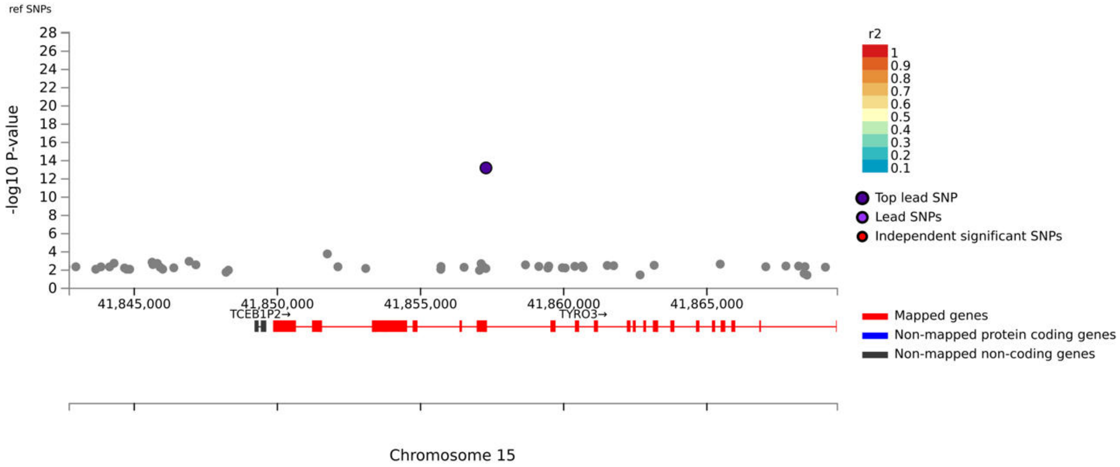
ref SNPs
-log10 P-value
41,845,000
41,850,000
41,855,000
41,860,000
41,865,000
TCEB1P2→
TYRO3→
r2
Top lead SNP
Lead SNPs
Independent significant SNPs
Mapped genes
Non-mapped protein coding genes
Non-mapped non-coding genes
Chromosome 15

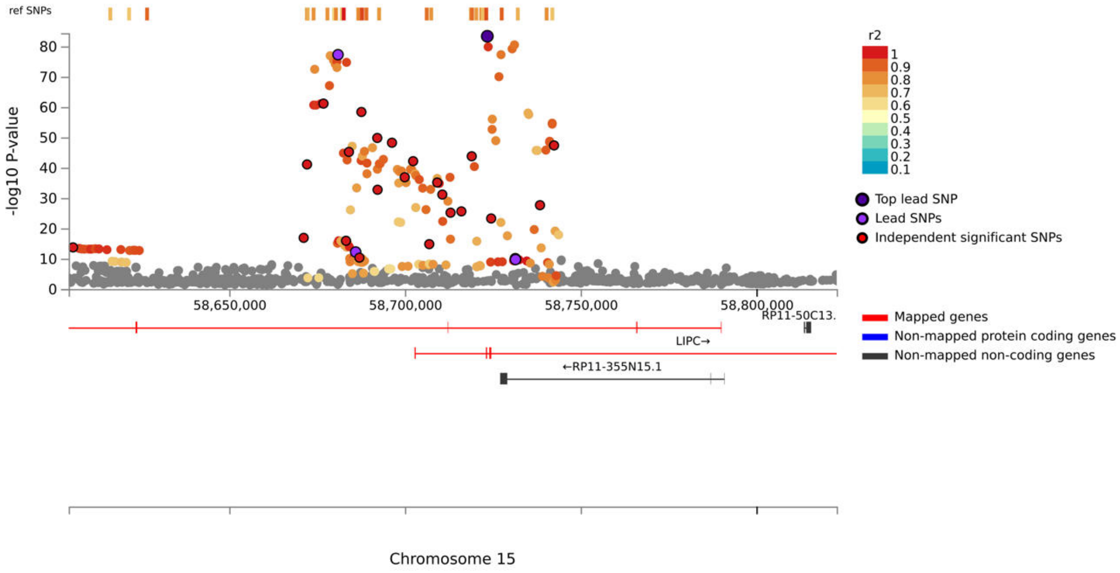
ref SNPs
-log10 P-value
58,650,000
58,700,000
58,750,000
58,800,000
RP11-50C13.
LIPC→
←RP11-355N15.1
r2
Top lead SNP
Lead SNPs
Independent significant SNPs
Mapped genes
Non-mapped protein coding genes
Non-mapped non-coding genes
Chromosome 15

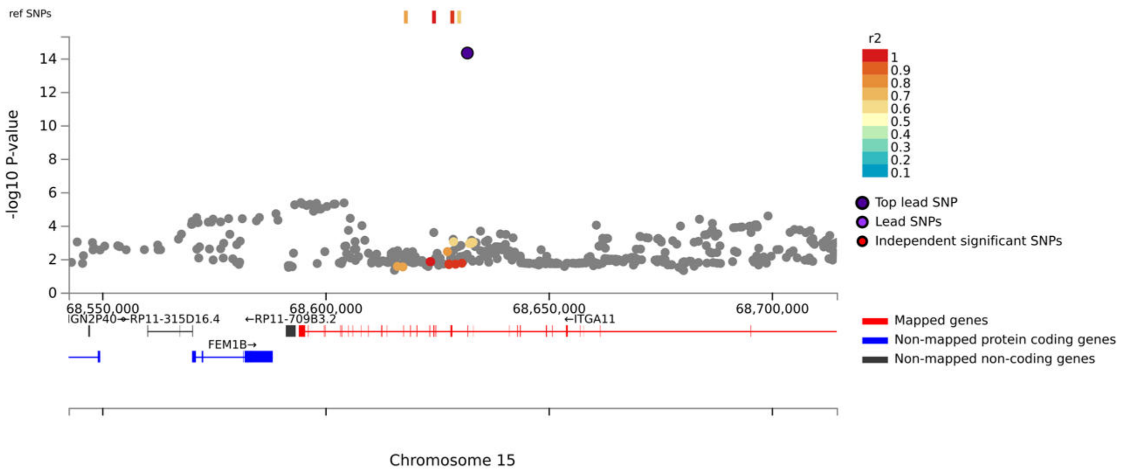
ref SNPs
-log10 P-value
r2
1
0.9
0.8
0.7
0.6
0.5
0.4
0.3
0.2
0.1
Top lead SNP
Lead SNPs
Independent significant SNPs
68,550,000
68,600,000
68,650,000
68,700,000
←RP11-709B3.2
←ITGA11
FEM1B→
Mapped genes
Non-mapped protein coding genes
Non-mapped non-coding genes
Chromosome 15

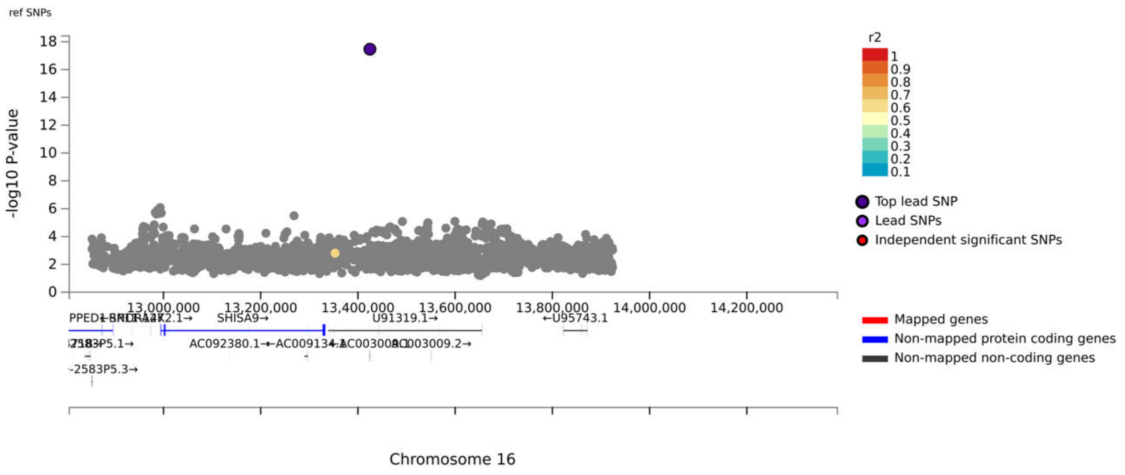
ref SNPs
-log10 P-value
r2
1
0.9
0.8
0.7
0.6
0.5
0.4
0.3
0.2
0.1
Top lead SNP
Lead SNPs
Independent significant SNPs
13,000,000
13,200,000
13,400,000
13,600,000
13,800,000
14,000,000
14,200,000
SHISA9→
U91319.1→
←U95743.1
Mapped genes
Non-mapped protein coding genes
Non-mapped non-coding genes
Chromosome 16

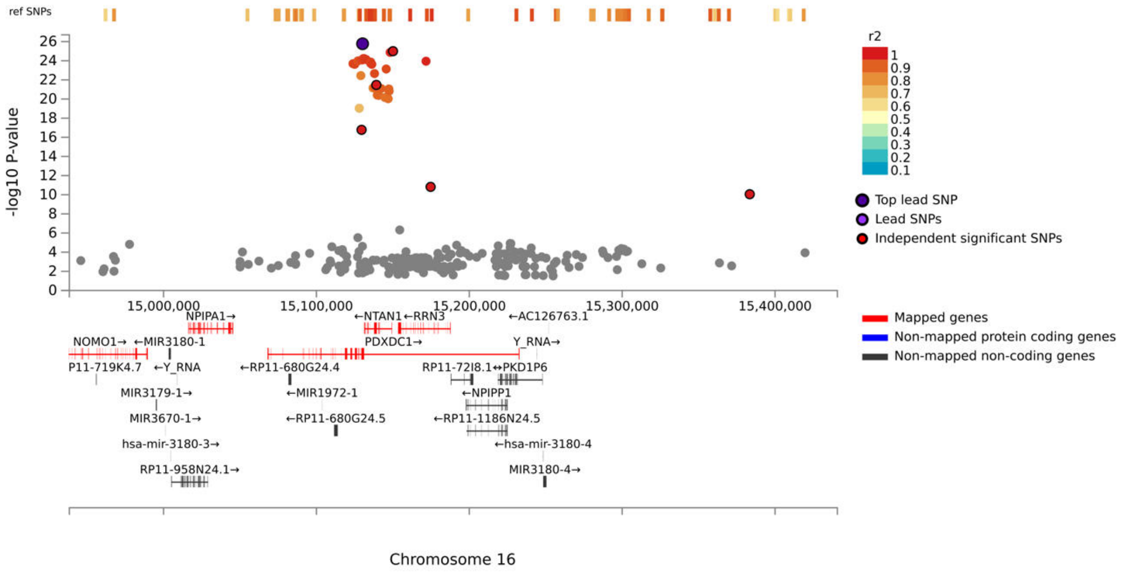
ref SNPs
-log10 P-value
r2
1
0.9
0.8
0.7
0.6
0.5
0.4
0.3
0.2
0.1
Top lead SNP
Lead SNPs
Independent significant SNPs
15,000,000
15,100,000
15,200,000
15,300,000
15,400,000
NPIPA1→
←NTAN1
←RRN3
←AC126763.1
NOMO1→
←MIR3180-1
PDXDC1→
Y_RNA→
←Y_RNA
←RP11-680G24.4
RP11-72I8.1
↔PKD1P6
MIR3179-1→
←MIR1972-1
←NPIPP1
MIR3670-1→
←RP11-680G24.5
←RP11-1186N24.5
hsa-mir-3180-3→
←hsa-mir-3180-4
RP11-958N24.1→
MIR3180-4→
Mapped genes
Non-mapped protein coding genes
Non-mapped non-coding genes
Chromosome 16

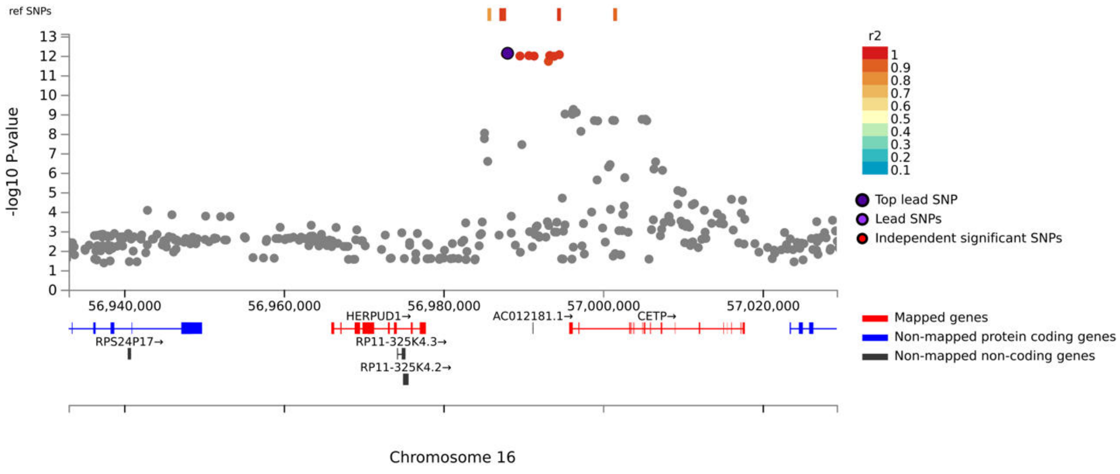
ref SNPs
-log10 P-value
r2
Top lead SNP
Lead SNPs
Independent significant SNPs
56,940,000
56,960,000
56,980,000
57,000,000
57,020,000
HERPUD1→
AC012181.1→
CETP→
RPS24P17→
RP11-325K4.3→
RP11-325K4.2→
Mapped genes
Non-mapped protein coding genes
Non-mapped non-coding genes
Chromosome 16

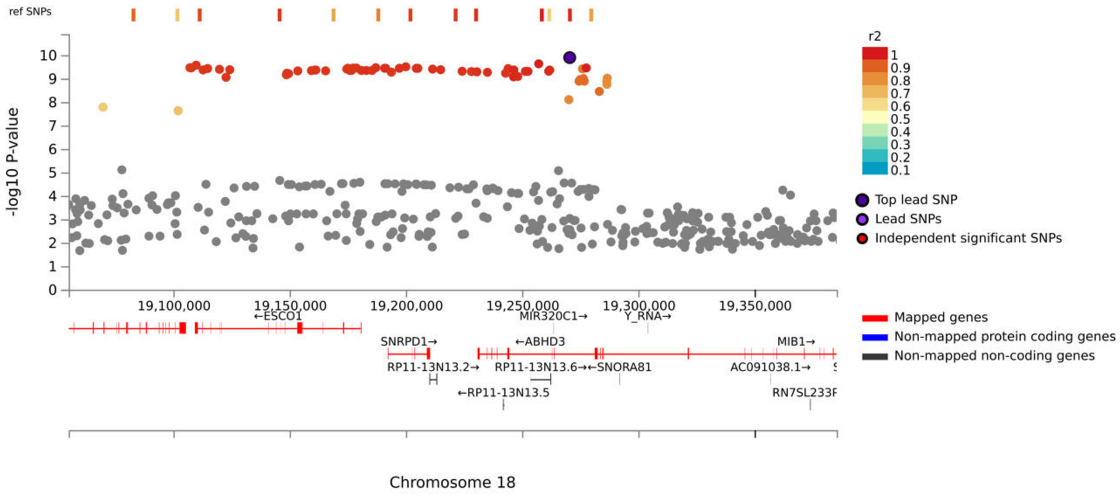
ref SNPs
-log10 P-value
r2
Top lead SNP
Lead SNPs
Independent significant SNPs
19,100,000
19,150,000
19,200,000
19,250,000
19,300,000
19,350,000
←ESCO1
MIR320C1→
Y_RNA→
SNRPD1→
←ABHD3
MIB1→
RP11-13N13.2→
RP11-13N13.6→
←SNORA81
AC091038.1→
←RP11-13N13.5
Mapped genes
Non-mapped protein coding genes
Non-mapped non-coding genes
Chromosome 18

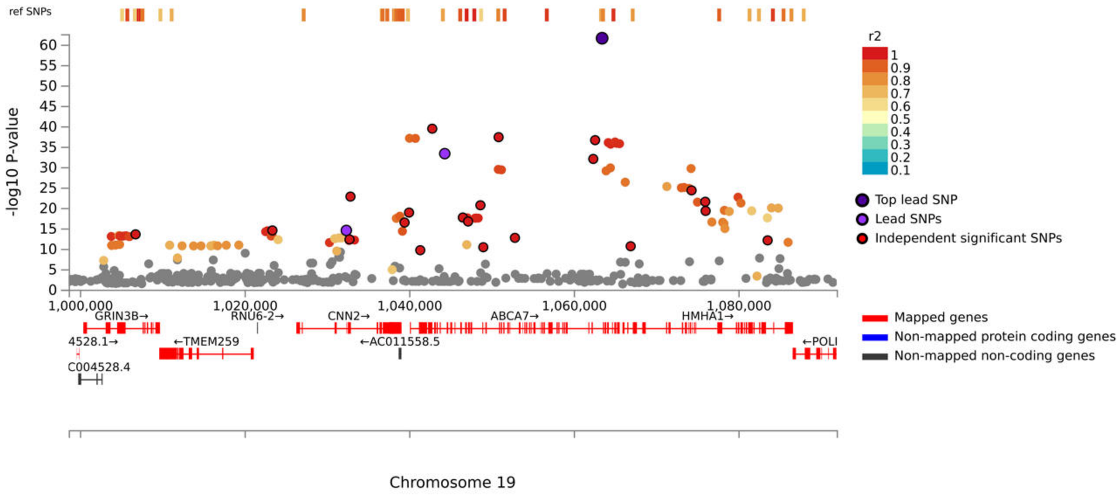
ref SNPs
-log10 P-value
r2
Top lead SNP
Lead SNPs
Independent significant SNPs
1,000,000
1,020,000
1,040,000
1,060,000
1,080,000
GRIN3B→
RNU6-2→
CNN2→
ABCA7→
HMHA1→
←TMEM259
←AC011558.5
Mapped genes
Non-mapped protein coding genes
Non-mapped non-coding genes
Chromosome 19

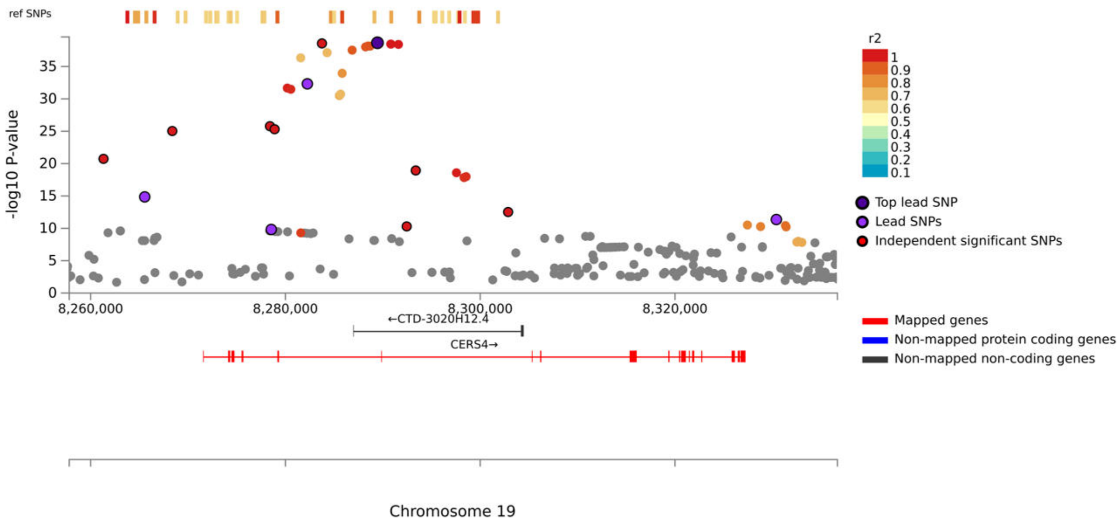
ref SNPs
-log10 P-value
r2
Top lead SNP
Lead SNPs
Independent significant SNPs
8,260,000
8,280,000
8,300,000
8,320,000
←CTD-3020H12.4
CERS4→
Mapped genes
Non-mapped protein coding genes
Non-mapped non-coding genes
Chromosome 19

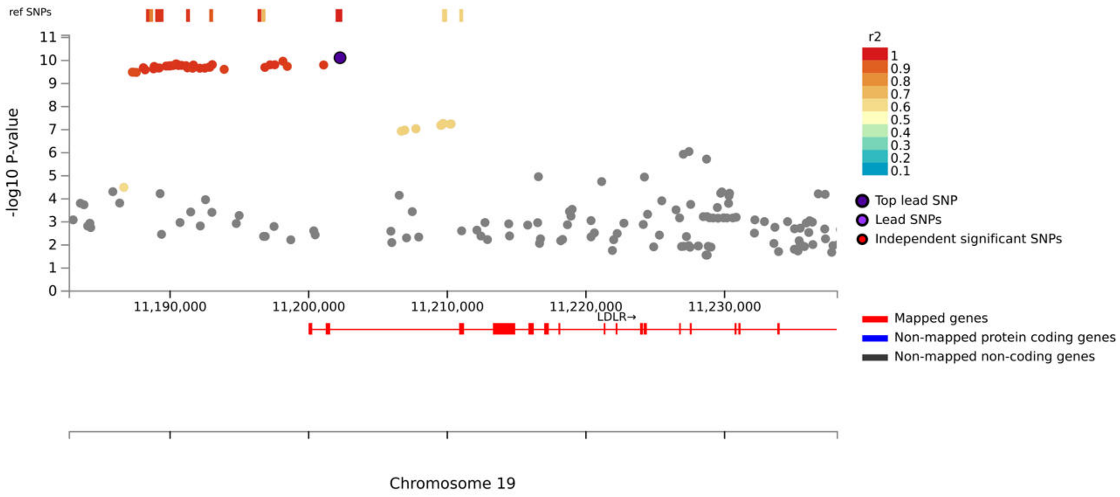
ref SNPs
-log10 P-value
r2
Top lead SNP
Lead SNPs
Independent significant SNPs
11,190,000
11,200,000
11,210,000
11,220,000
11,230,000
LDLR→
Mapped genes
Non-mapped protein coding genes
Non-mapped non-coding genes
Chromosome 19

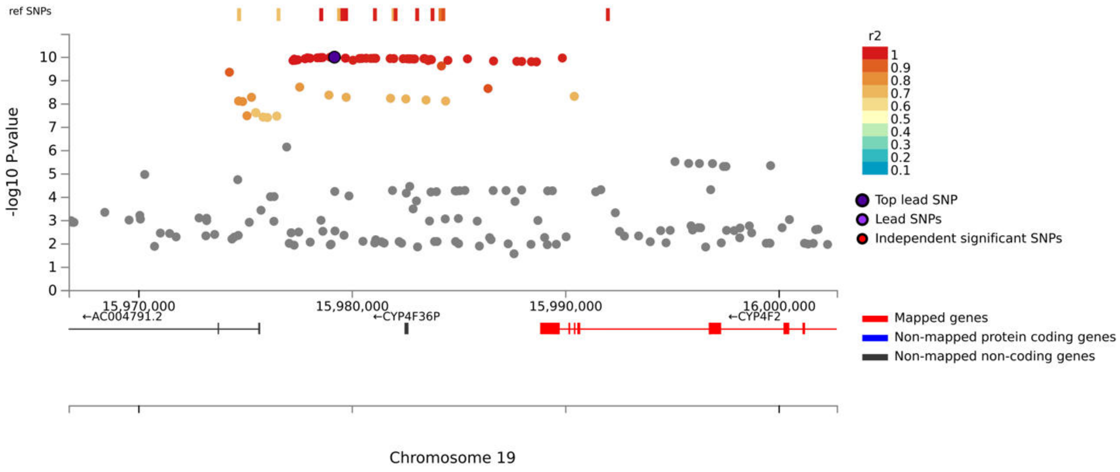
ref SNPs
-log10 P-value
r2
Top lead SNP
Lead SNPs
Independent significant SNPs
15,970,000
15,980,000
15,990,000
16,000,000
←AC004791.2
←CYP4F36P
←CYP4F2
Mapped genes
Non-mapped protein coding genes
Non-mapped non-coding genes
Chromosome 19

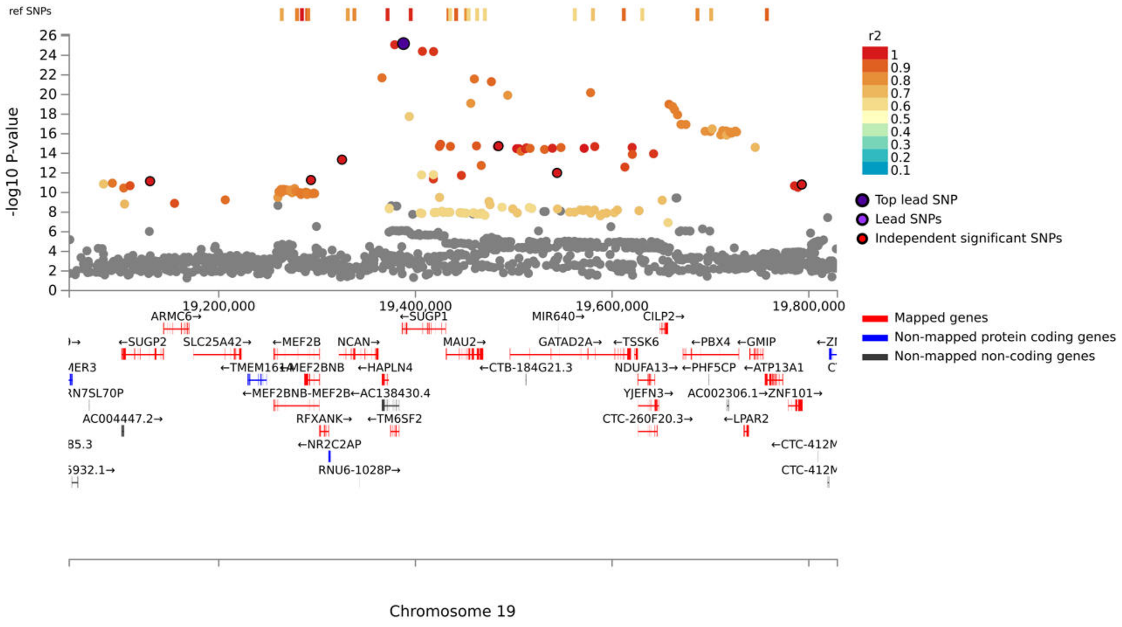
ref SNPs
-log10 P-value
r2
1
0.9
0.8
0.7
0.6
0.5
0.4
0.3
0.2
0.1
Top lead SNP
Lead SNPs
Independent significant SNPs
19,200,000
19,400,000
19,600,000
19,800,000
Mapped genes
Non-mapped protein coding genes
Non-mapped non-coding genes
ARMC6→
←SUGP2
SLC25A42→
←MEF2B
NCAN→
←SUGP1
MAU2→
MIR640→
GATAD2A→
CILP2→
←TSSK6
←PBX4
←GMIP
←TMEM161A
←MEF2BNB
←HAPLN4
←CTB-184G21.3
NDUFA13→
←PHF5CP
←ATP13A1
←MEF2BNB-MEF2B
←AC138430.4
YJEFN3→
AC002306.1→
ZNF101→
AC004447.2→
RFXANK→
←TM6SF2
CTC-260F20.3→
←LPAR2
←NR2C2AP
RNU6-1028P→
Chromosome 19

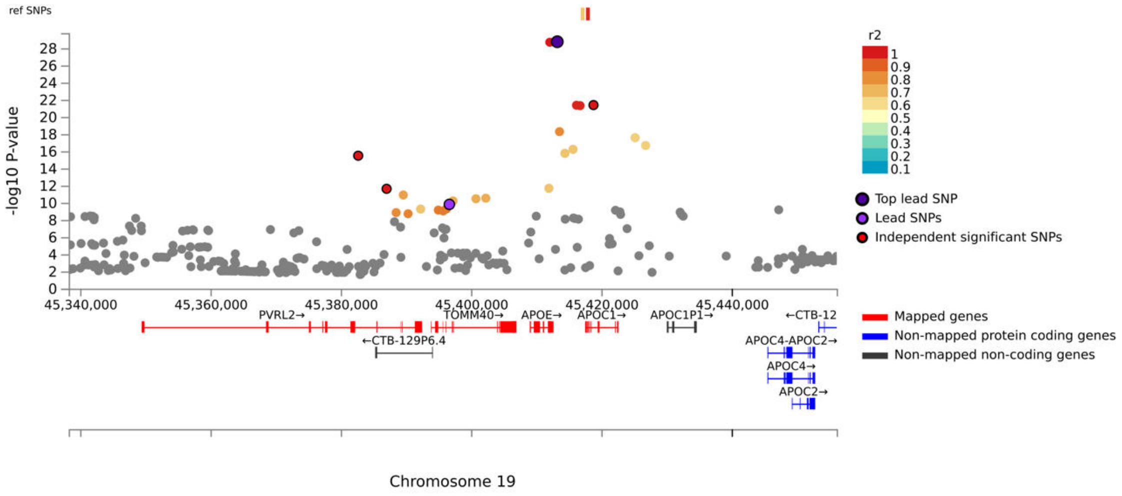
ref SNPs
-log10 P-value
r2
1
0.9
0.8
0.7
0.6
0.5
0.4
0.3
0.2
0.1
Top lead SNP
Lead SNPs
Independent significant SNPs
45,340,000
45,360,000
45,380,000
45,400,000
45,420,000
45,440,000
Mapped genes
Non-mapped protein coding genes
Non-mapped non-coding genes
PVRL2→
TOMM40→
APOE→
APOC1→
APOC1P1→
←CTB-129P6.4
APOC4-APOC2→
APOC4→
APOC2→
Chromosome 19

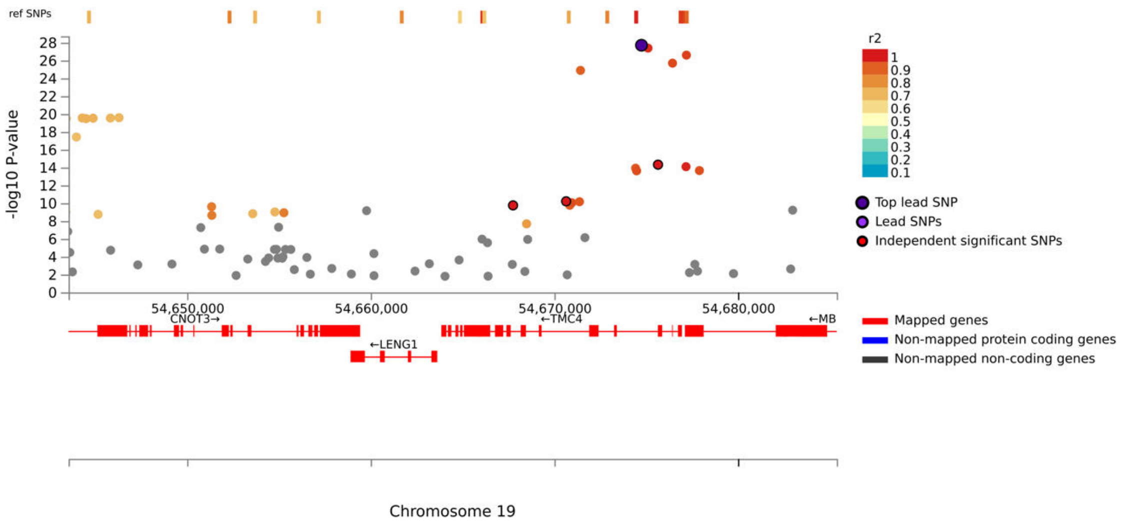

ref SNPs
-log10 P-value
54,650,000
54,660,000
54,670,000
54,680,000
CNOT3→
←TMC4
←MB
←LENG1
r2
Top lead SNP
Lead SNPs
Independent significant SNPs
Mapped genes
Non-mapped protein coding genes
Non-mapped non-coding genes
Chromosome 19

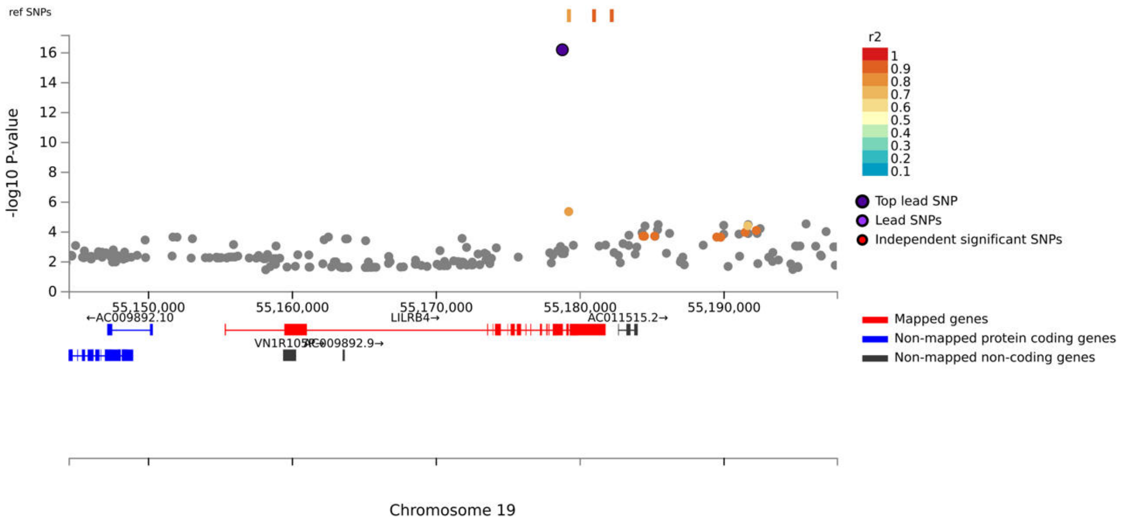

ref SNPs
-log10 P-value
55,150,000
55,160,000
55,170,000
55,180,000
55,190,000
←AC009892.10
LILRB4→
AC011515.2→
r2
Top lead SNP
Lead SNPs
Independent significant SNPs
Mapped genes
Non-mapped protein coding genes
Non-mapped non-coding genes
Chromosome 19

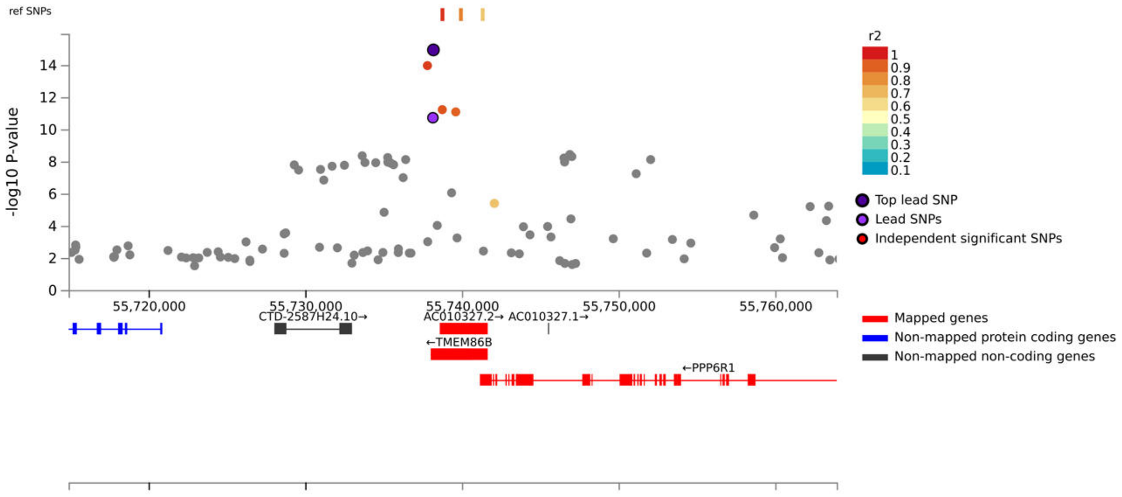

Chromosome 19

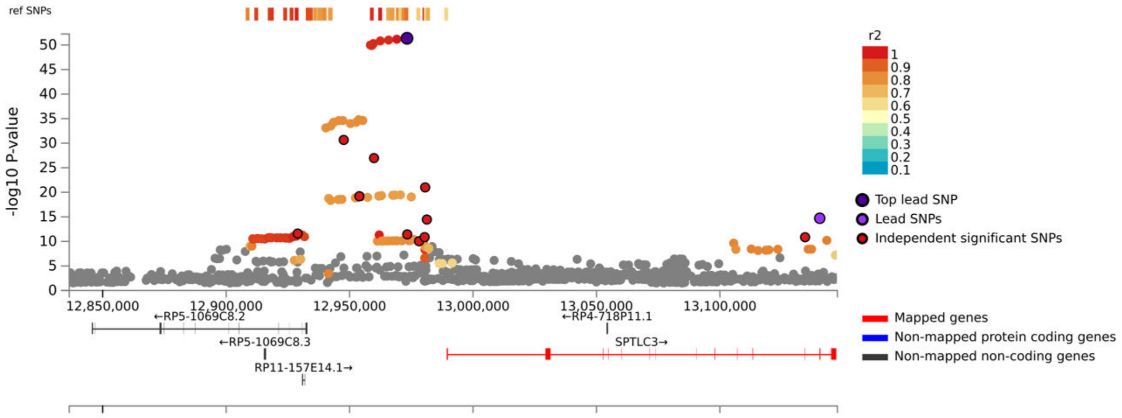

Chromosome 20

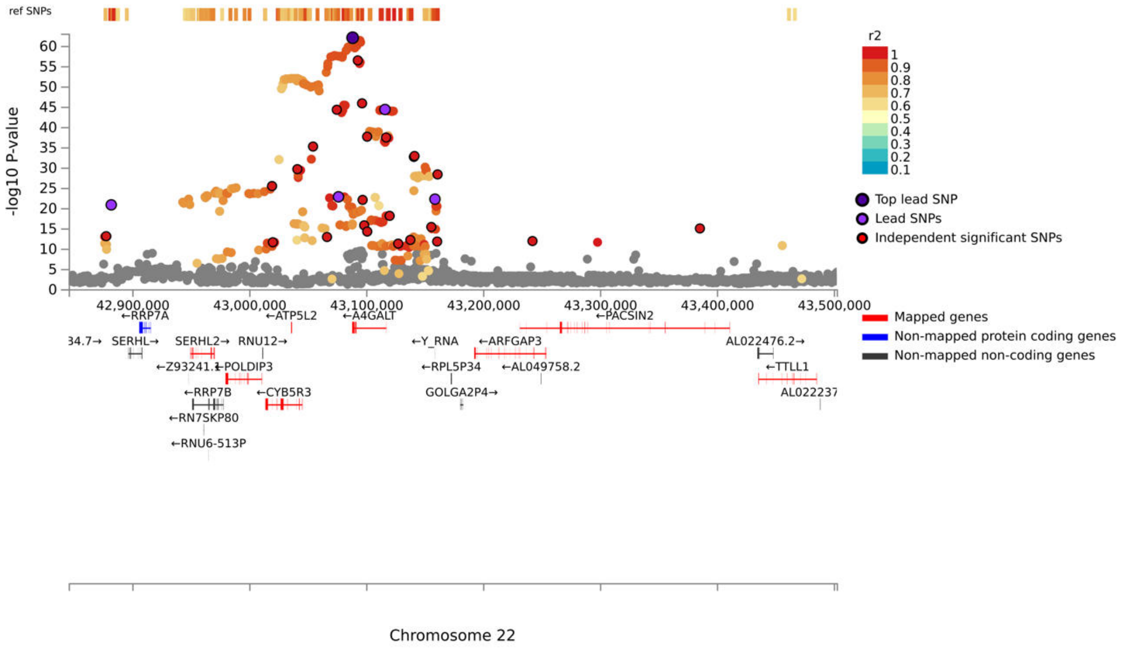
ref SNPs
-log10 P-value
r2
1
0.9
0.8
0.7
0.6
0.5
0.4
0.3
0.2
0.1
Top lead SNP
Lead SNPs
Independent significant SNPs
42,900,000
43,000,000
43,100,000
43,200,000
43,300,000
43,400,000
43,500,000
←RRP7A
←ATP5L2
←A4GALT
←PACSIN2
SERHL→
SERHL2→
RNU12→
←Y_RNA
←ARFGAP3
AL022476.2→
←Z93241.1
←POLDIP3
←RPL5P34
←AL049758.2
←TTLL1
←RRP7B
←CYB5R3
GOLGA2P4→
←RN7SKP80
←RNU6-513P
Mapped genes
Non-mapped protein coding genes
Non-mapped non-coding genes
Chromosome 22

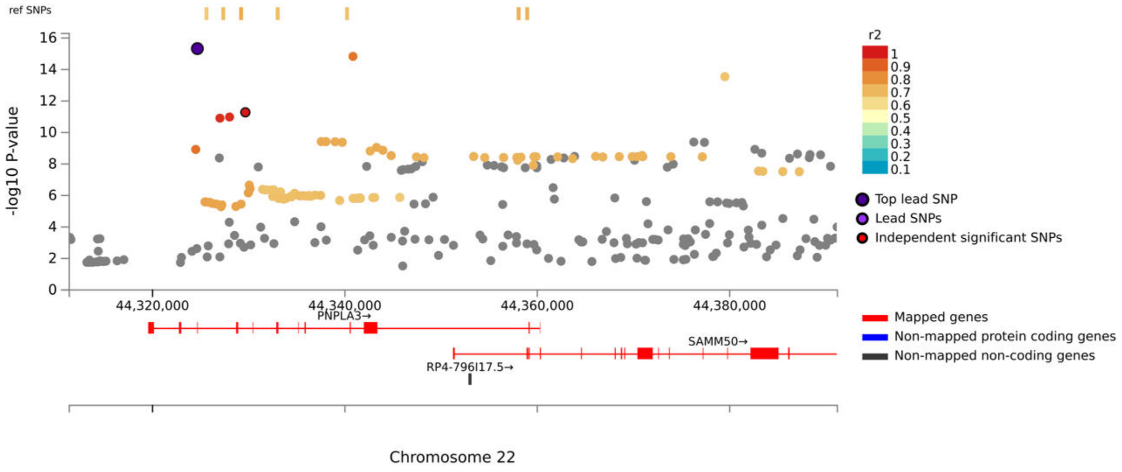
ref SNPs
-log10 P-value
r2
1
0.9
0.8
0.7
0.6
0.5
0.4
0.3
0.2
0.1
Top lead SNP
Lead SNPs
Independent significant SNPs
44,320,000
44,340,000
44,360,000
44,380,000
PNPLA3→
SAMM50→
RP4-796I17.5→
Mapped genes
Non-mapped protein coding genes
Non-mapped non-coding genes
Chromosome 22

(b)

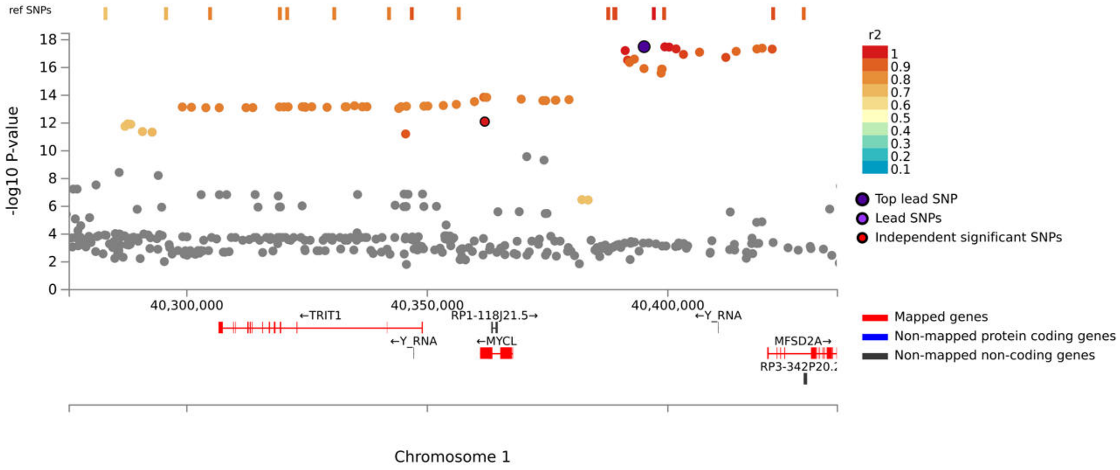
ref SNPs
-log10 P-value
r2
1
0.9
0.8
0.7
0.6
0.5
0.4
0.3
0.2
0.1
Top lead SNP
Lead SNPs
Independent significant SNPs
40,300,000
40,350,000
40,400,000
←TRIT1
RP1-118J21.5→
←Y_RNA
←MYCL
←Y_RNA
MFSD2A→
Mapped genes
Non-mapped protein coding genes
Non-mapped non-coding genes
Chromosome 1

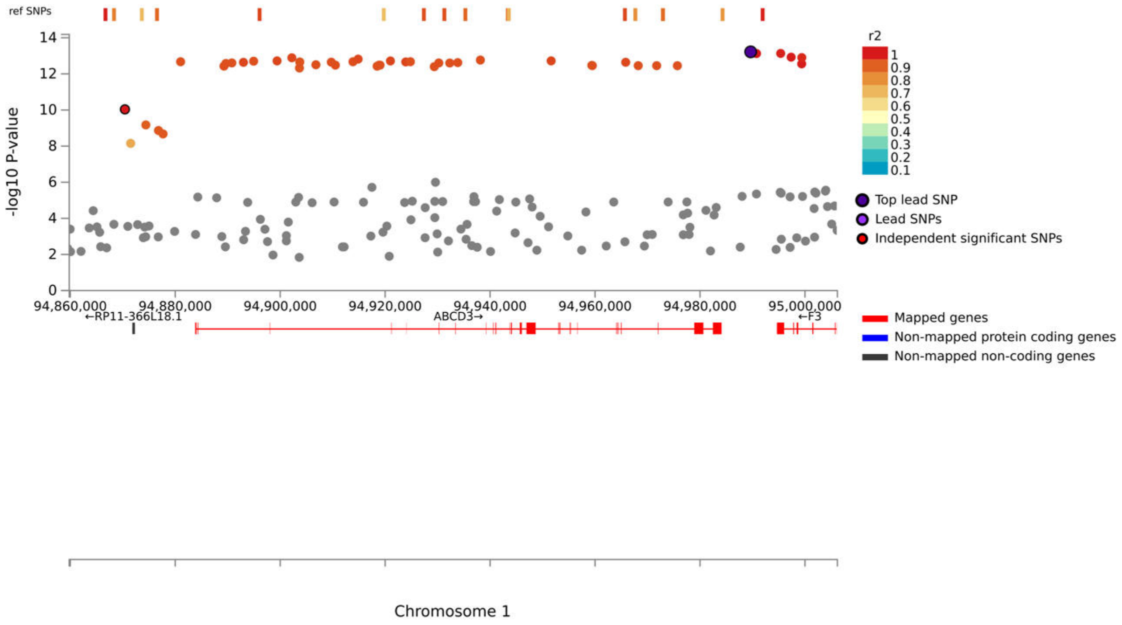
ref SNPs
-log10 P-value
0
2
4
6
8
10
12
14
94,860,000
94,880,000
94,900,000
94,920,000
94,940,000
94,960,000
94,980,000
95,000,000
←RP11-366L18.1
ABCD3→
←F3
r2
1
0.9
0.8
0.7
0.6
0.5
0.4
0.3
0.2
0.1
Top lead SNP
Lead SNPs
Independent significant SNPs
Mapped genes
Non-mapped protein coding genes
Non-mapped non-coding genes
Chromosome 1

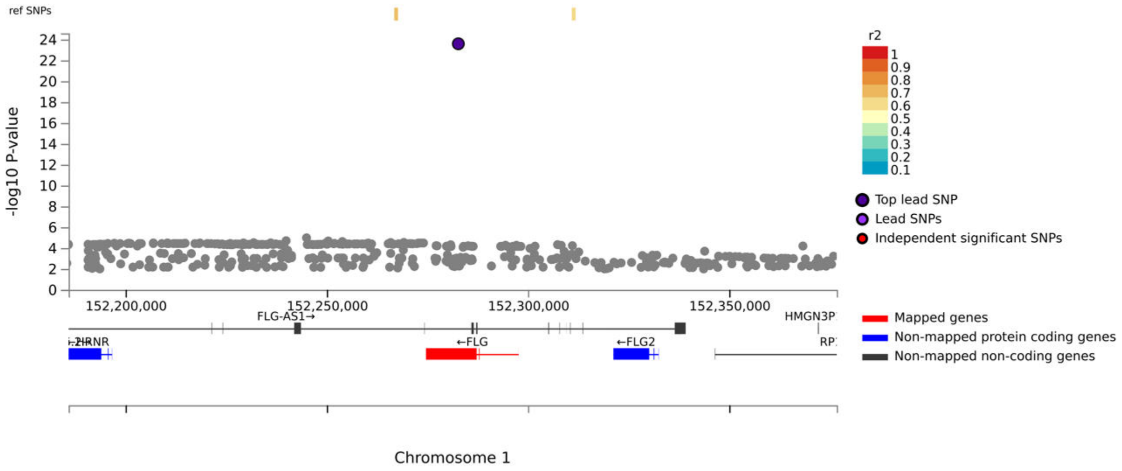
ref SNPs
-log10 P-value
0
2
4
6
8
10
12
14
16
18
20
22
24
152,200,000
152,250,000
152,300,000
152,350,000
FLG-AS1→
HMGN3P
←FLG
←FLG2
r2
1
0.9
0.8
0.7
0.6
0.5
0.4
0.3
0.2
0.1
Top lead SNP
Lead SNPs
Independent significant SNPs
Mapped genes
Non-mapped protein coding genes
Non-mapped non-coding genes
Chromosome 1

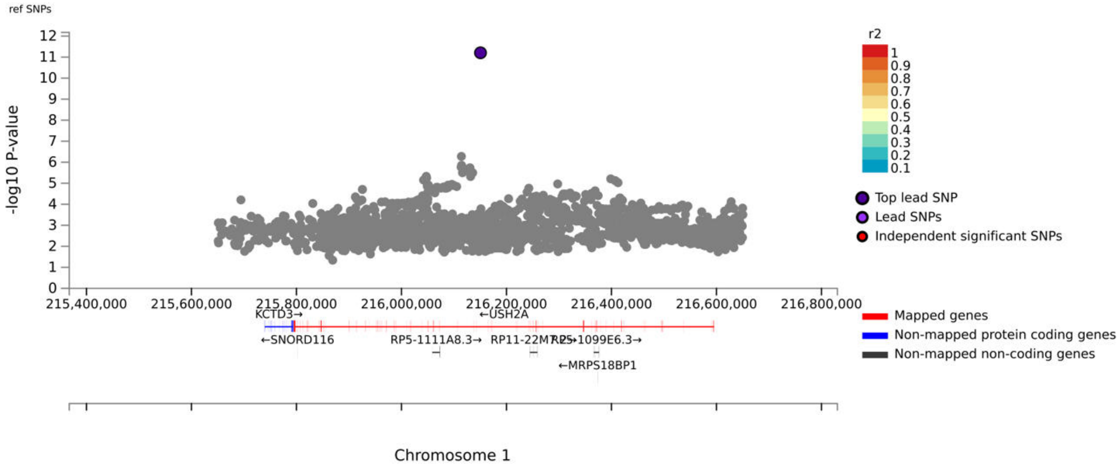
ref SNPs
-log10 P-value
r2
Top lead SNP
Lead SNPs
Independent significant SNPs
KCTD3→
←USH2A
←SNORD116
RP5-1111A8.3→
RP11-22M7.2→
RP5-1099E6.3→
←MRPS18BP1
Mapped genes
Non-mapped protein coding genes
Non-mapped non-coding genes
Chromosome 1

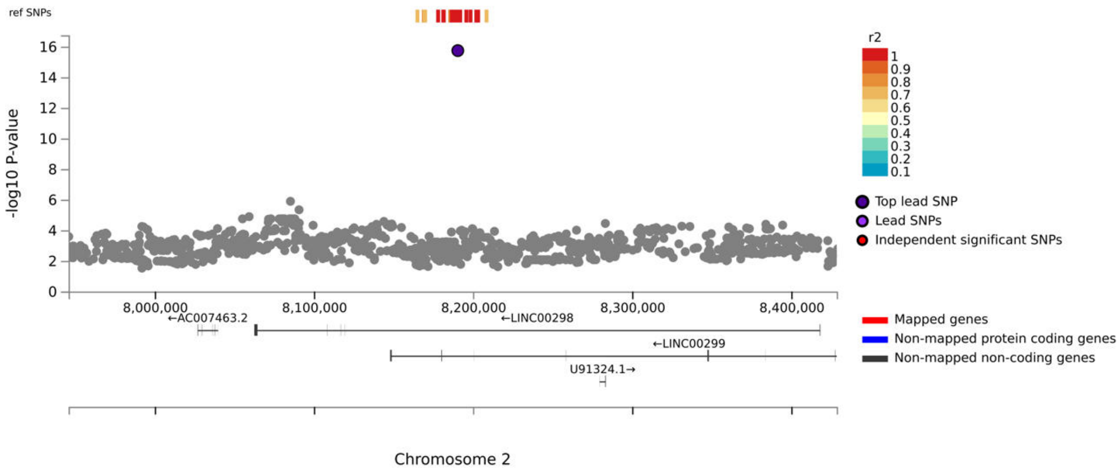
ref SNPs
-log10 P-value
r2
Top lead SNP
Lead SNPs
Independent significant SNPs
←AC007463.2
←LINC00298
←LINC00299
U91324.1→
Mapped genes
Non-mapped protein coding genes
Non-mapped non-coding genes
Chromosome 2

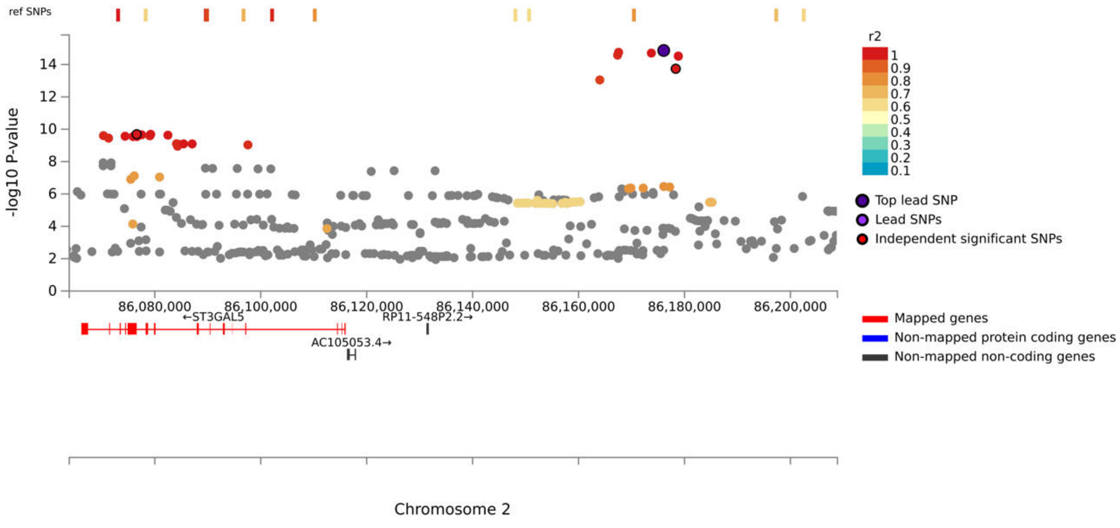
ref SNPs
-log10 P-value
r2
Top lead SNP
Lead SNPs
Independent significant SNPs
←ST3GAL5
RP11-548P2.2→
AC105053.4→
Mapped genes
Non-mapped protein coding genes
Non-mapped non-coding genes
Chromosome 2

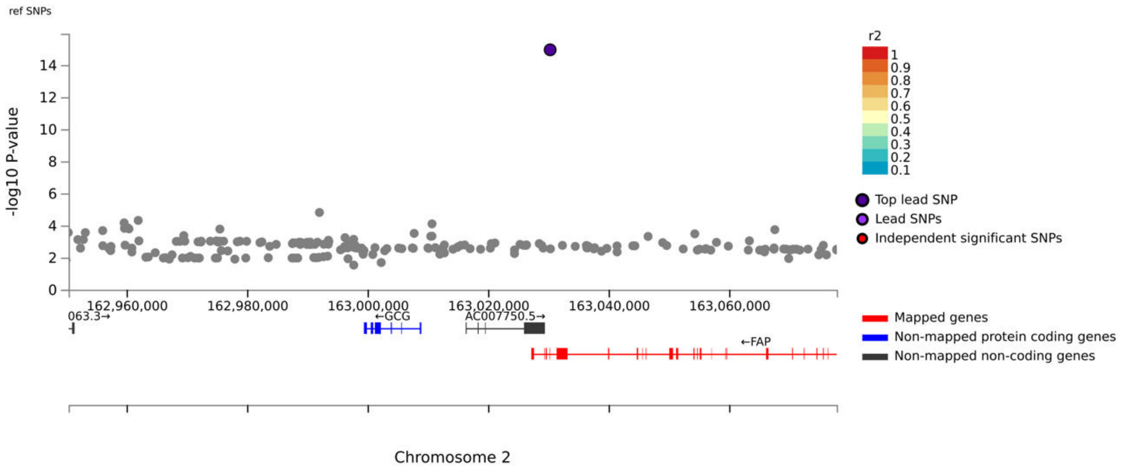

ref SNPs
-log10 P-value
0
2
4
6
8
10
12
14
162,960,000
162,980,000
163,000,000
163,020,000
163,040,000
163,060,000
063.3→
←GCG
AC007750.5→
←FAP
Chromosome 2
r2
1
0.9
0.8
0.7
0.6
0.5
0.4
0.3
0.2
0.1
Top lead SNP
Lead SNPs
Independent significant SNPs
Mapped genes
Non-mapped protein coding genes
Non-mapped non-coding genes

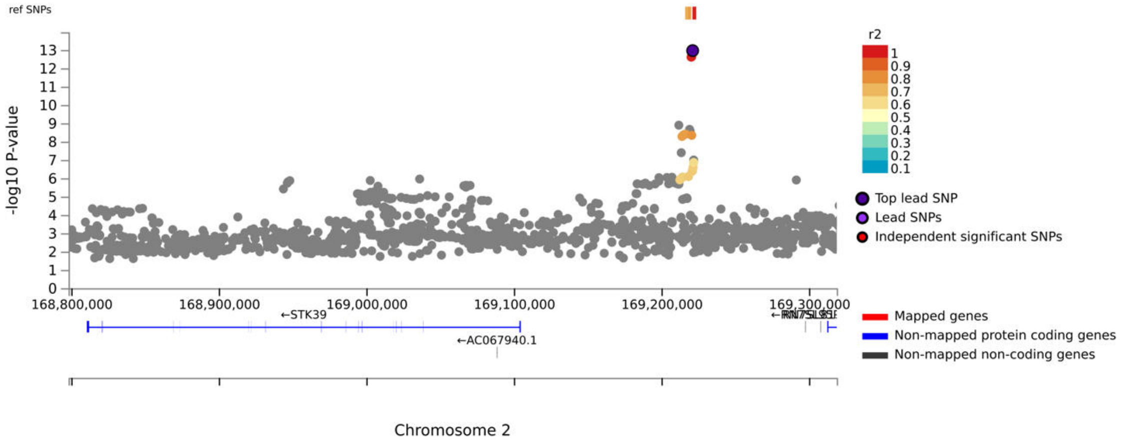

ref SNPs
-log10 P-value
0
1
2
3
4
5
6
7
8
9
10
11
12
13
168,800,000
168,900,000
169,000,000
169,100,000
169,200,000
169,300,000
←STK39
←AC067940.1
Chromosome 2
r2
1
0.9
0.8
0.7
0.6
0.5
0.4
0.3
0.2
0.1
Top lead SNP
Lead SNPs
Independent significant SNPs
Mapped genes
Non-mapped protein coding genes
Non-mapped non-coding genes

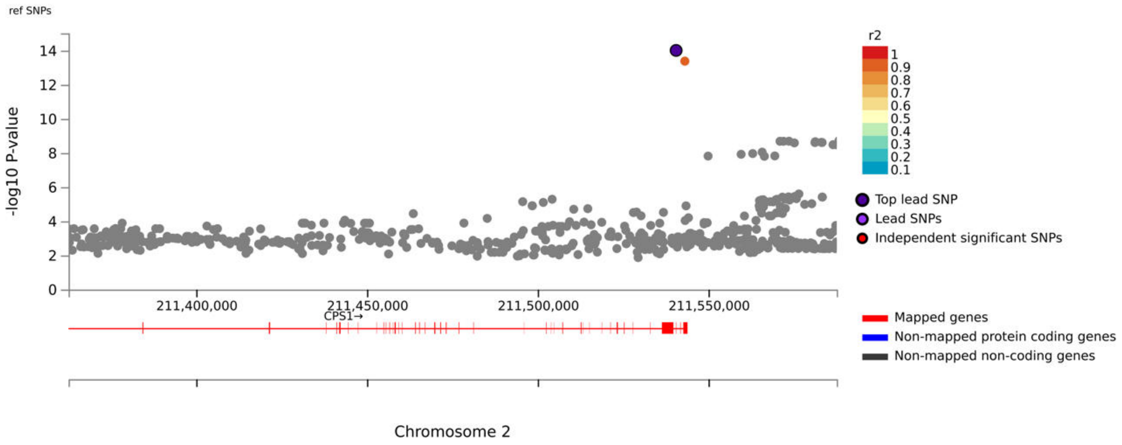

ref SNPs
-log10 P-value
0
2
4
6
8
10
12
14
211,400,000
211,450,000
211,500,000
211,550,000
CPS1→
Chromosome 2
r2
1
0.9
0.8
0.7
0.6
0.5
0.4
0.3
0.2
0.1
Top lead SNP
Lead SNPs
Independent significant SNPs
Mapped genes
Non-mapped protein coding genes
Non-mapped non-coding genes

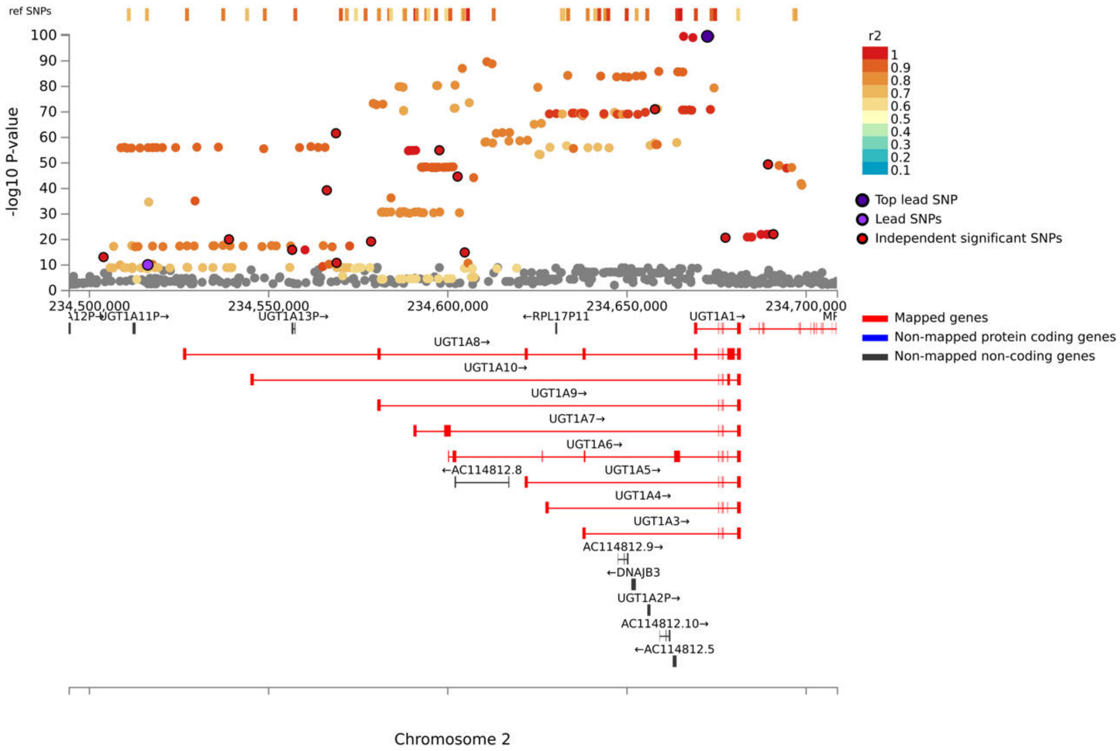
ref SNPs
-log10 P-value
r2
Top lead SNP
Lead SNPs
Independent significant SNPs
Mapped genes
Non-mapped protein coding genes
Non-mapped non-coding genes
234,500,000
234,550,000
234,600,000
234,650,000
234,700,000
UGT1A11P→
UGT1A13P→
←RPL17P11
UGT1A1→
UGT1A8→
UGT1A10→
UGT1A9→
UGT1A7→
UGT1A6→
←AC114812.8
UGT1A5→
UGT1A4→
UGT1A3→
AC114812.9→
←DNAJB3
UGT1A2P→
AC114812.10→
←AC114812.5
Chromosome 2

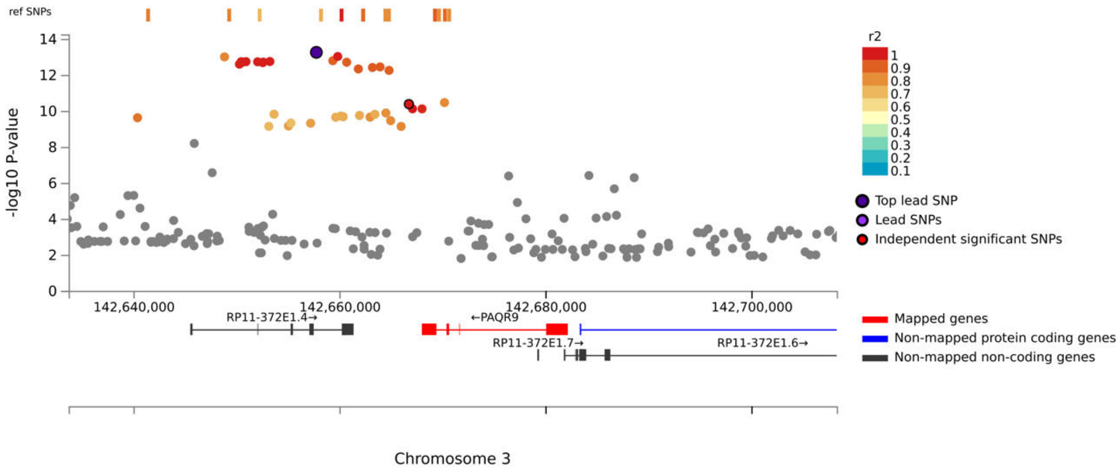
ref SNPs
-log10 P-value
r2
Top lead SNP
Lead SNPs
Independent significant SNPs
Mapped genes
Non-mapped protein coding genes
Non-mapped non-coding genes
142,640,000
142,660,000
142,680,000
142,700,000
RP11-372E1.4→
←PAQR9
RP11-372E1.7→
RP11-372E1.6→
Chromosome 3

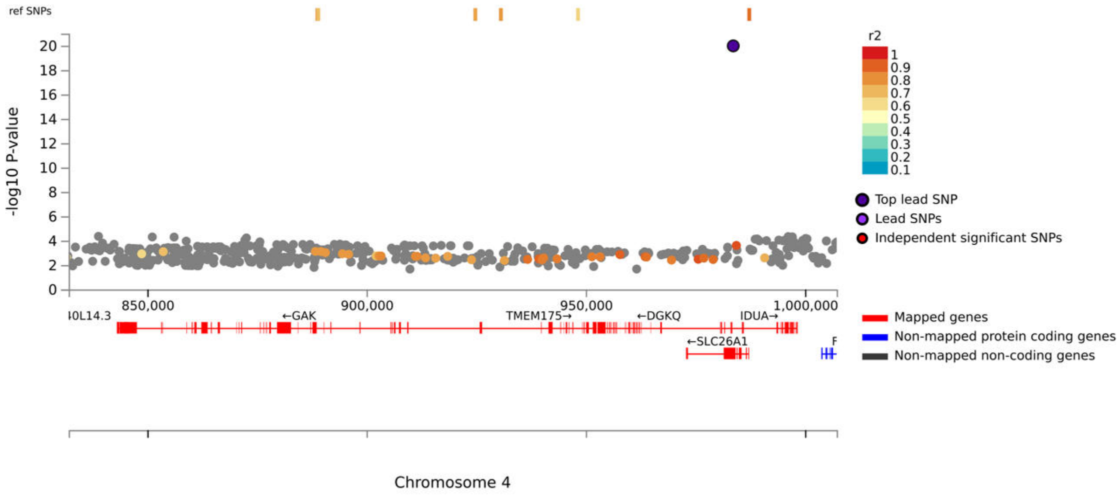
ref SNPs
-log10 P-value
r2
1
0.9
0.8
0.7
0.6
0.5
0.4
0.3
0.2
0.1
Top lead SNP
Lead SNPs
Independent significant SNPs
850,000
900,000
950,000
1,000,000
←GAK
TMEM175→
←DGKQ
IDUA→
←SLC26A1
Mapped genes
Non-mapped protein coding genes
Non-mapped non-coding genes
Chromosome 4

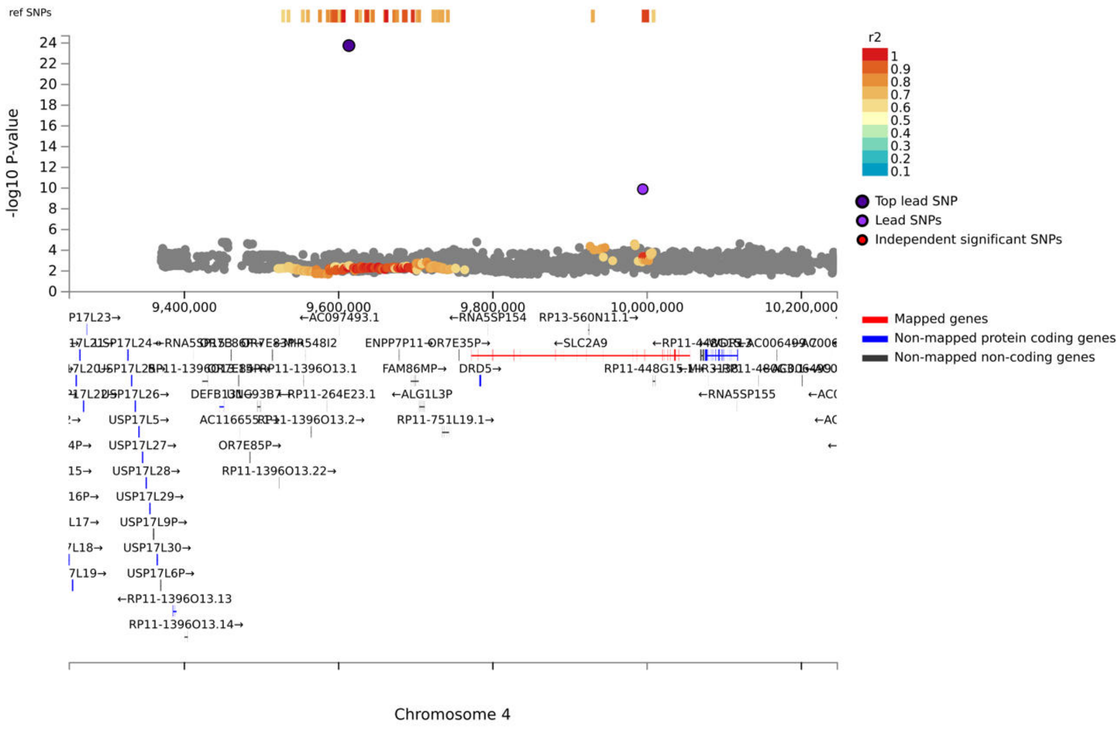
ref SNPs
-log10 P-value
r2
1
0.9
0.8
0.7
0.6
0.5
0.4
0.3
0.2
0.1
Top lead SNP
Lead SNPs
Independent significant SNPs
9,400,000
9,600,000
9,800,000
10,000,000
10,200,000
←AC097493.1
←RNA5SP154
RP13-560N11.1→
ENPP7P11
←OR7E35P→
←SLC2A9
FAM86MP→
DRD5→
←ALG1L3P
RP11-751L19.1→
←RNA5SP155
USP17L5→
USP17L27→
USP17L28→
USP17L29→
USP17L9P→
USP17L30→
USP17L6P→
←RP11-1396O13.13
RP11-1396O13.14→
OR7E85P→
RP11-1396O13.22→
←RP11-264E23.1
RP11-1396O13.2→
Mapped genes
Non-mapped protein coding genes
Non-mapped non-coding genes
Chromosome 4

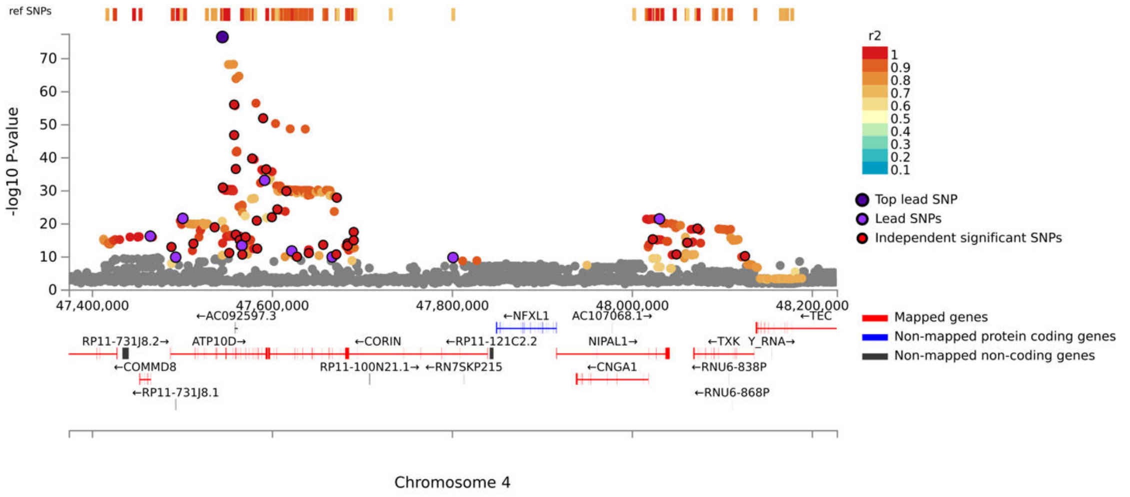
ref SNPs
-log10 P-value
0
10
20
30
40
50
60
70
47,400,000
47,600,000
47,800,000
48,000,000
48,200,000
r2
1
0.9
0.8
0.7
0.6
0.5
0.4
0.3
0.2
0.1
Top lead SNP
Lead SNPs
Independent significant SNPs
Mapped genes
Non-mapped protein coding genes
Non-mapped non-coding genes
←AC092597.3
←NFXL1
AC107068.1→
←TEC
RP11-731J8.2→
ATP10D→
←CORIN
←RP11-121C2.2
NIPAL1→
←TXK
Y_RNA→
←COMMD8
RP11-100N21.1→
←RN7SKP215
←CNGA1
←RNU6-838P
←RP11-731J8.1
←RNU6-868P
Chromosome 4

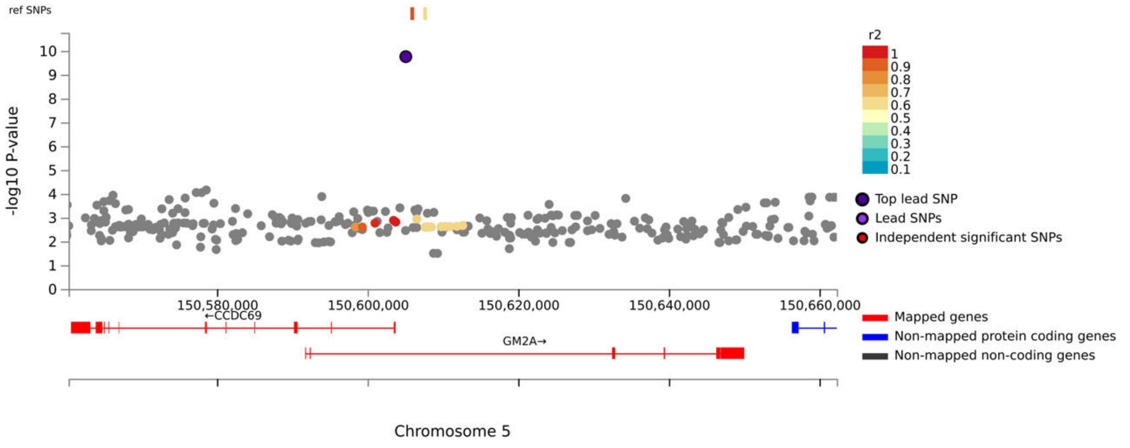
ref SNPs
-log10 P-value
0
1
2
3
4
5
6
7
8
9
10
150,580,000
150,600,000
150,620,000
150,640,000
150,660,000
r2
1
0.9
0.8
0.7
0.6
0.5
0.4
0.3
0.2
0.1
Top lead SNP
Lead SNPs
Independent significant SNPs
Mapped genes
Non-mapped protein coding genes
Non-mapped non-coding genes
←CCDC69
GM2A→
Chromosome 5

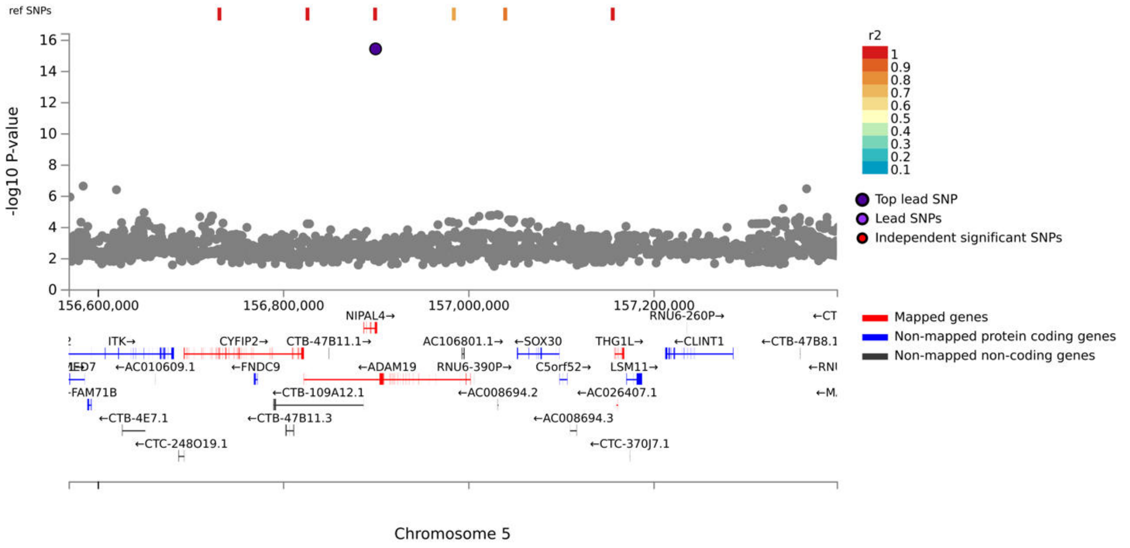
ref SNPs
-log10 P-value
0
2
4
6
8
10
12
14
16
156,600,000
156,800,000
157,000,000
157,200,000
r2
1
0.9
0.8
0.7
0.6
0.5
0.4
0.3
0.2
0.1
Top lead SNP
Lead SNPs
Independent significant SNPs
Mapped genes
Non-mapped protein coding genes
Non-mapped non-coding genes
NIPAL4→
RNU6-260P→
ITK→
CYFIP2→
CTB-47B11.1→
AC106801.1→
←SOX30
THG1L→
←CLINT1
←CTB-47B8.1
←AC010609.1
←FNDC9
←ADAM19
RNU6-390P→
C5orf52→
LSM11→
-FAM71B
←CTB-109A12.1
←AC008694.2
←AC026407.1
←CTB-4E7.1
←CTB-47B11.3
←AC008694.3
←CTC-248O19.1
←CTC-370J7.1
Chromosome 5

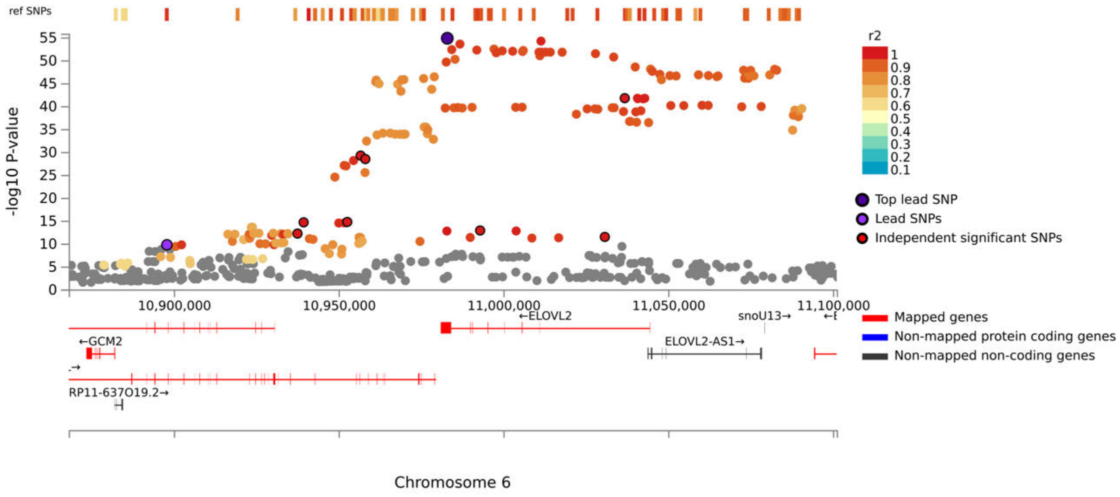
ref SNPs
-log10 P-value
55
50
45
40
35
30
25
20
15
10
5
0
10,900,000
10,950,000
11,000,000
11,050,000
11,100,000
r2
1
0.9
0.8
0.7
0.6
0.5
0.4
0.3
0.2
0.1
Top lead SNP
Lead SNPs
Independent significant SNPs
←ELOVL2
snoU13→
←GCM2
ELOVL2-AS1→
RP11-637O19.2→
Mapped genes
Non-mapped protein coding genes
Non-mapped non-coding genes
Chromosome 6

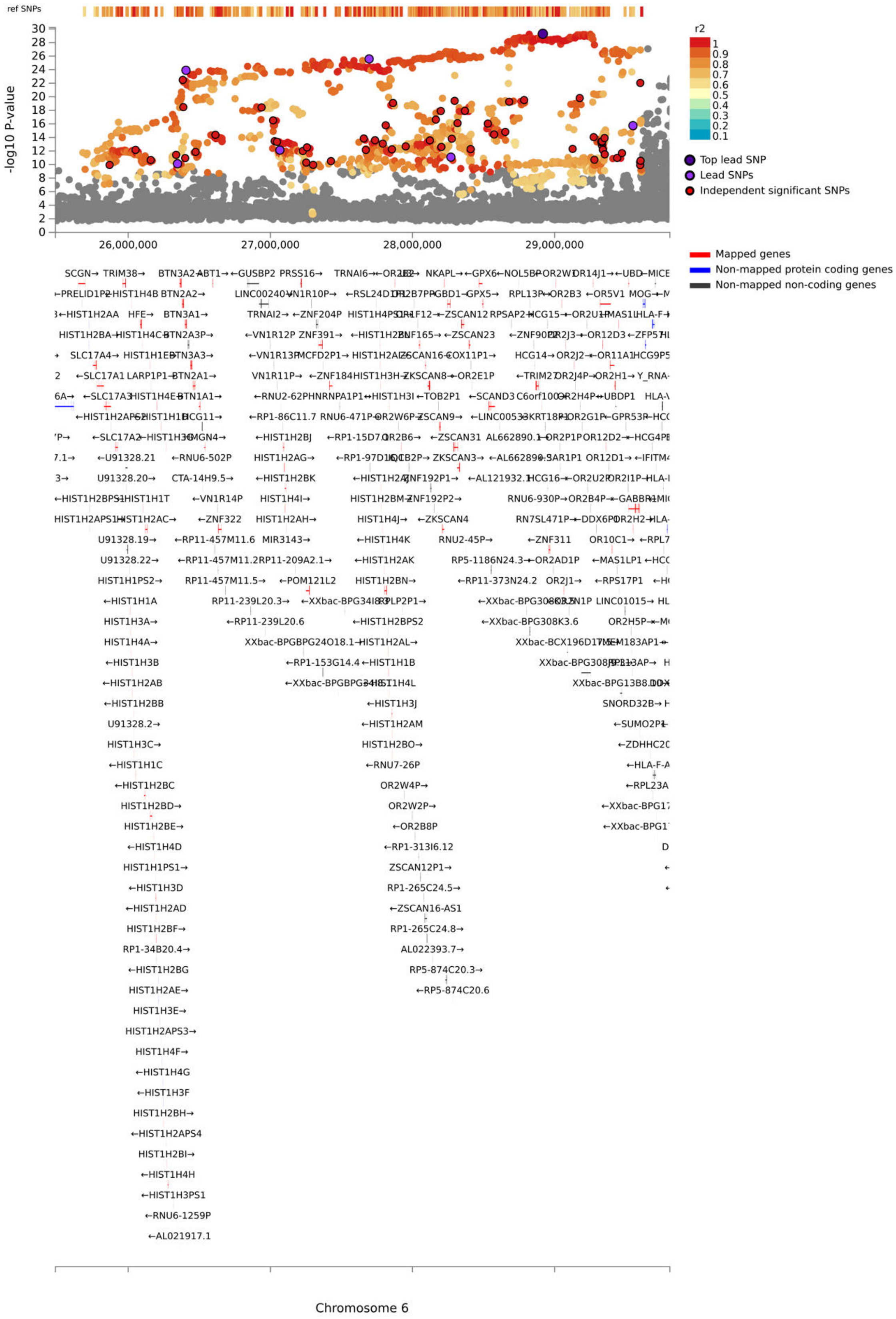
ref SNPs
-log10 P-value
r2
1
0.9
0.8
0.7
0.6
0.5
0.4
0.3
0.2
0.1
Top lead SNP
Lead SNPs
Independent significant SNPs
26,000,000
27,000,000
28,000,000
29,000,000
Mapped genes
Non-mapped protein coding genes
Non-mapped non-coding genes
Chromosome 6

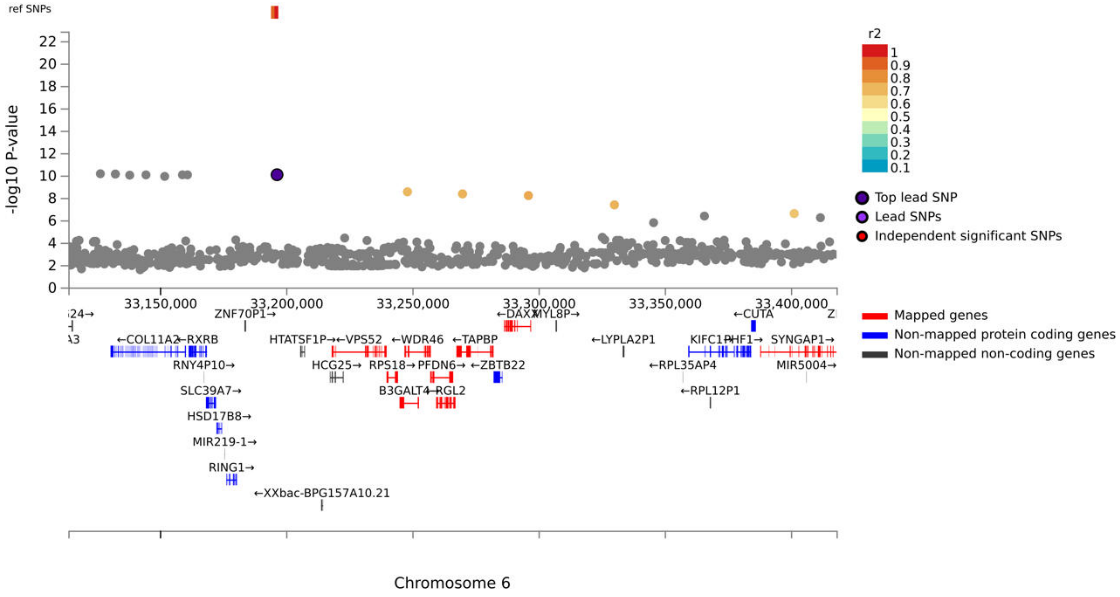
ref SNPs
-log10 P-value
33,150,000
33,200,000
33,250,000
33,300,000
33,350,000
33,400,000
r2
Top lead SNP
Lead SNPs
Independent significant SNPs
Mapped genes
Non-mapped protein coding genes
Non-mapped non-coding genes
ZNF70P1→
←DAXX
MYL8P→
←CUTA
←COL11A2
←RXRB
HTATSF1P→
←VPS52
←WDR46
←TAPBP
←LYPLA2P1
KIFC1
PHF1→
SYNGAP1→
RNY4P10→
HCG25→
RPS18→
PFDN6→
←ZBTB22
←RPL35AP4
MIR5004→
SLC39A7→
B3GALT4
←RGL2
←RPL12P1
HSD17B8→
MIR219-1→
RING1→
←XXbac-BPG157A10.21
Chromosome 6

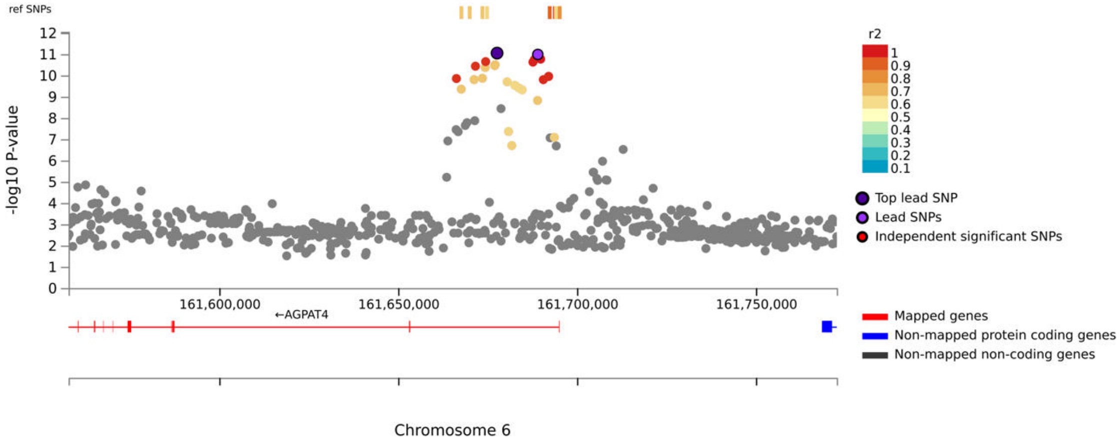
ref SNPs
-log10 P-value
161,600,000
161,650,000
161,700,000
161,750,000
←AGPAT4
r2
Top lead SNP
Lead SNPs
Independent significant SNPs
Mapped genes
Non-mapped protein coding genes
Non-mapped non-coding genes
Chromosome 6

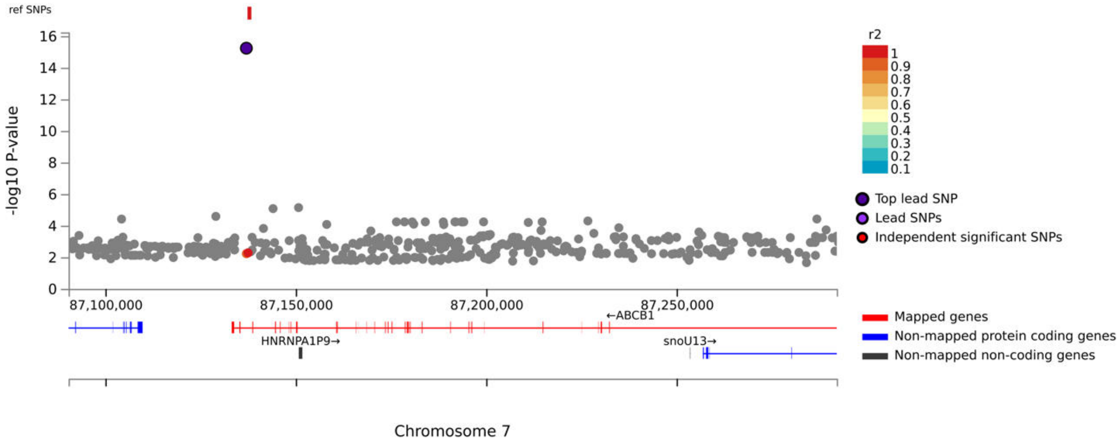
ref SNPs
-log10 P-value
87,100,000
87,150,000
87,200,000
87,250,000
←ABCB1
HNRNPA1P9→
snoU13→
r2
Top lead SNP
Lead SNPs
Independent significant SNPs
Mapped genes
Non-mapped protein coding genes
Non-mapped non-coding genes
Chromosome 7

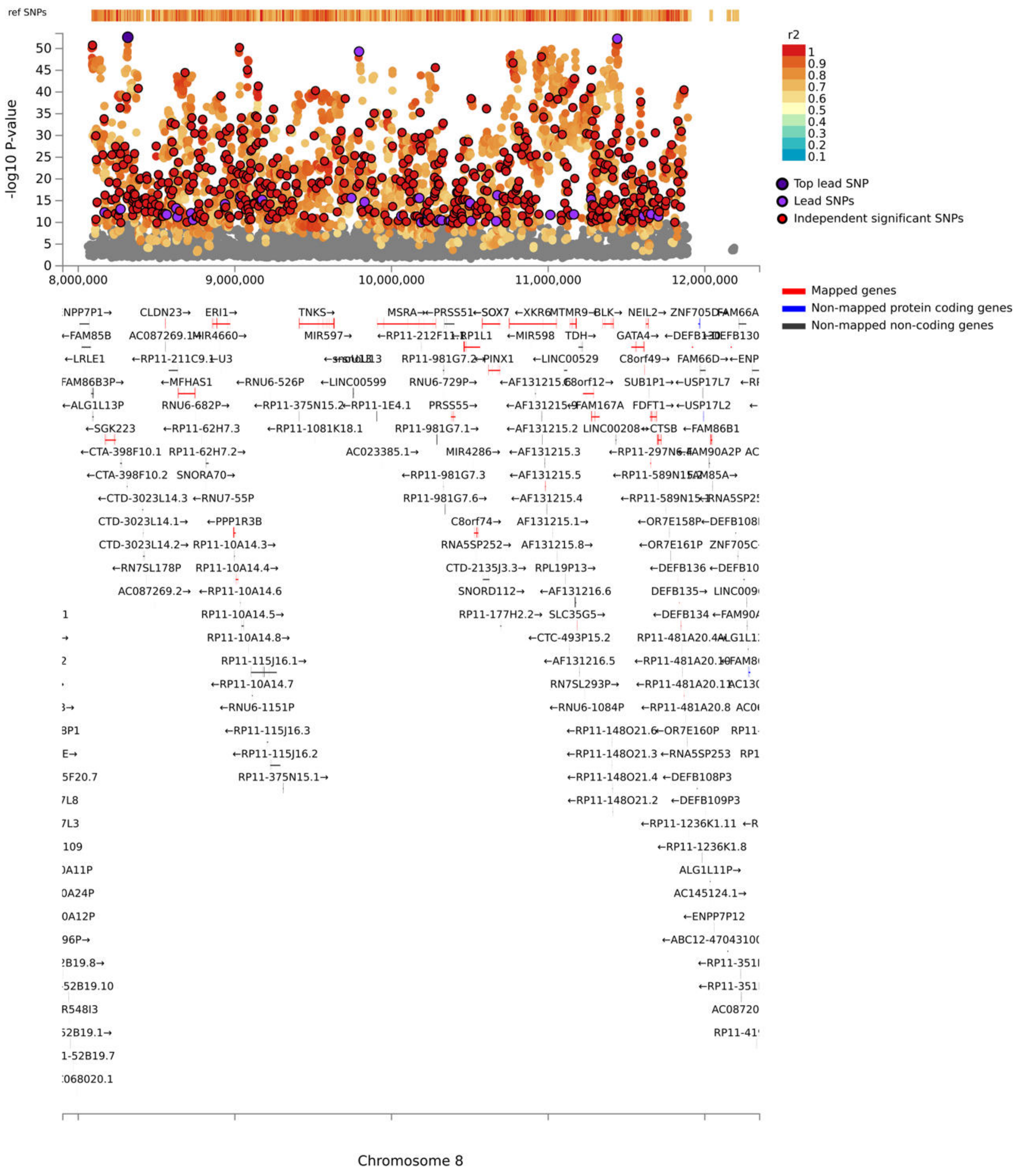
ref SNPs
-log10 P-value
0
5
10
15
20
25
30
35
40
45
50
8,000,000
9,000,000
10,000,000
11,000,000
12,000,000
r2
1
0.9
0.8
0.7
0.6
0.5
0.4
0.3
0.2
0.1
Top lead SNP
Lead SNPs
Independent significant SNPs
Mapped genes
Non-mapped protein coding genes
Non-mapped non-coding genes
Chromosome 8

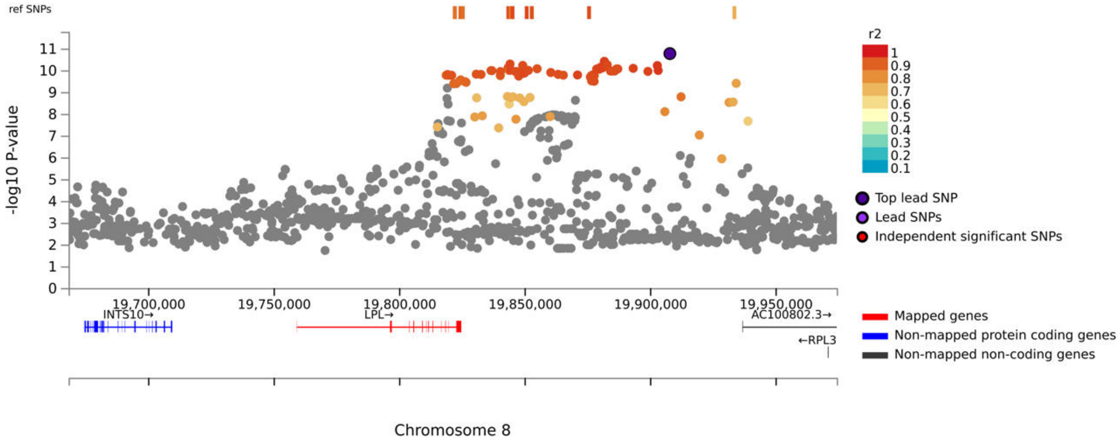
ref SNPs
-log10 P-value
r2
Top lead SNP
Lead SNPs
Independent significant SNPs
INTS10→
LPL→
AC100802.3→
←RPL3
Mapped genes
Non-mapped protein coding genes
Non-mapped non-coding genes
19,700,000
19,750,000
19,800,000
19,850,000
19,900,000
19,950,000
Chromosome 8

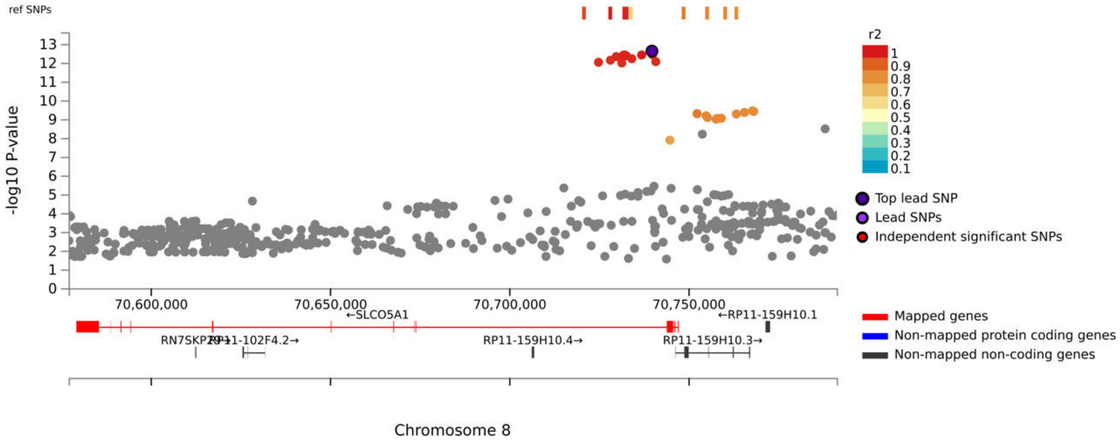
ref SNPs
-log10 P-value
r2
Top lead SNP
Lead SNPs
Independent significant SNPs
←SLCO5A1
←RP11-159H10.1
RP11-159H10.4→
RP11-159H10.3→
Mapped genes
Non-mapped protein coding genes
Non-mapped non-coding genes
70,600,000
70,650,000
70,700,000
70,750,000
Chromosome 8

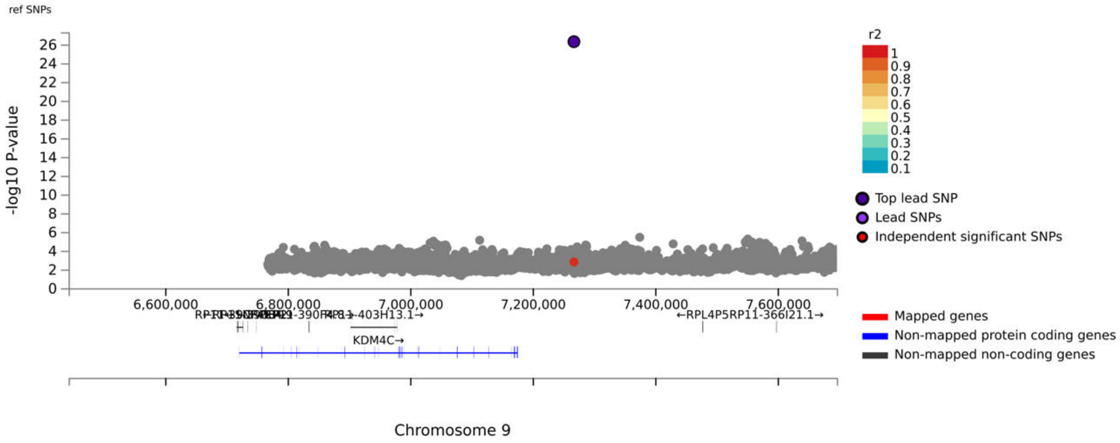
ref SNPs
-log10 P-value
r2
Top lead SNP
Lead SNPs
Independent significant SNPs
KDM4C→
Mapped genes
Non-mapped protein coding genes
Non-mapped non-coding genes
6,600,000
6,800,000
7,000,000
7,200,000
7,400,000
7,600,000
Chromosome 9

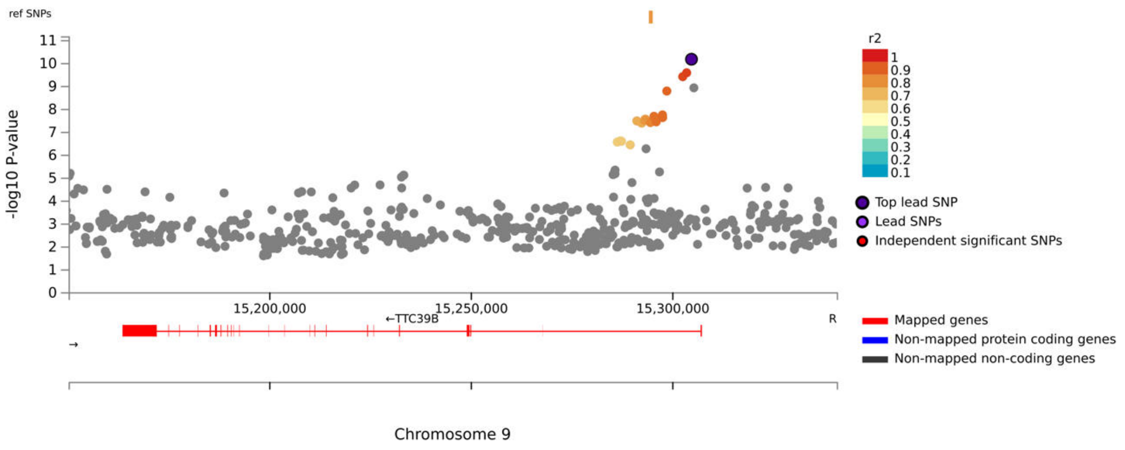
ref SNPs
-log10 P-value
r2
1
0.9
0.8
0.7
0.6
0.5
0.4
0.3
0.2
0.1
Top lead SNP
Lead SNPs
Independent significant SNPs
15,200,000
15,250,000
15,300,000
←TTC39B
Mapped genes
Non-mapped protein coding genes
Non-mapped non-coding genes
Chromosome 9

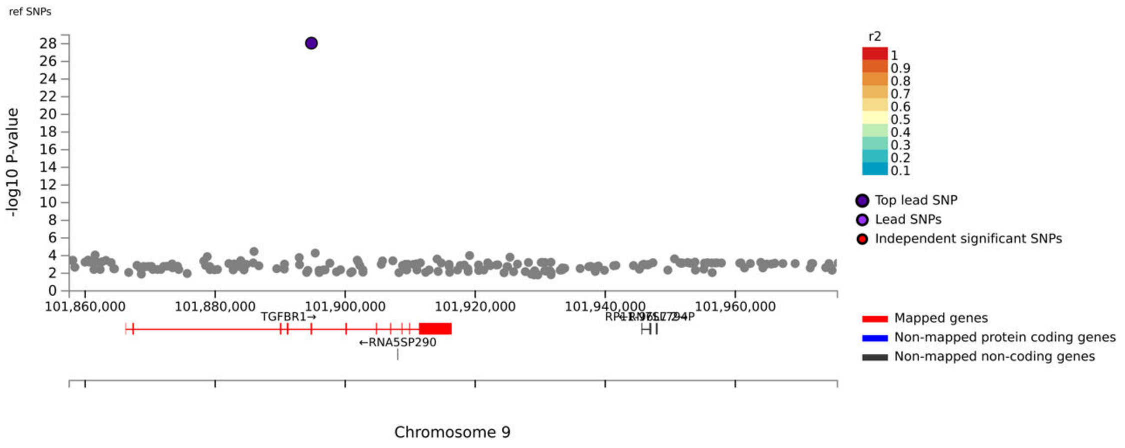
ref SNPs
-log10 P-value
r2
Top lead SNP
Lead SNPs
Independent significant SNPs
101,860,000
101,880,000
101,900,000
101,920,000
101,940,000
101,960,000
TGFBR1→
←RNA5SP290
Mapped genes
Non-mapped protein coding genes
Non-mapped non-coding genes
Chromosome 9

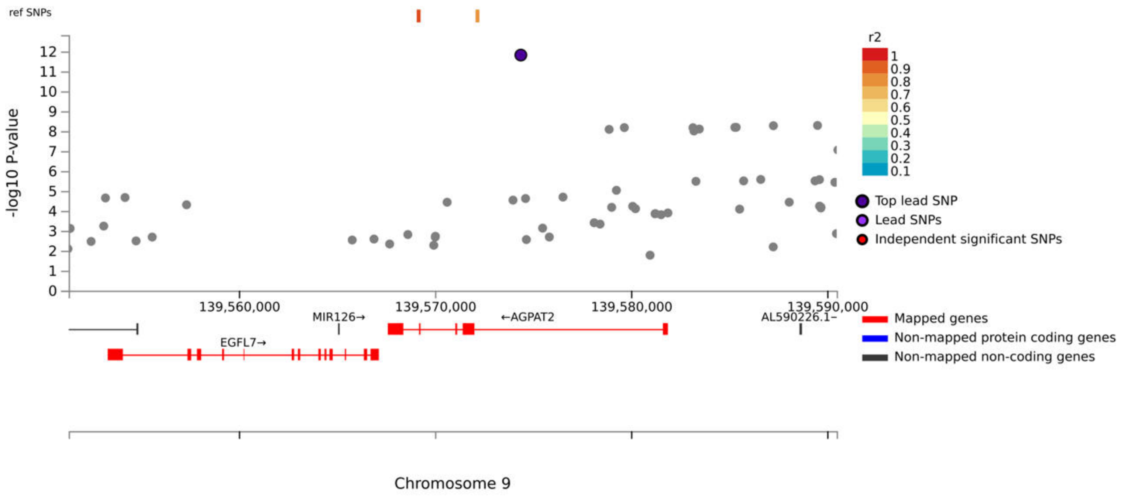
ref SNPs
-log10 P-value
r2
Top lead SNP
Lead SNPs
Independent significant SNPs
139,560,000
139,570,000
139,580,000
139,590,000
MIR126→
←AGPAT2
EGFL7→
AL590226.1←
Mapped genes
Non-mapped protein coding genes
Non-mapped non-coding genes
Chromosome 9

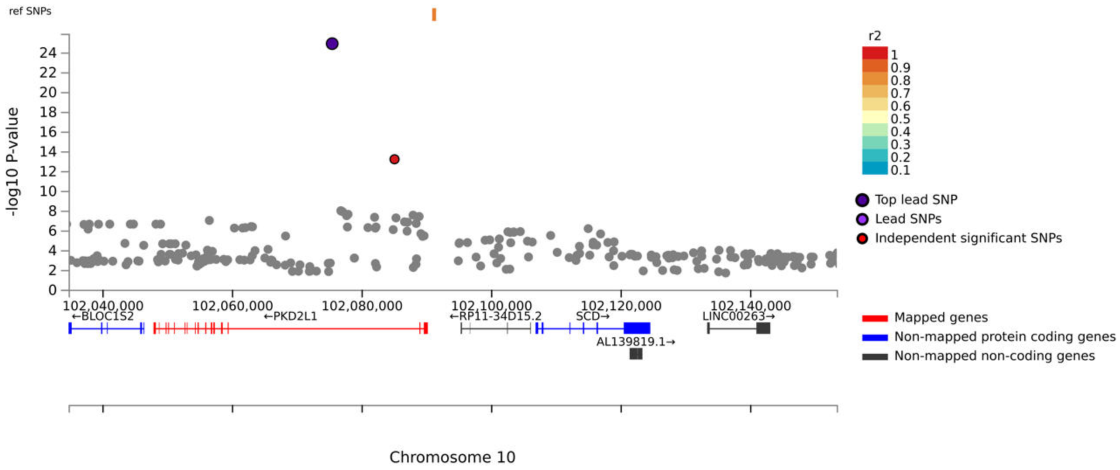
ref SNPs
-log10 P-value
r2
1
0.9
0.8
0.7
0.6
0.5
0.4
0.3
0.2
0.1
Top lead SNP
Lead SNPs
Independent significant SNPs
102,040,000
102,060,000
102,080,000
102,100,000
102,120,000
102,140,000
←BLOC1S2
←PKD2L1
←RP11-34D15.2
SCD→
LINC00263→
AL139819.1→
Mapped genes
Non-mapped protein coding genes
Non-mapped non-coding genes
Chromosome 10

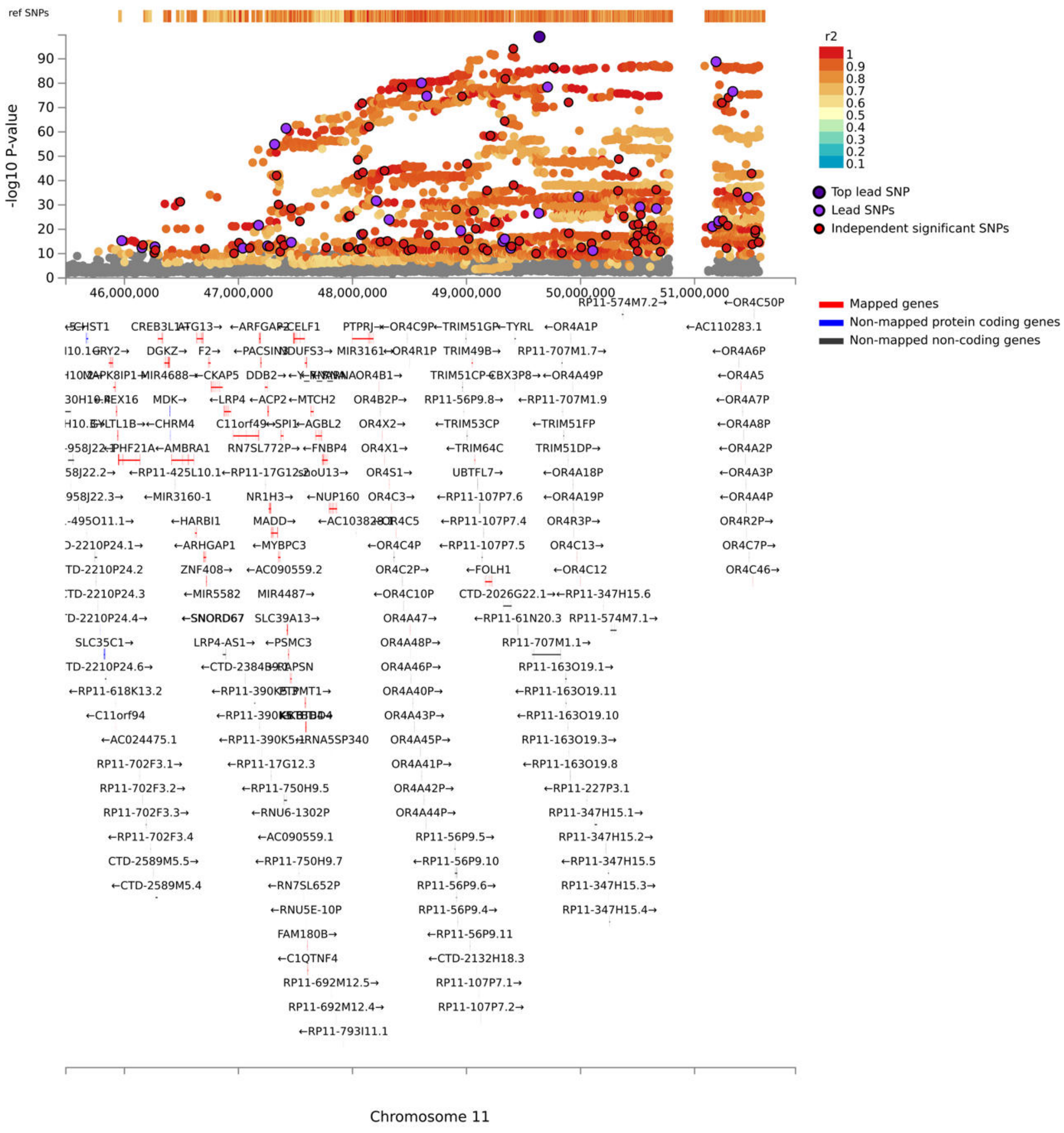
ref SNPs
-log10 P-value
0
10
20
30
40
50
60
70
80
90
46,000,000
47,000,000
48,000,000
49,000,000
50,000,000
51,000,000
r2
1
0.9
0.8
0.7
0.6
0.5
0.4
0.3
0.2
0.1
Top lead SNP
Lead SNPs
Independent significant SNPs
Mapped genes
Non-mapped protein coding genes
Non-mapped non-coding genes
RP11-574M7.2→
←OR4C50P
←AC110283.1
←OR4A6P
←OR4A5
←OR4A7P
←OR4A8P
←OR4A2P
←OR4A3P
←OR4A4P
OR4R2P→
OR4C7P→
OR4C46→
←OR4A1P
RP11-707M1.7→
←OR4A49P
←RP11-707M1.9
←TRIM51FP
TRIM51DP→
←OR4A18P
←OR4A19P
OR4R3P→
OR4C13→
←OR4C12
←RP11-347H15.6
RP11-574M7.1→
RP11-707M1.1→
RP11-163O19.1→
←RP11-163O19.11
←RP11-163O19.10
RP11-163O19.3→
←RP11-163O19.8
←RP11-227P3.1
RP11-347H15.1→
RP11-347H15.2→
←RP11-347H15.5
RP11-347H15.3→
RP11-347H15.4→
←TRIM51GP
←TYRL
TRIM49B→
TRIM51CP
CBX3P8→
RP11-56P9.8→
←TRIM53CP
←TRIM64C
UBTFL7→
←RP11-107P7.6
←RP11-107P7.4
←RP11-107P7.5
←FOLH1
CTD-2026G22.1→
←RP11-61N20.3
RP11-56P9.5→
←RP11-56P9.10
RP11-56P9.6→
RP11-56P9.4→
←RP11-56P9.11
←CTD-2132H18.3
RP11-107P7.1→
RP11-107P7.2→
←OR4C9P
←OR4R1P
OR4B1→
OR4B2P→
OR4X2→
OR4X1→
OR4S1→
OR4C3→
OR4C5
←OR4C4P
OR4C2P→
←OR4C10P
OR4A47→
OR4A48P→
OR4A46P→
OR4A40P→
OR4A43P→
OR4A45P→
OR4A41P→
OR4A42P→
OR4A44P→
PTPRJ
CELF1
NDUFS3→
MIR3161
←MTCH2
←AGBL2
←FNBP4
NUP160
←ARFGAP2
←PACSIN3
DDB2→
←ACP2
←SPI1
C11orf49
RN7SL772P→
NR1H3→
MADD→
←MYBPC3
←AC090559.2
MIR4487→
SLC39A13→
←PSMC3
←RP11-17G12.3
←RP11-750H9.5
←RNU6-1302P
←AC090559.1
←RP11-750H9.7
←RN7SL652P
←RNU5E-10P
FAM180B→
←C1QTNF4
RP11-692M12.5→
RP11-692M12.4→
←RP11-793I11.1
CREB3L1
ATG13→
DGKZ→
F2→
MIR4688
←CKAP5
MDK→
←LRP4
←CHRM4
←AMBRA1
←HARBI1
←ARHGAP1
ZNF408→
←MIR5582
←SNORD67
LRP4-AS1→
←RP11-425L10.1
←MIR3160-1
CHST1
CRY2→
MAPK8IP1→
PEX16
PHF21A
SLC35C1→
←RP11-618K13.2
←C11orf94
←AC024475.1
RP11-702F3.1→
RP11-702F3.2→
RP11-702F3.3→
←RP11-702F3.4
CTD-2589M5.5→
←CTD-2589M5.4
←RNA5SP340
Chromosome 11

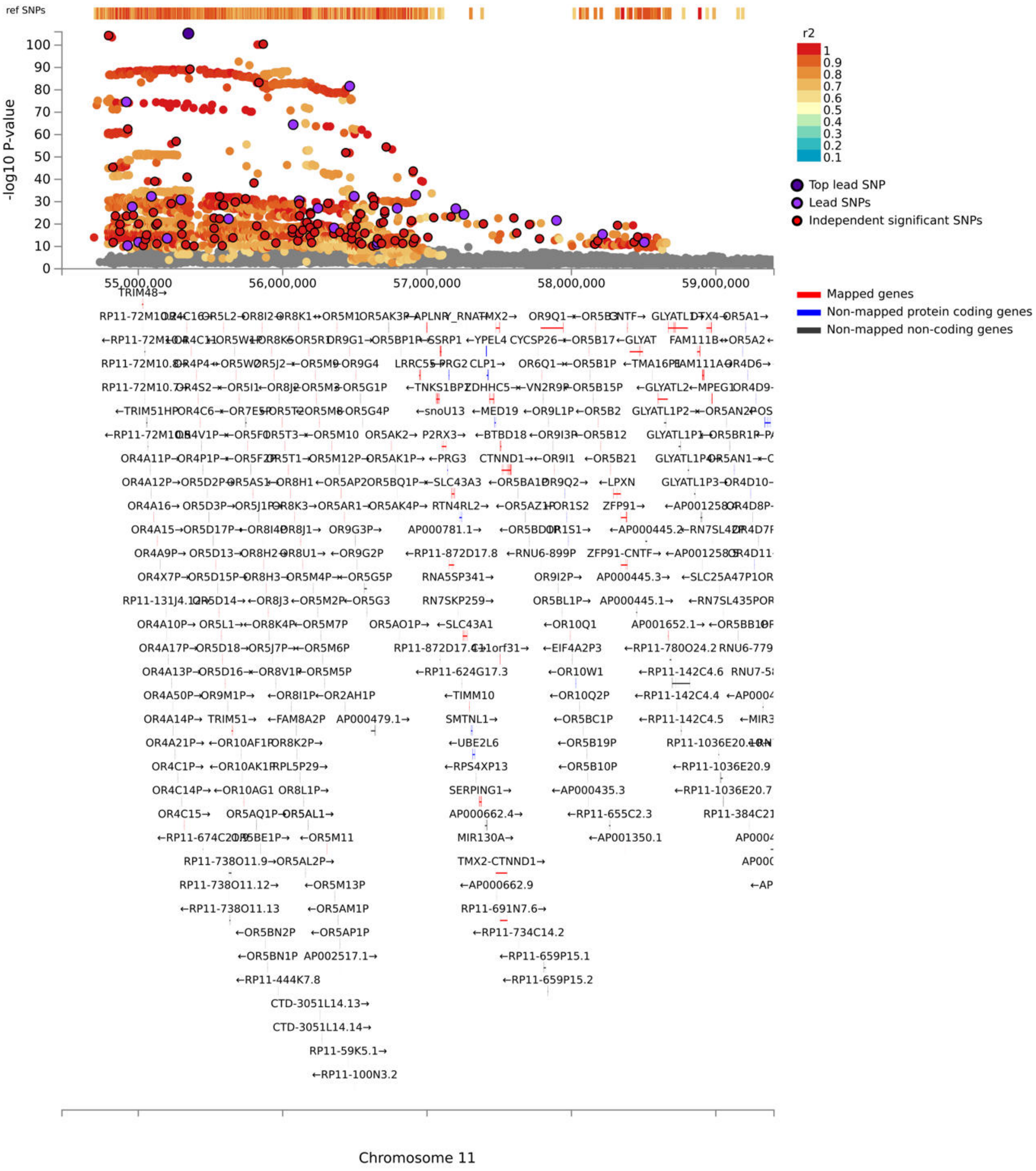

ref SNPs
-log10 P-value
0
10
20
30
40
50
60
70
80
90
100
55,000,000
56,000,000
57,000,000
58,000,000
59,000,000
r2
1
0.9
0.8
0.7
0.6
0.5
0.4
0.3
0.2
0.1
Top lead SNP
Lead SNPs
Independent significant SNPs
Mapped genes
Non-mapped protein coding genes
Non-mapped non-coding genes
Chromosome 11

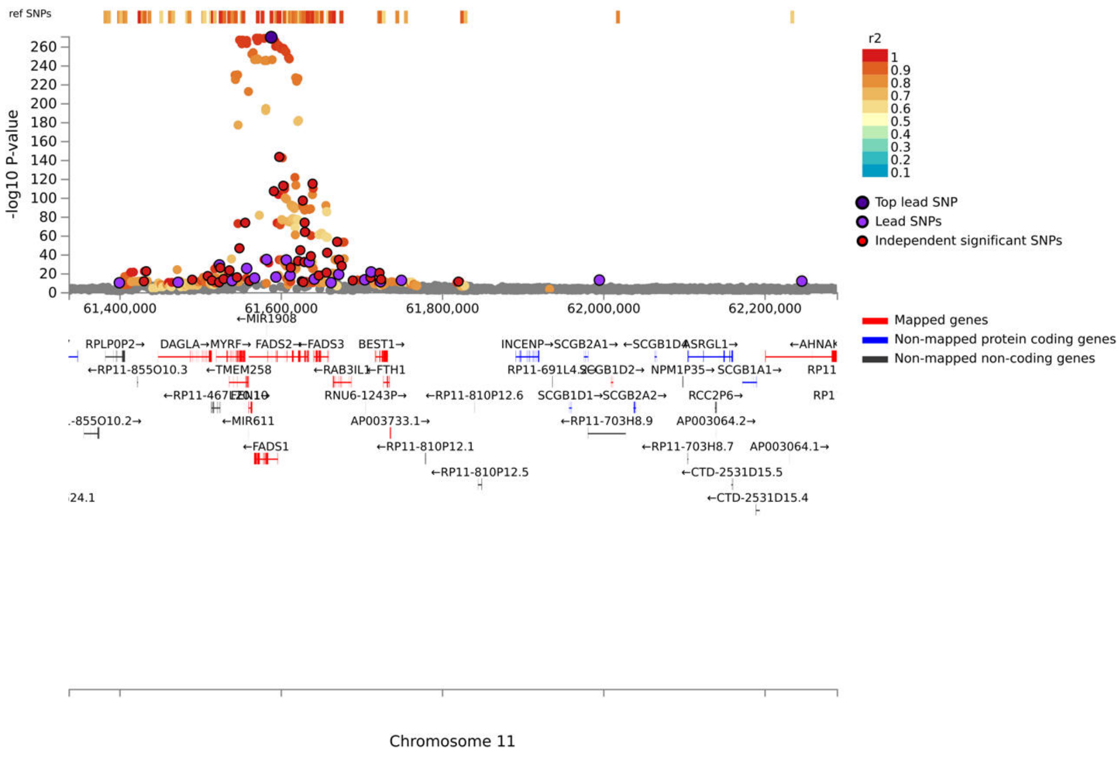
ref SNPs
-log10 P-value
0
20
40
60
80
100
120
140
160
180
200
220
240
260
61,400,000
61,600,000
61,800,000
62,000,000
62,200,000
←MIR1908
RPLP0P2→
DAGLA→
MYRF→
FADS2→
←FADS3
BEST1→
INCENP→
SCGB2A1→
←SCGB1D4
ASRGL1→
←AHNAK
←RP11-855O10.3
←TMEM258
←RAB3IL1
←FTH1
RP11-691L4.2
SCGB1D2→
NPM1P35→
SCGB1A1→
RP11
←RP11-467L20.10
FEN1→
RNU6-1243P→
←RP11-810P12.6
SCGB1D1→
SCGB2A2→
RCC2P6→
RP1
-855O10.2→
←MIR611
AP003733.1→
←RP11-703H8.9
AP003064.2→
←FADS1
←RP11-810P12.1
←RP11-703H8.7
AP003064.1→
←RP11-810P12.5
←CTD-2531D15.5
24.1
←CTD-2531D15.4
r2
1
0.9
0.8
0.7
0.6
0.5
0.4
0.3
0.2
0.1
Top lead SNP
Lead SNPs
Independent significant SNPs
Mapped genes
Non-mapped protein coding genes
Non-mapped non-coding genes
Chromosome 11

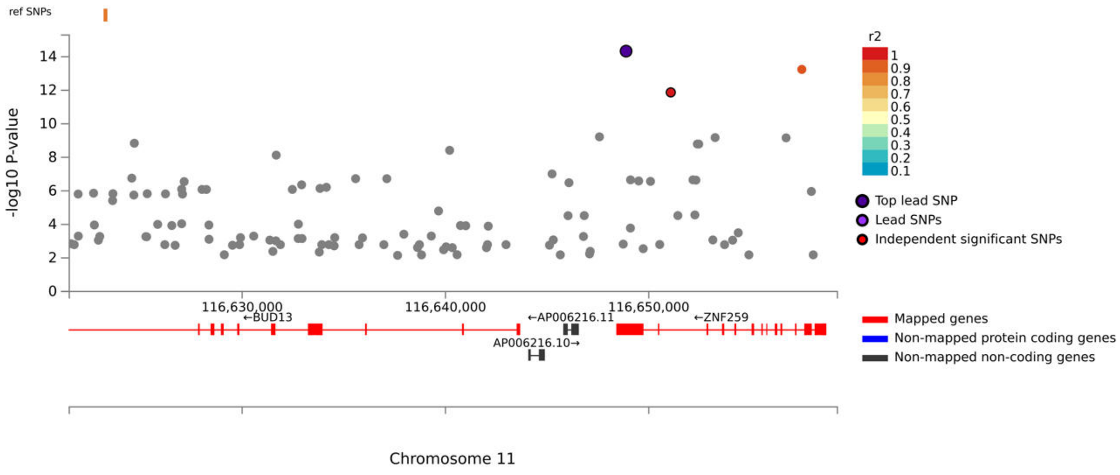
ref SNPs
-log10 P-value
0
2
4
6
8
10
12
14
116,630,000
116,640,000
116,650,000
←BUD13
←AP006216.11
←ZNF259
AP006216.10→
r2
1
0.9
0.8
0.7
0.6
0.5
0.4
0.3
0.2
0.1
Top lead SNP
Lead SNPs
Independent significant SNPs
Mapped genes
Non-mapped protein coding genes
Non-mapped non-coding genes
Chromosome 11

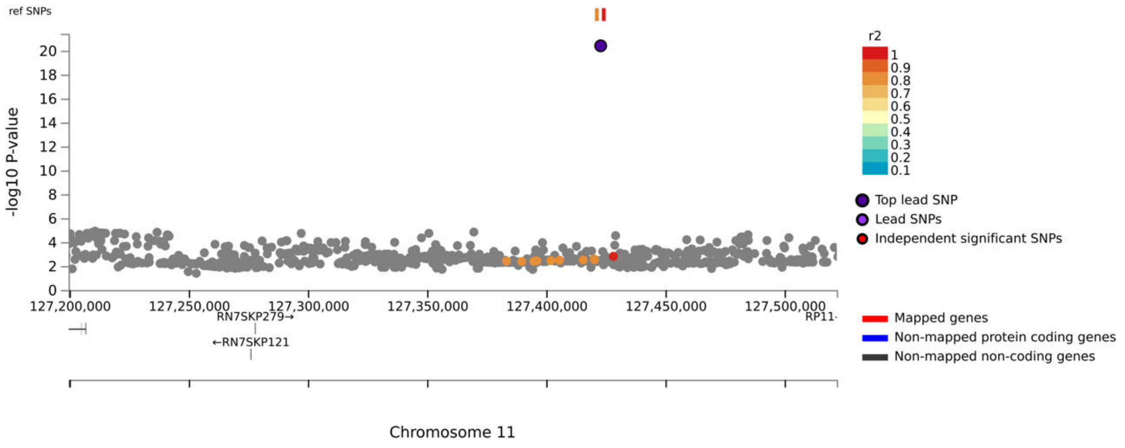
ref SNPs
-log10 P-value
RN7SKP279→
←RN7SKP121
RP11-
r2
Top lead SNP
Lead SNPs
Independent significant SNPs
Mapped genes
Non-mapped protein coding genes
Non-mapped non-coding genes
Chromosome 11

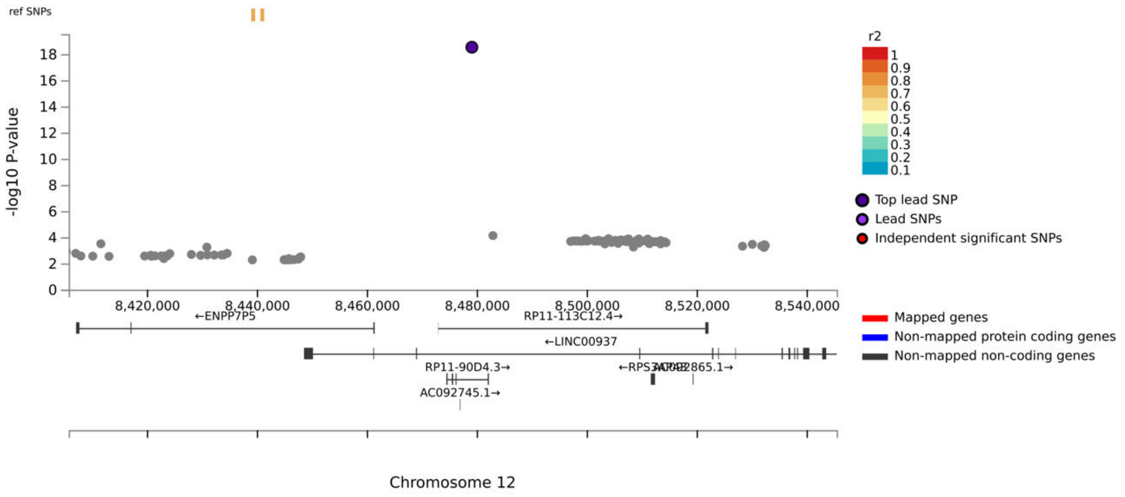
ref SNPs
-log10 P-value
←ENPP7P5
RP11-113C12.4→
←LINC00937
RP11-90D4.3→
AC092745.1→
r2
Top lead SNP
Lead SNPs
Independent significant SNPs
Mapped genes
Non-mapped protein coding genes
Non-mapped non-coding genes
Chromosome 12

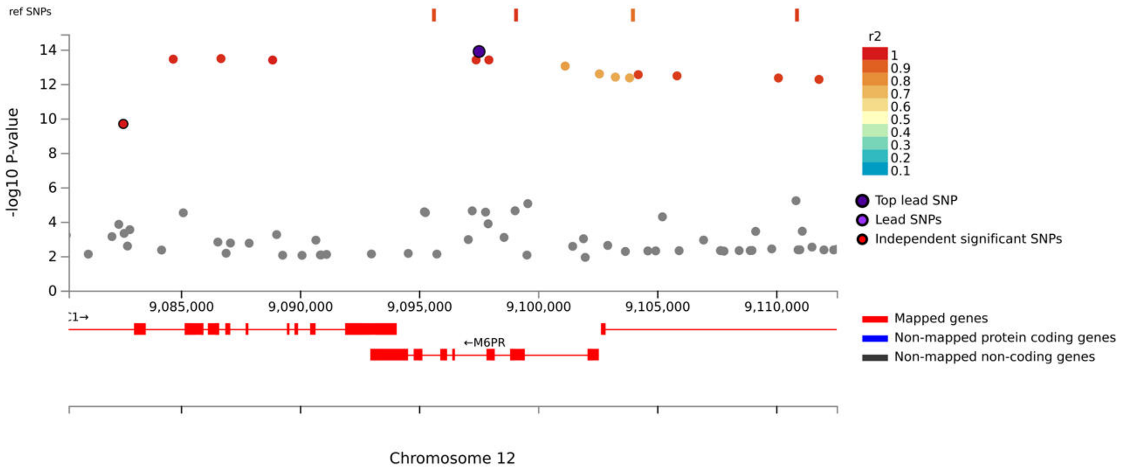
ref SNPs
-log10 P-value
←M6PR
r2
Top lead SNP
Lead SNPs
Independent significant SNPs
Mapped genes
Non-mapped protein coding genes
Non-mapped non-coding genes
Chromosome 12

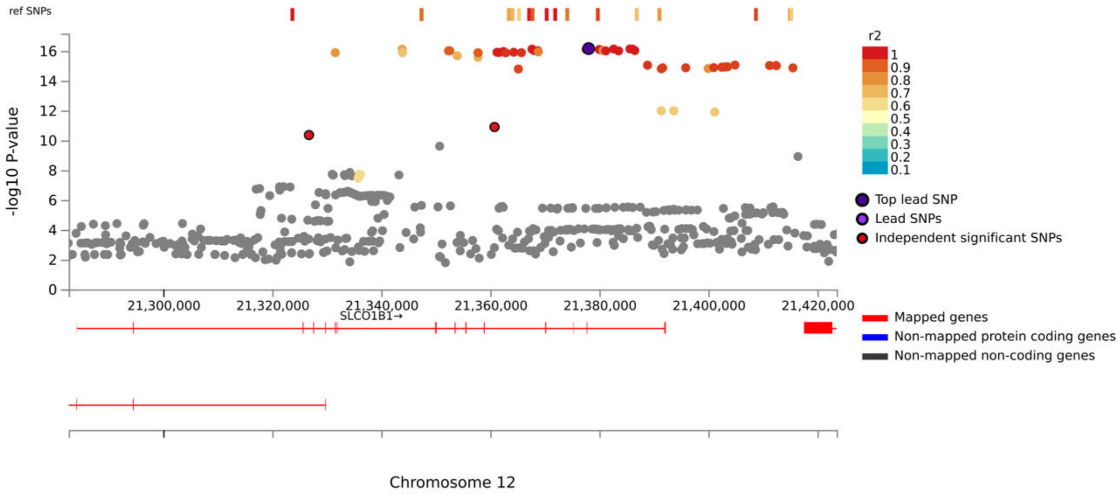
ref SNPs
-log10 P-value
r2
Top lead SNP
Lead SNPs
Independent significant SNPs
21,300,000
21,320,000
21,340,000
21,360,000
21,380,000
21,400,000
21,420,000
SLCO1B1→
Mapped genes
Non-mapped protein coding genes
Non-mapped non-coding genes
Chromosome 12

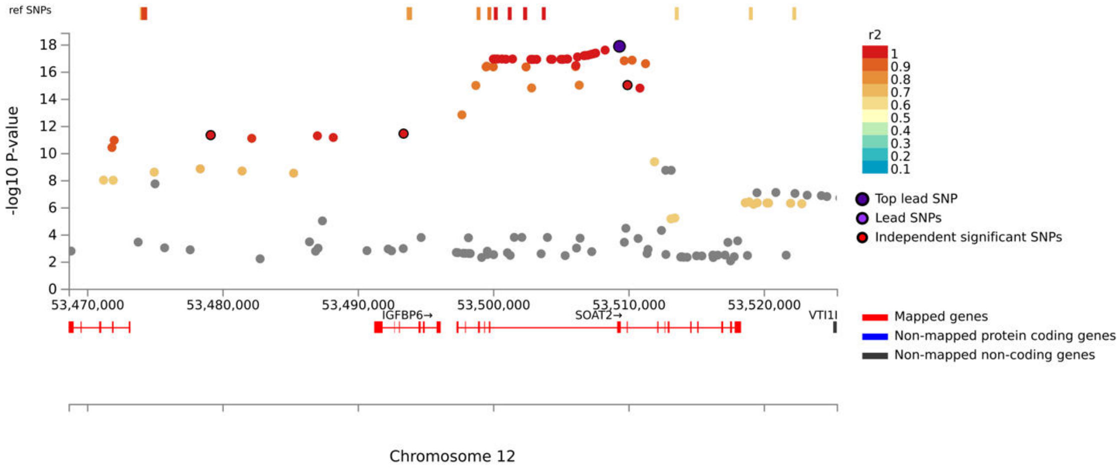
ref SNPs
-log10 P-value
r2
Top lead SNP
Lead SNPs
Independent significant SNPs
53,470,000
53,480,000
53,490,000
53,500,000
53,510,000
53,520,000
IGFBP6→
SOAT2→
Mapped genes
Non-mapped protein coding genes
Non-mapped non-coding genes
Chromosome 12

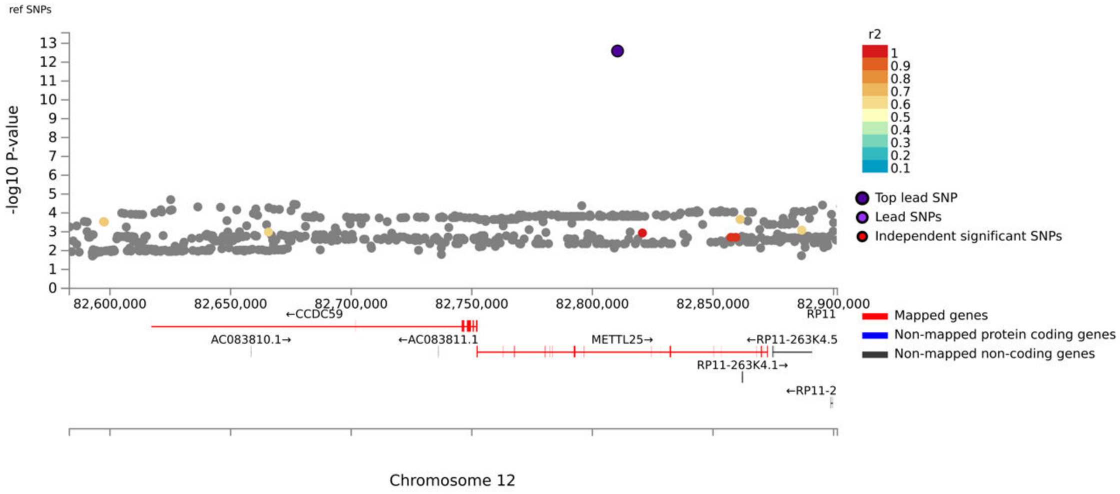
ref SNPs
-log10 P-value
r2
Top lead SNP
Lead SNPs
Independent significant SNPs
82,600,000
82,650,000
82,700,000
82,750,000
82,800,000
82,850,000
82,900,000
←CCDC59
AC083810.1→
←AC083811.1
METTL25→
←RP11-263K4.5
RP11-263K4.1→
←RP11-2
Mapped genes
Non-mapped protein coding genes
Non-mapped non-coding genes
Chromosome 12

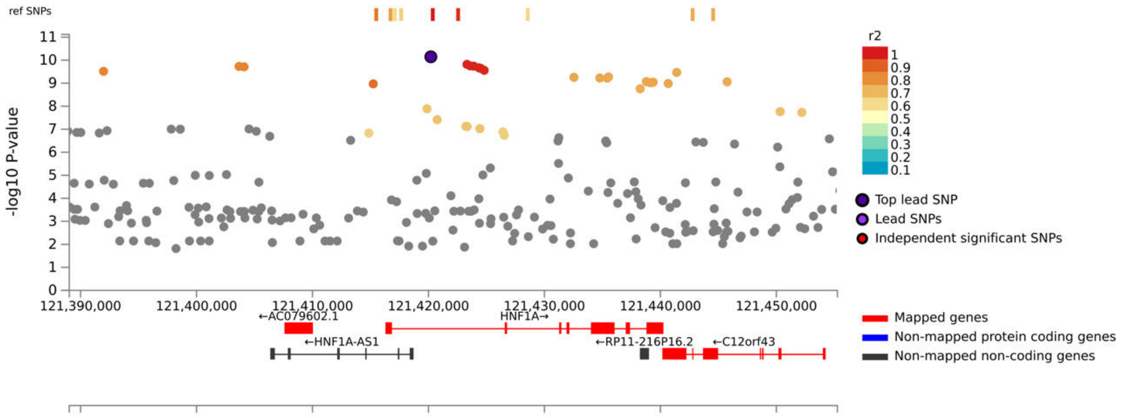

Chromosome 12

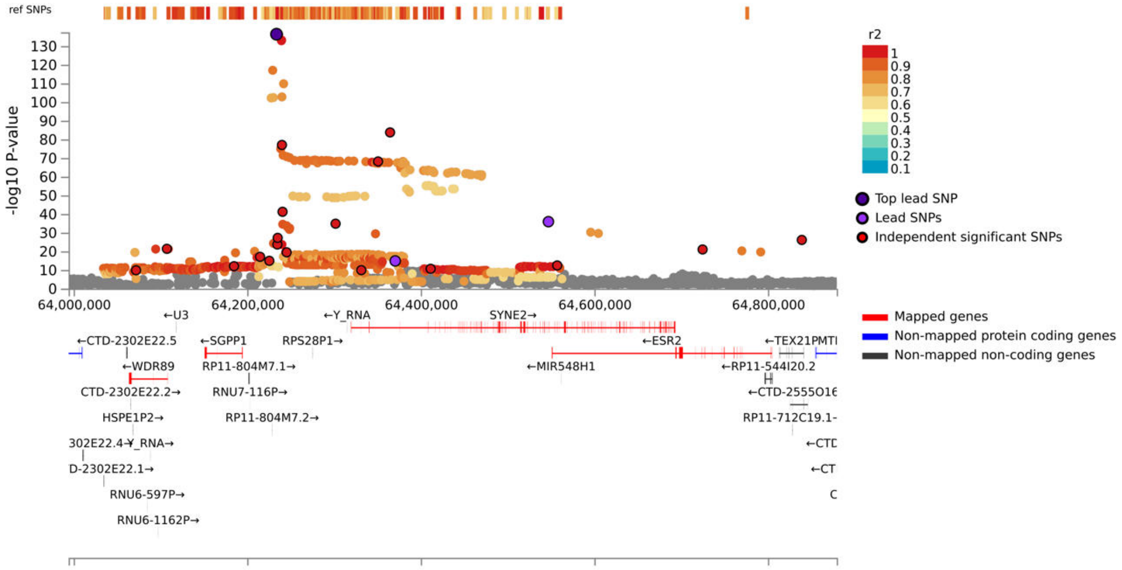

Chromosome 14

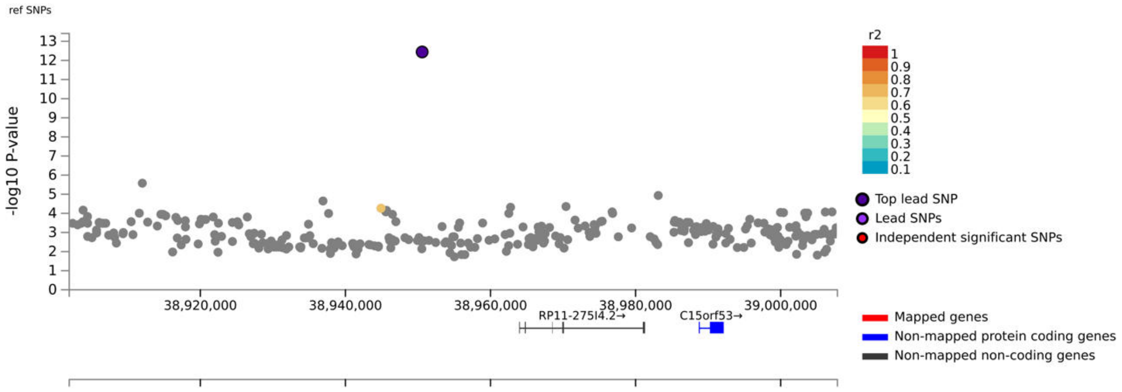

Chromosome 15

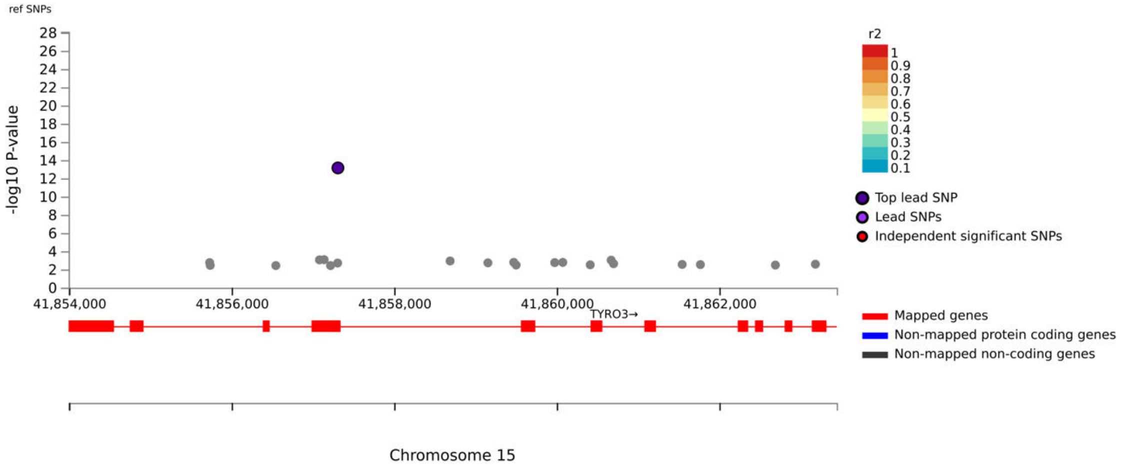

ref SNPs
-log10 P-value
41,854,000
41,856,000
41,858,000
41,860,000
41,862,000
TYRO3→
r2
Top lead SNP
Lead SNPs
Independent significant SNPs
Mapped genes
Non-mapped protein coding genes
Non-mapped non-coding genes
Chromosome 15

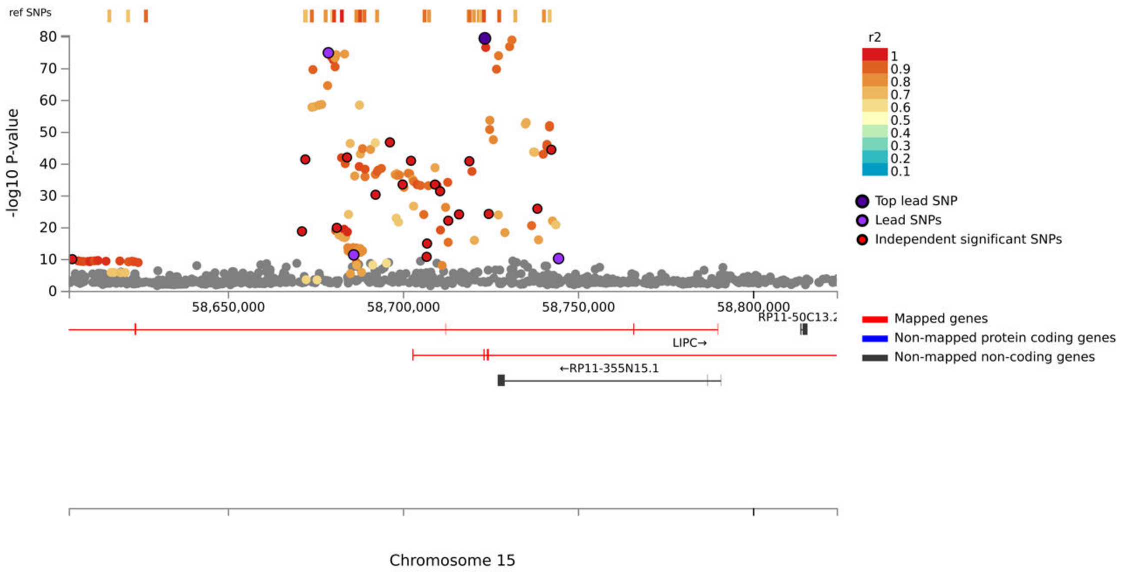

ref SNPs
-log10 P-value
58,650,000
58,700,000
58,750,000
58,800,000
RP11-50C13.2
LIPC→
←RP11-355N15.1
r2
Top lead SNP
Lead SNPs
Independent significant SNPs
Mapped genes
Non-mapped protein coding genes
Non-mapped non-coding genes
Chromosome 15

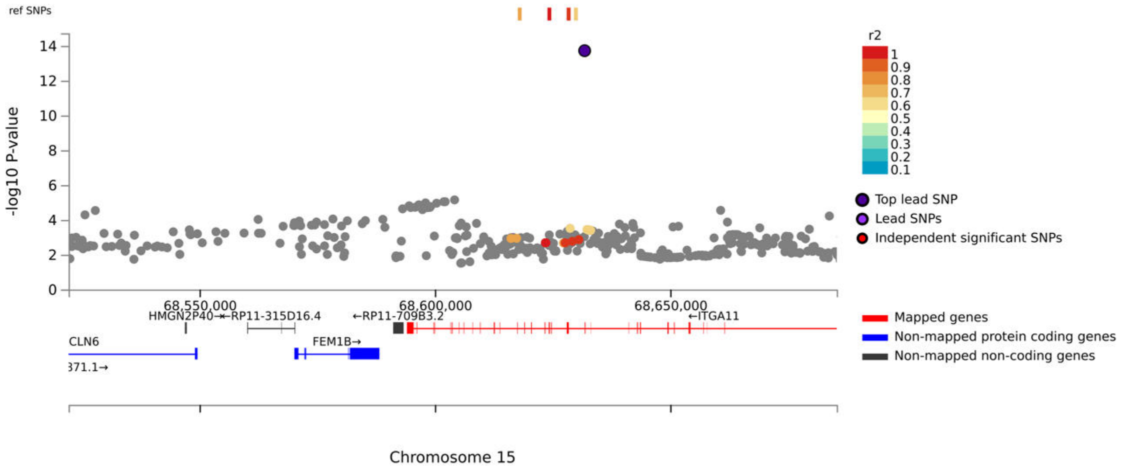

ref SNPs
-log10 P-value
68,550,000
68,600,000
68,650,000
HMGN2P40→
←RP11-315D16.4
←RP11-709B3.2
←ITGA11
CLN6
FEM1B→
r2
Top lead SNP
Lead SNPs
Independent significant SNPs
Mapped genes
Non-mapped protein coding genes
Non-mapped non-coding genes
Chromosome 15

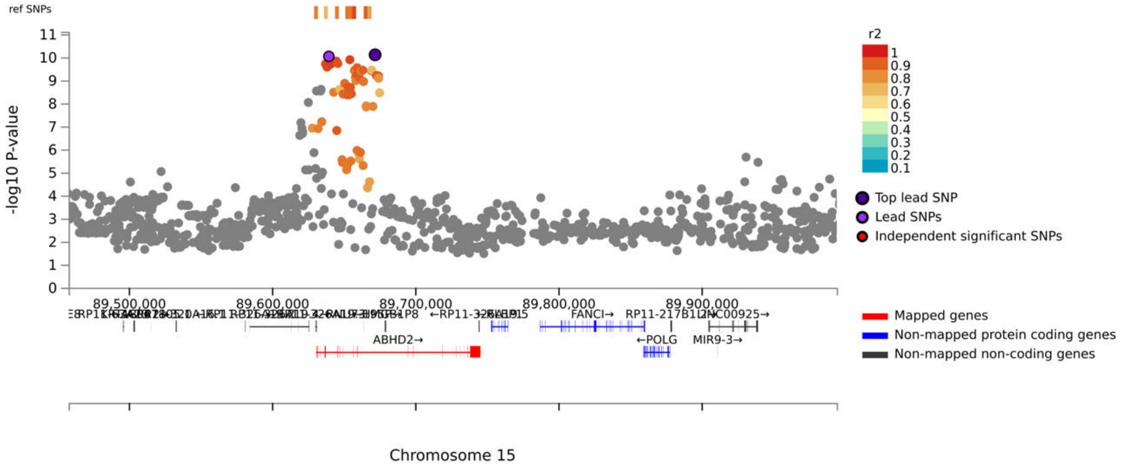

ref SNPs
-log10 P-value
r2
Top lead SNP
Lead SNPs
Independent significant SNPs
ABHD2→
FANCI→
←POLG
MIR9-3→
Mapped genes
Non-mapped protein coding genes
Non-mapped non-coding genes
Chromosome 15

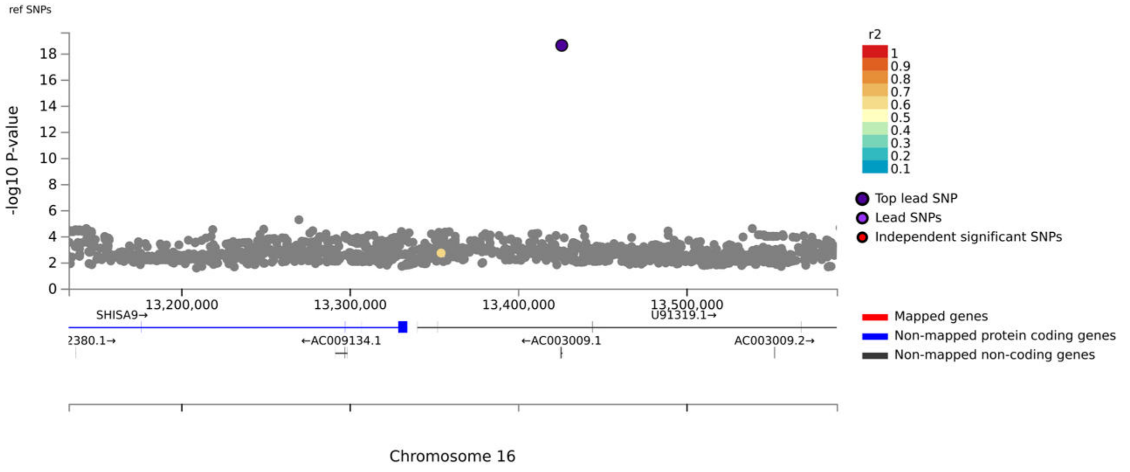

ref SNPs
-log10 P-value
r2
Top lead SNP
Lead SNPs
Independent significant SNPs
SHISA9→
U91319.1→
←AC009134.1
←AC003009.1
AC003009.2→
Mapped genes
Non-mapped protein coding genes
Non-mapped non-coding genes
Chromosome 16

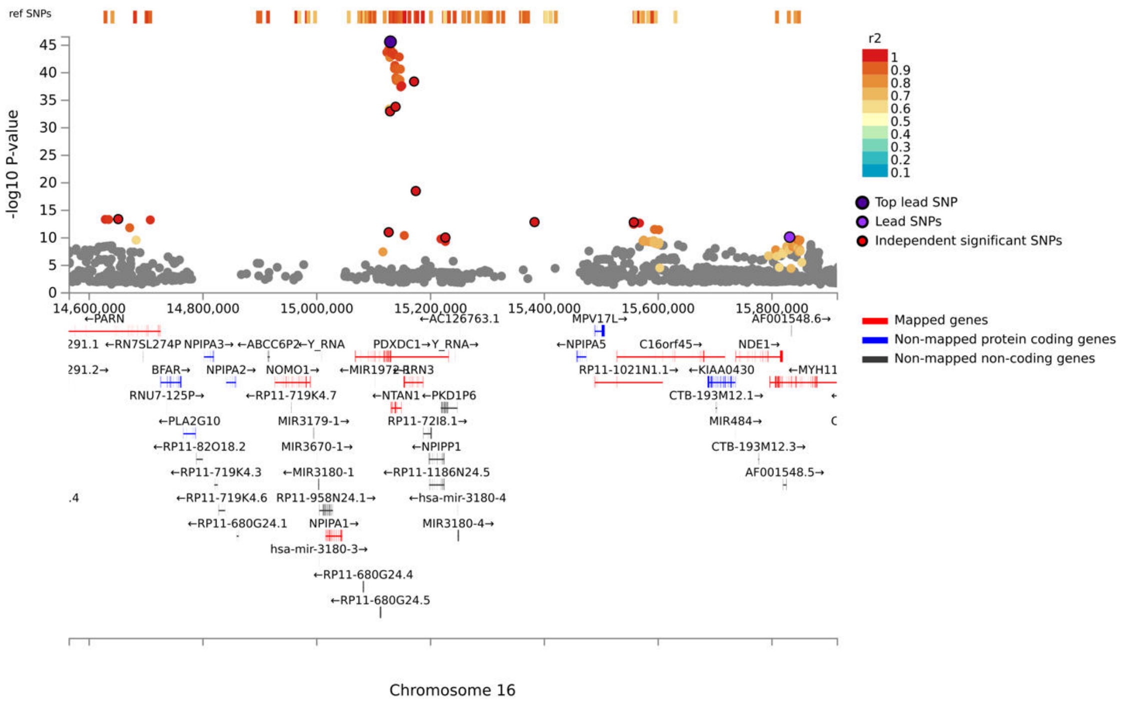
ref SNPs
-log10 P-value
45
40
35
30
25
20
15
10
5
0
14,600,000
14,800,000
15,000,000
15,200,000
15,400,000
15,600,000
15,800,000
r2
1
0.9
0.8
0.7
0.6
0.5
0.4
0.3
0.2
0.1
Top lead SNP
Lead SNPs
Independent significant SNPs
Mapped genes
Non-mapped protein coding genes
Non-mapped non-coding genes
←PARN
←RN7SL274P
NPIPA3→
←ABCC6P2
←Y_RNA
PDXDC1→
←Y_RNA→
←AC126763.1
MPV17L→
←NPIPA5
C16orf45→
NDE1→
AF001548.6→
BFAR→
NPIPA2→
NOMO1→
←MIR1972-2
←RRN3
RP11-1021N1.1→
←KIAA0430
←MYH11
RNU7-125P→
←RP11-719K4.7
←NTAN1
←PKD1P6
CTB-193M12.1→
←PLA2G10
MIR3179-1→
RP11-72I8.1→
MIR484→
←RP11-82O18.2
MIR3670-1→
←NPIPP1
CTB-193M12.3→
←RP11-719K4.3
←MIR3180-1
←RP11-1186N24.5
AF001548.5→
←RP11-719K4.6
RP11-958N24.1→
←hsa-mir-3180-4
←RP11-680G24.1
NPIPA1→
MIR3180-4→
hsa-mir-3180-3→
←RP11-680G24.4
←RP11-680G24.5
Chromosome 16

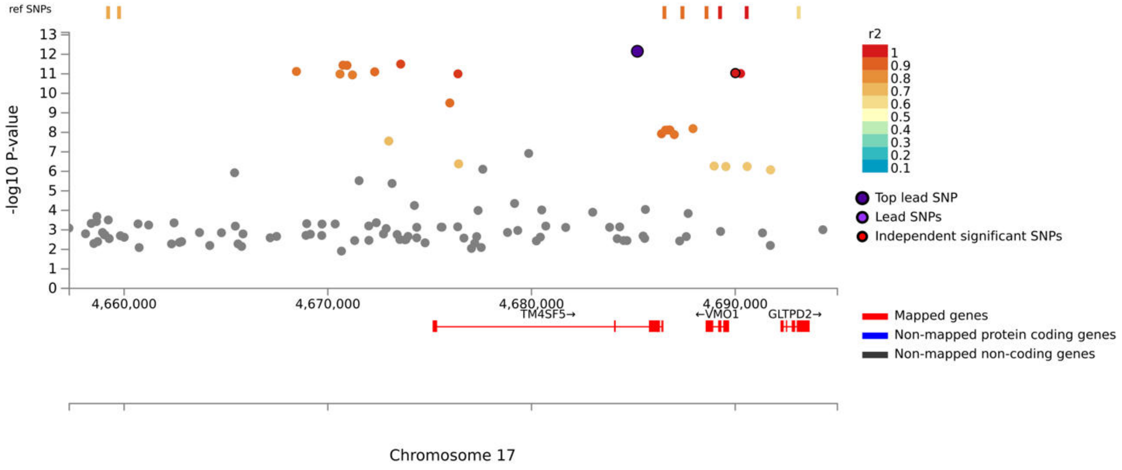
ref SNPs
-log10 P-value
13
12
11
10
9
8
7
6
5
4
3
2
1
0
4,660,000
4,670,000
4,680,000
4,690,000
r2
1
0.9
0.8
0.7
0.6
0.5
0.4
0.3
0.2
0.1
Top lead SNP
Lead SNPs
Independent significant SNPs
Mapped genes
Non-mapped protein coding genes
Non-mapped non-coding genes
TM4SF5→
←VMO1
GLTPD2→
Chromosome 17

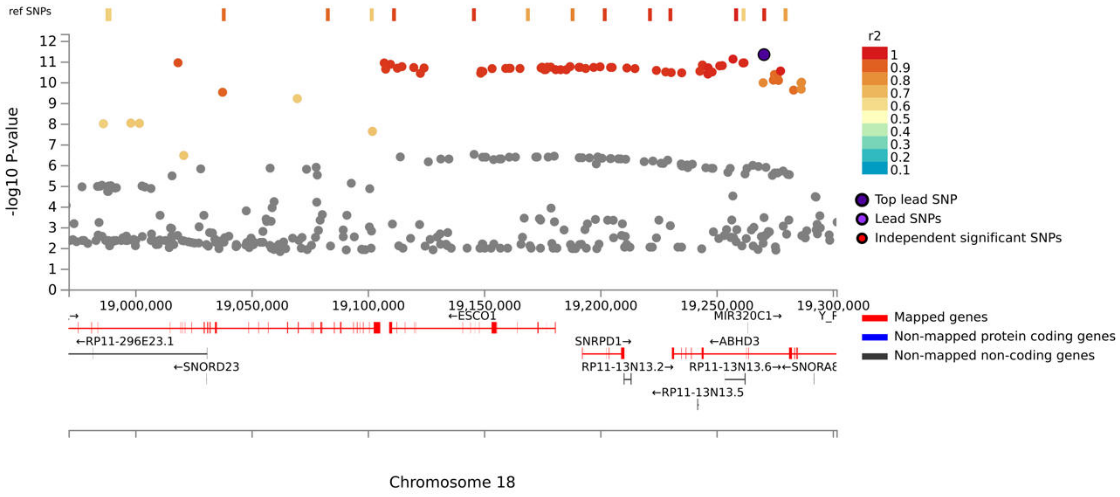

ref SNPs
-log10 P-value
r2
Top lead SNP
Lead SNPs
Independent significant SNPs
19,000,000
19,050,000
19,100,000
19,150,000
19,200,000
19,250,000
19,300,000
←ESCO1
MIR320C1→
←RP11-296E23.1
SNRPD1→
←ABHD3
←SNORD23
RP11-13N13.2→
RP11-13N13.6→
←RP11-13N13.5
Mapped genes
Non-mapped protein coding genes
Non-mapped non-coding genes
Chromosome 18

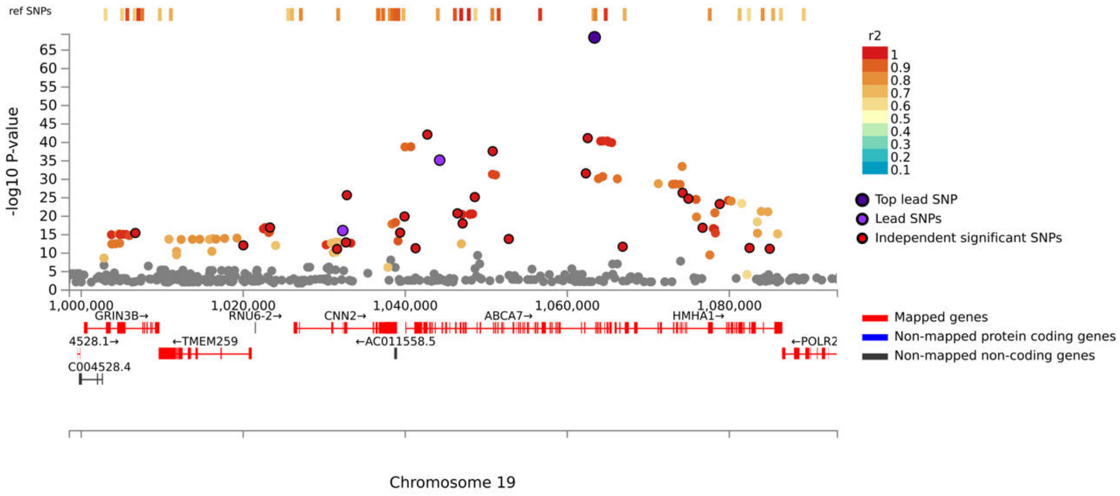

ref SNPs
-log10 P-value
r2
Top lead SNP
Lead SNPs
Independent significant SNPs
1,000,000
1,020,000
1,040,000
1,060,000
1,080,000
GRIN3B→
RNU6-2→
CNN2→
ABCA7→
HMHA1→
←TMEM259
←AC011558.5
Mapped genes
Non-mapped protein coding genes
Non-mapped non-coding genes
Chromosome 19

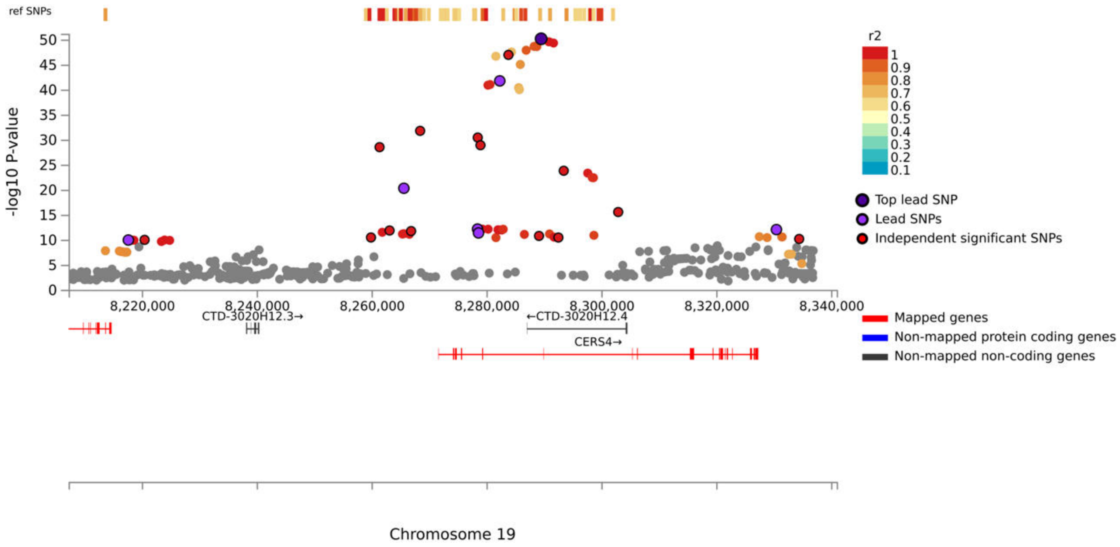

ref SNPs
-log10 P-value
r2
Top lead SNP
Lead SNPs
Independent significant SNPs
8,220,000
8,240,000
8,260,000
8,280,000
8,300,000
8,320,000
8,340,000
CTD-3020H12.3→
←CTD-3020H12.4
CERS4→
Mapped genes
Non-mapped protein coding genes
Non-mapped non-coding genes
Chromosome 19

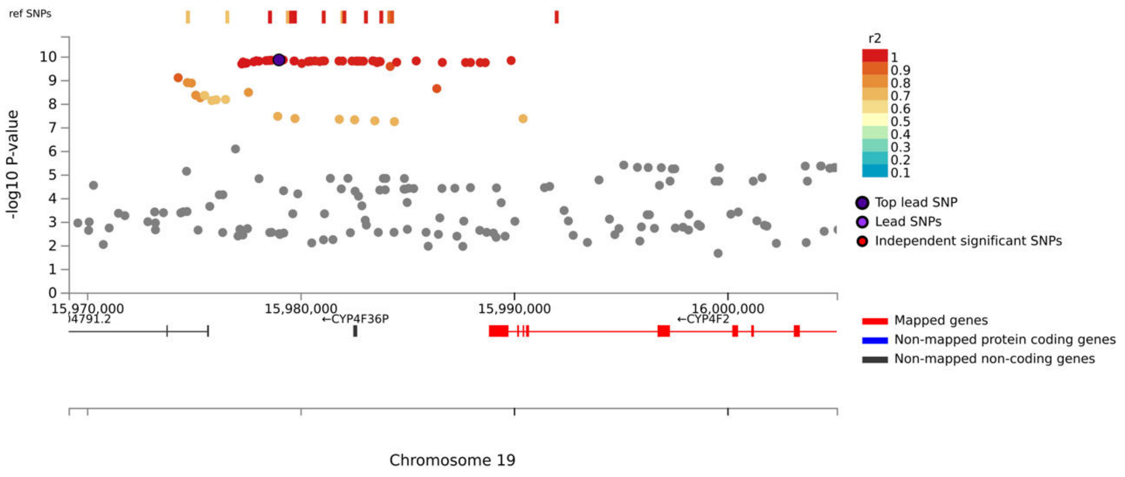

ref SNPs
-log10 P-value
r2
Top lead SNP
Lead SNPs
Independent significant SNPs
15,970,000
15,980,000
15,990,000
16,000,000
←CYP4F36P
←CYP4F2
Mapped genes
Non-mapped protein coding genes
Non-mapped non-coding genes
Chromosome 19

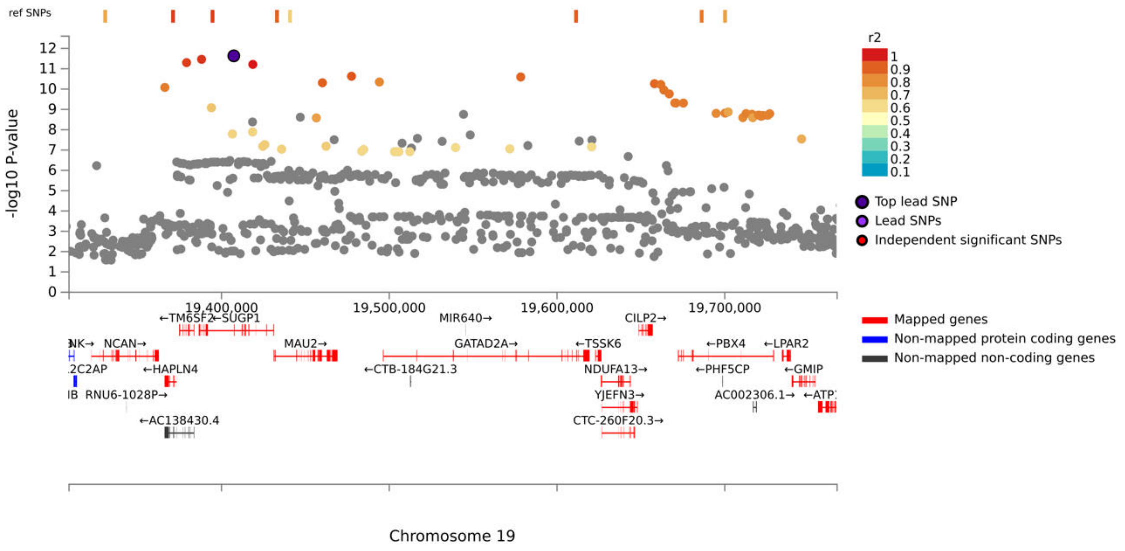

ref SNPs
-log10 P-value
r2
Top lead SNP
Lead SNPs
Independent significant SNPs
19,400,000
19,500,000
19,600,000
19,700,000
←TM6SF2
←SUGP1
MIR640→
CILP2→
NCAN→
MAU2→
GATAD2A→
←TSSK6
←PBX4
←LPAR2
←HAPLN4
←CTB-184G21.3
NDUFA13→
←PHF5CP
←GMIP
RNU6-1028P→
YJEFN3→
AC002306.1→
←AC138430.4
CTC-260F20.3→
Mapped genes
Non-mapped protein coding genes
Non-mapped non-coding genes
Chromosome 19

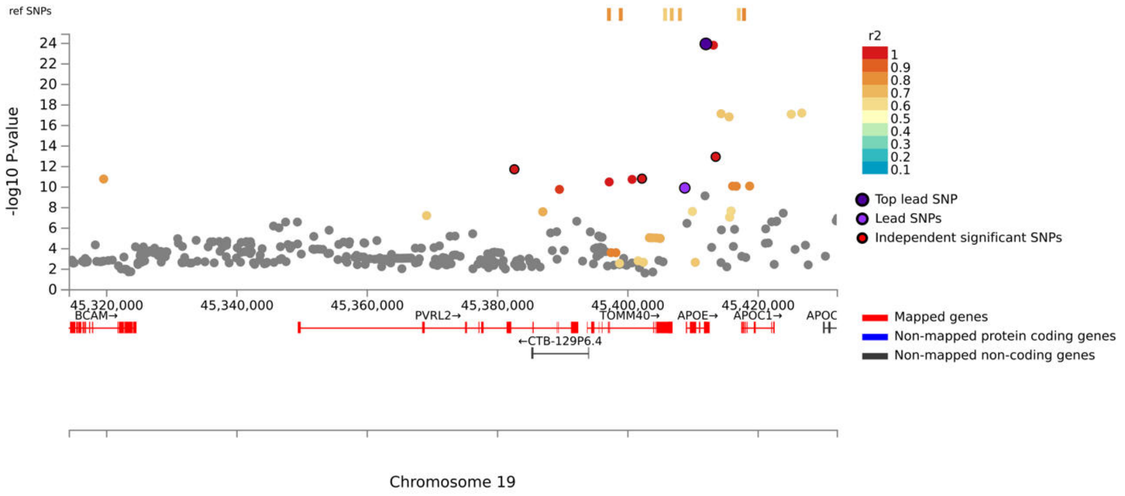

ref SNPs
-log10 P-value
r2
Top lead SNP
Lead SNPs
Independent significant SNPs
45,320,000
45,340,000
45,360,000
45,380,000
45,400,000
45,420,000
BCAM→
PVRL2→
TOMM40→
APOE→
APOC1→
←CTB-129P6.4
Mapped genes
Non-mapped protein coding genes
Non-mapped non-coding genes
Chromosome 19

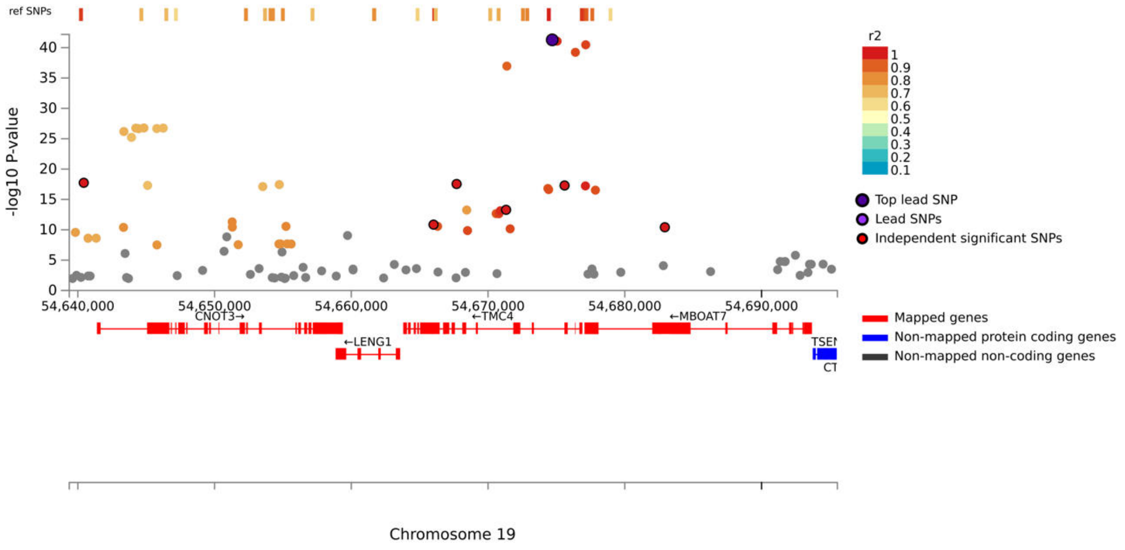
ref SNPs
-log10 P-value
0
5
10
15
20
25
30
35
40
54,640,000
54,650,000
54,660,000
54,670,000
54,680,000
54,690,000
CNOT3→
←TMC4
←MBOAT7
←LENG1
r2
1
0.9
0.8
0.7
0.6
0.5
0.4
0.3
0.2
0.1
Top lead SNP
Lead SNPs
Independent significant SNPs
Mapped genes
Non-mapped protein coding genes
Non-mapped non-coding genes
Chromosome 19

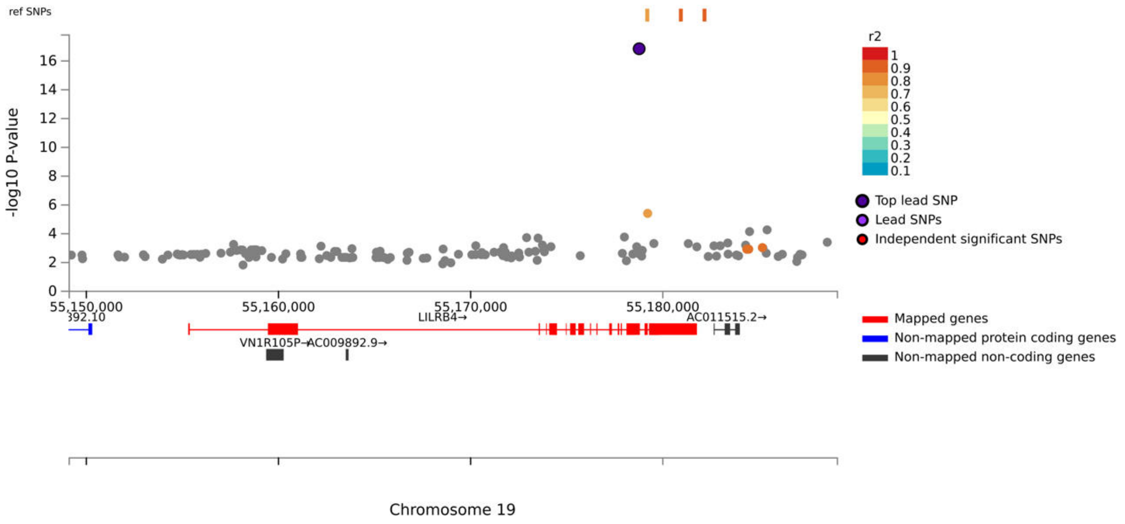
ref SNPs
-log10 P-value
0
2
4
6
8
10
12
14
16
55,150,000
55,160,000
55,170,000
55,180,000
LILRB4→
AC011515.2→
VN1R105P→
AC009892.9→
r2
1
0.9
0.8
0.7
0.6
0.5
0.4
0.3
0.2
0.1
Top lead SNP
Lead SNPs
Independent significant SNPs
Mapped genes
Non-mapped protein coding genes
Non-mapped non-coding genes
Chromosome 19

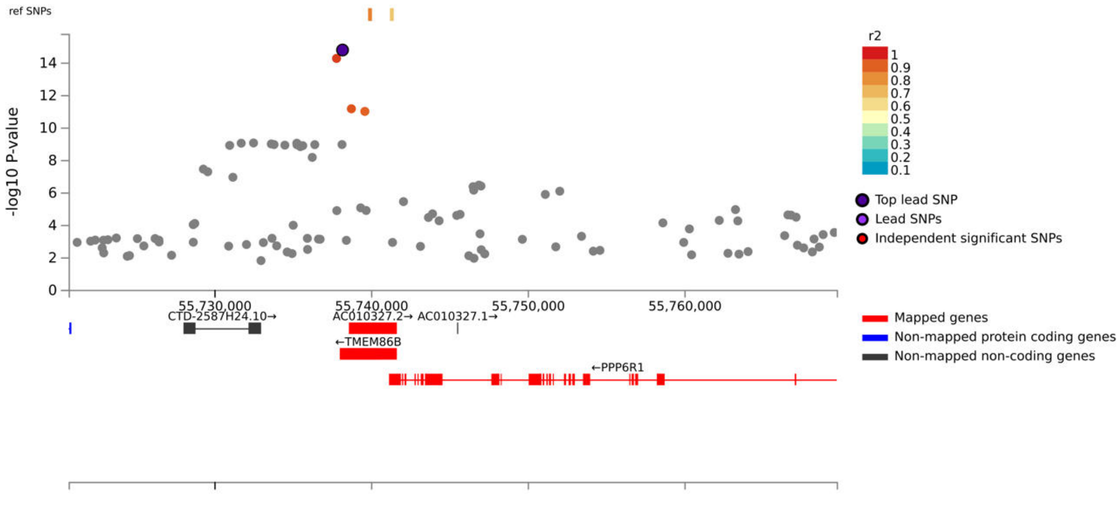

Chromosome 19

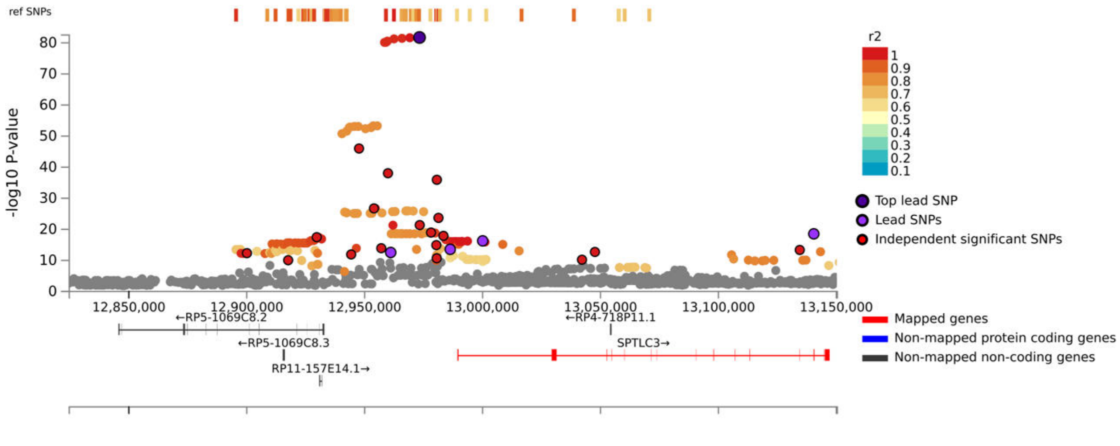

Chromosome 20

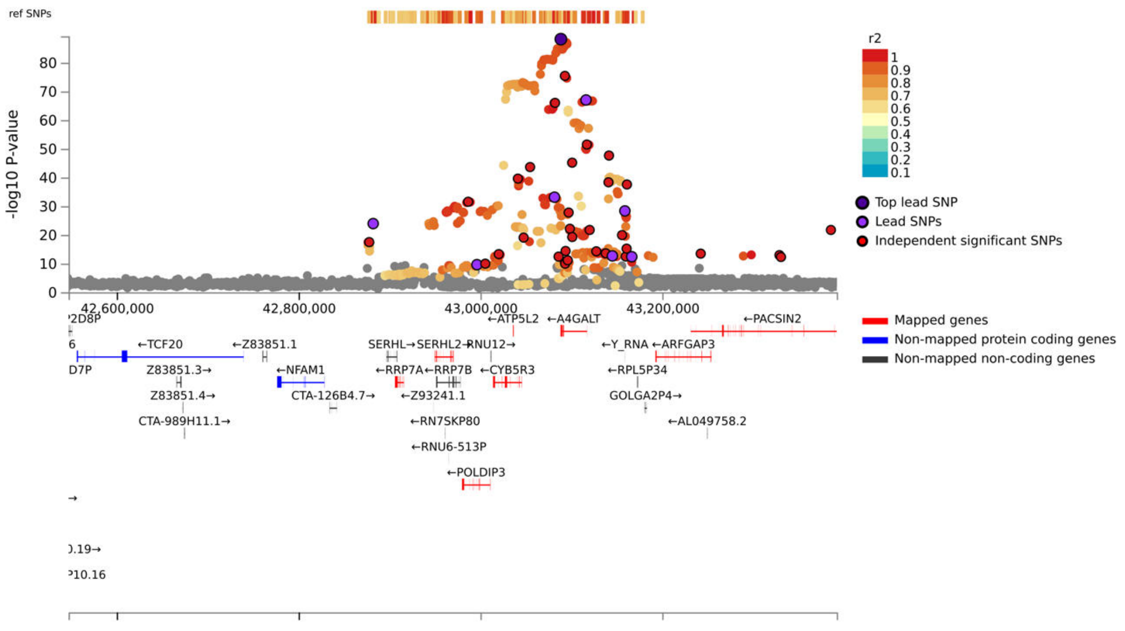

Chromosome 22

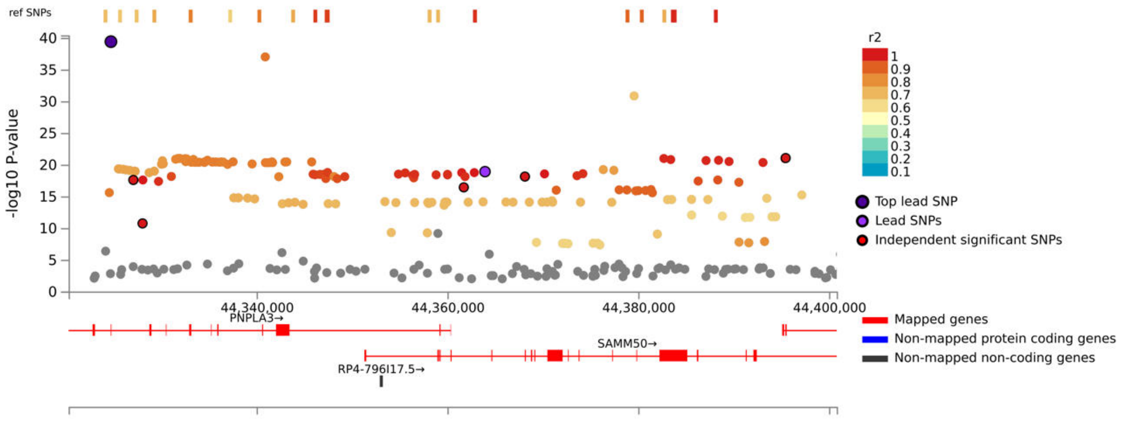

Chromosome 22

Supplementary Figure 3: Genetic correlation of fatty acid composite measures with traditional lipid measurements, The genetic correlation of fatty acid composite measures by the number of carbons (**a**), or double bonds (**b**), with HDL-C, LDL-C and total triglycerides after adjustment for age, sex and the first 10 genetic principal components, Statistical significance is indicated by colours, Fatty acid composite measures based on genetic correlation within each class, which overlapped with at least two clinical lipid measurements, are annotated

(a)

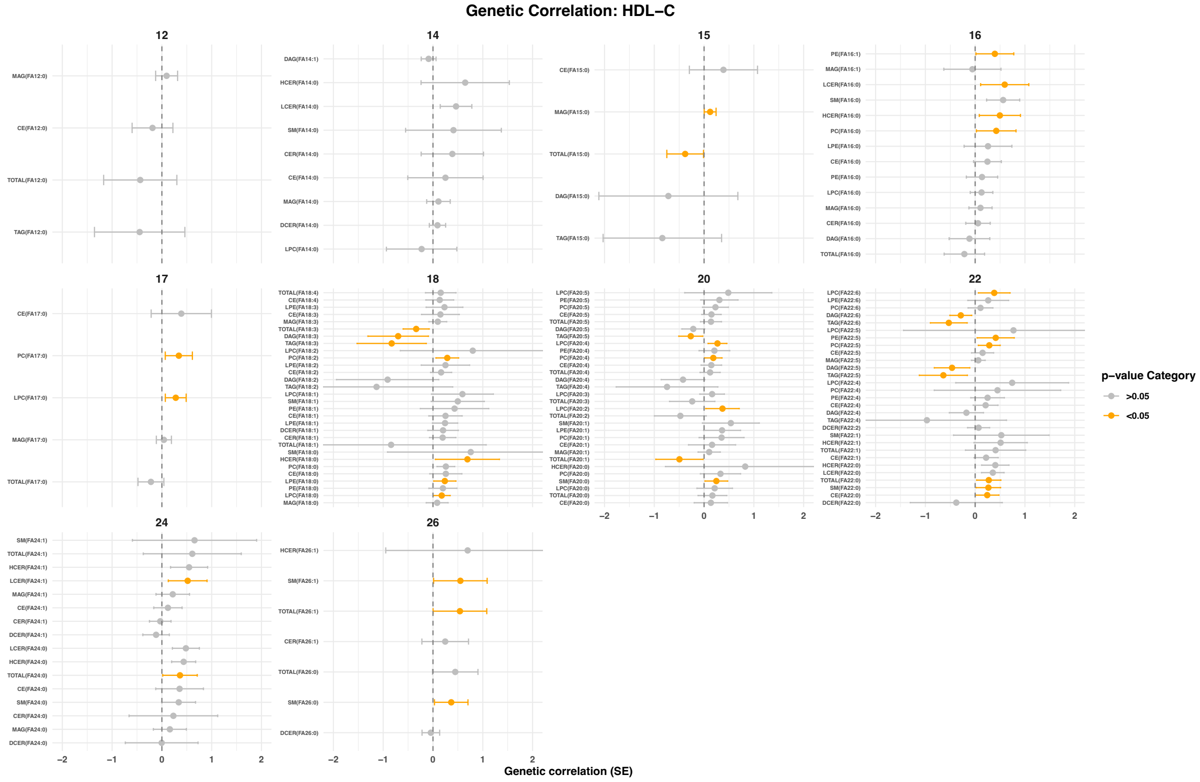

(b)

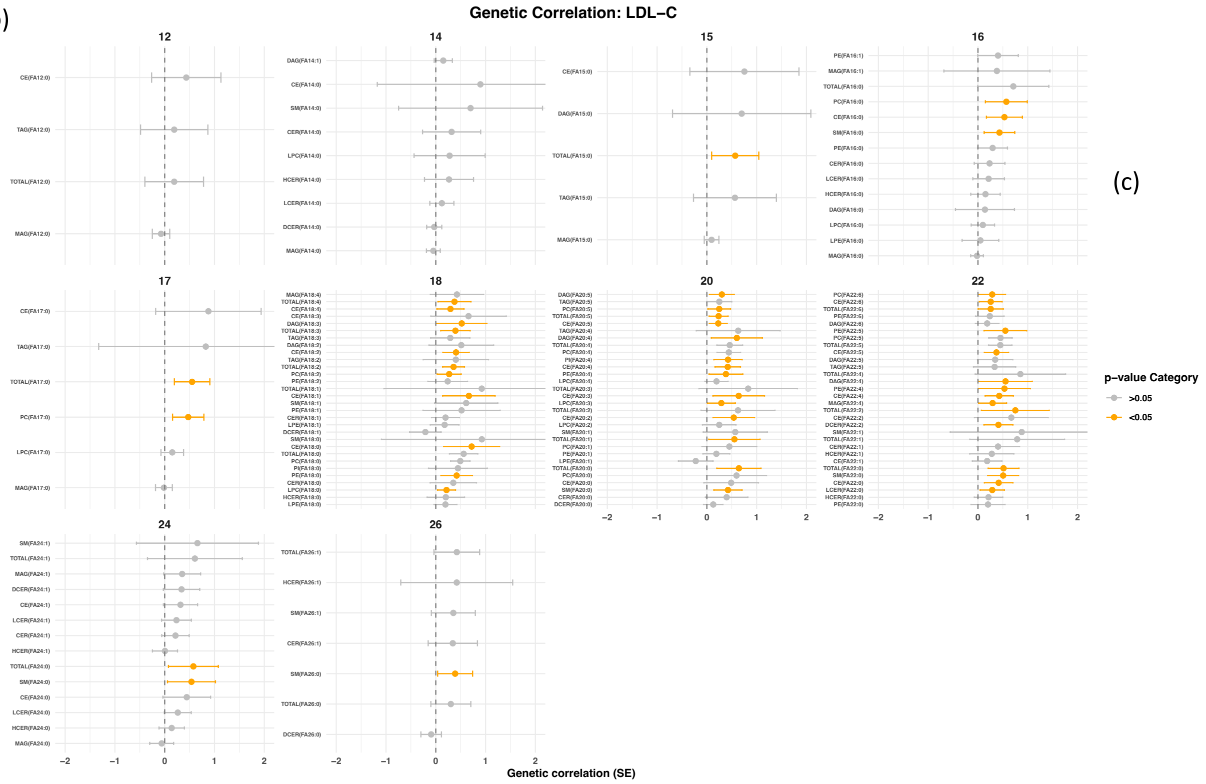

Genetic Correlation: LDL−C
12
14
15
16
17
18
20
22
24
26
p−value Category
>0.05
<0.05
Genetic correlation (SE)

(c)

(c)

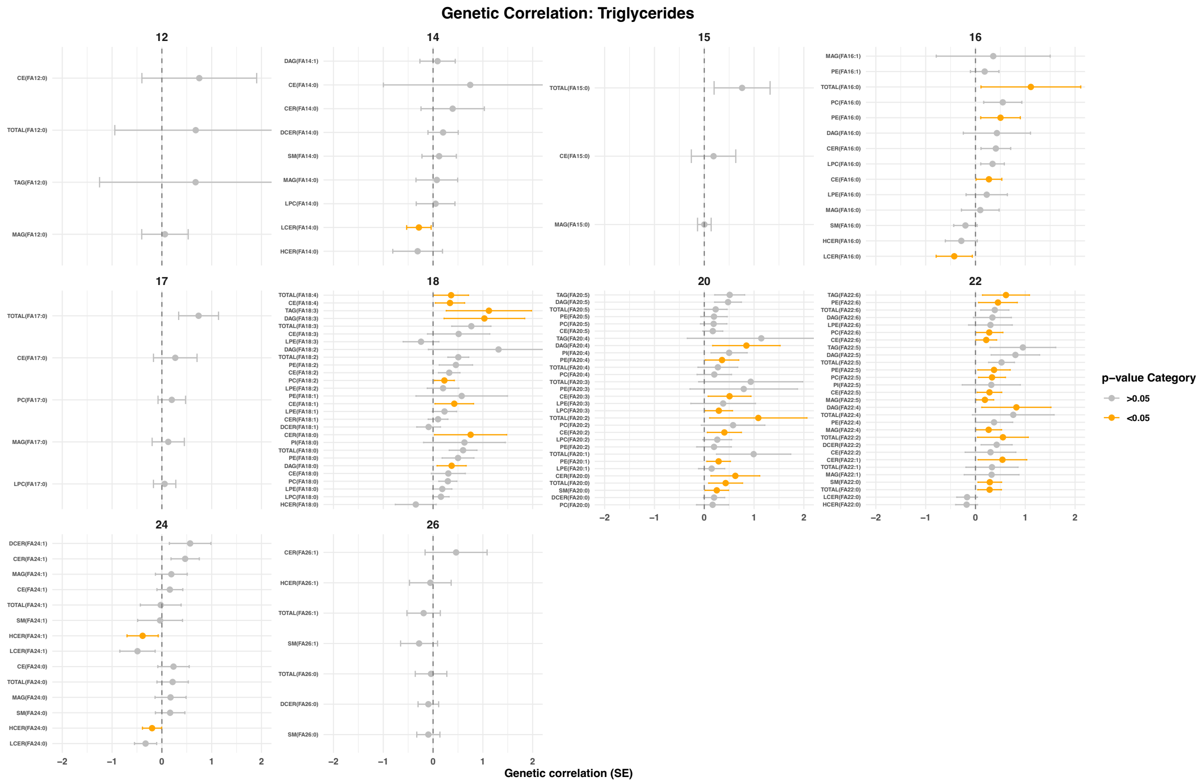

Genetic Correlation: Triglycerides
12
14
15
16
17
18
20
22
24
26
p−value Category
>0.05
<0.05
Genetic correlation (SE)

Supplementary Figure 4: Hudson plots for (a) PEP (18:0/20:2)) and (b) TAG54:4(FA18:0), stratified by sex

(a)

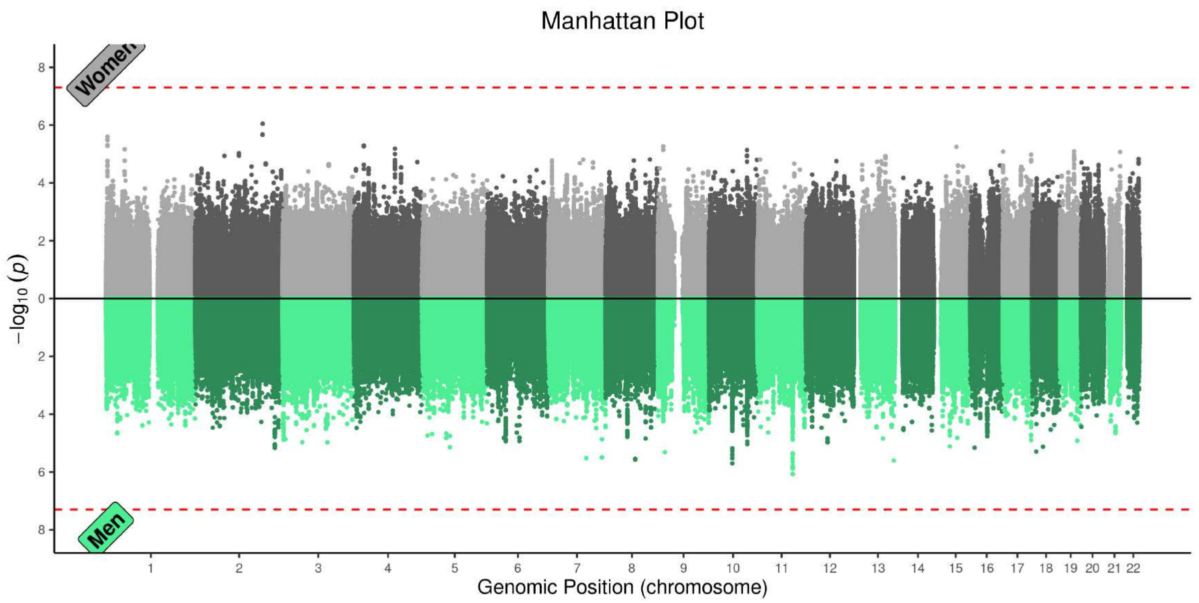

(b)

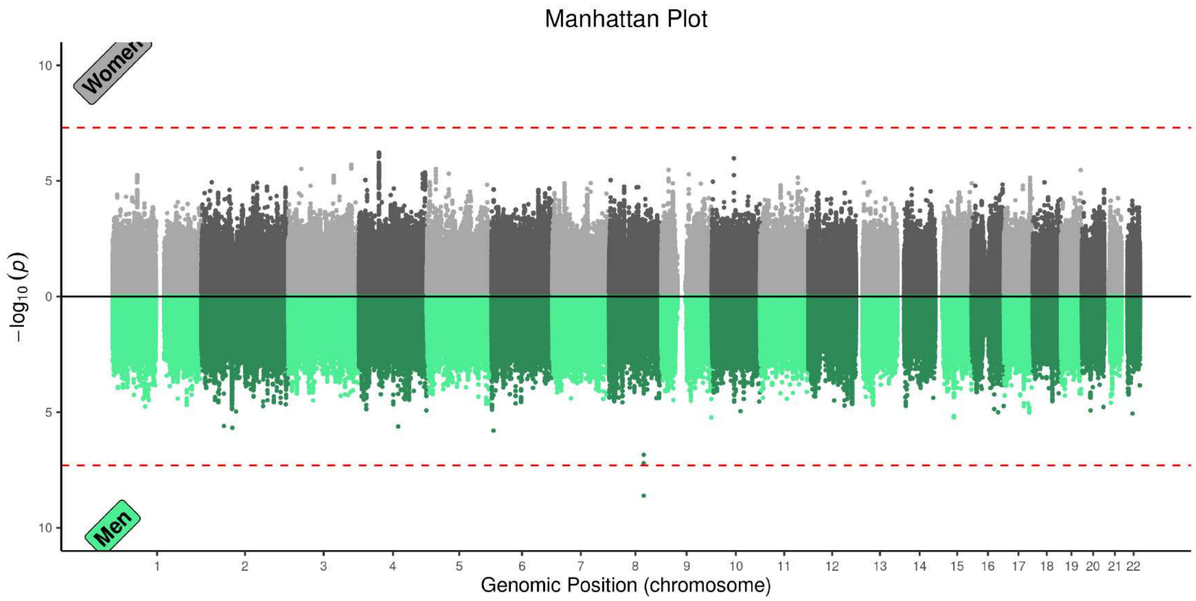

Manhattan Plot
Women
Men
10
5
0
5
10
−log10 (p)
1
2
3
4
5
6
7
8
9
10
11
12
13
14
15
16
17
18
19
20
21
22
Genomic Position (chromosome)

Supplementary Figure 5: Heritability estimates for lipid species and fatty acid composition, (a) Boxplots display the different SNP heritability estimates based on Genome-wide Complex Trait Analysis (GCTA) for model 1 (adjusted for age, sex and the first 10 genetic principal components for lipid species within respective classes, The lipid classes are sorted from left to right based on the median value of the heritability estimates of their lipid species, The degree of heritability for fatty acid composition by the number of carbon atoms and unsaturated bonds for model 1 **(b)**, with lighter colour and larger disk size indicating a higher heritability estimate

(a)

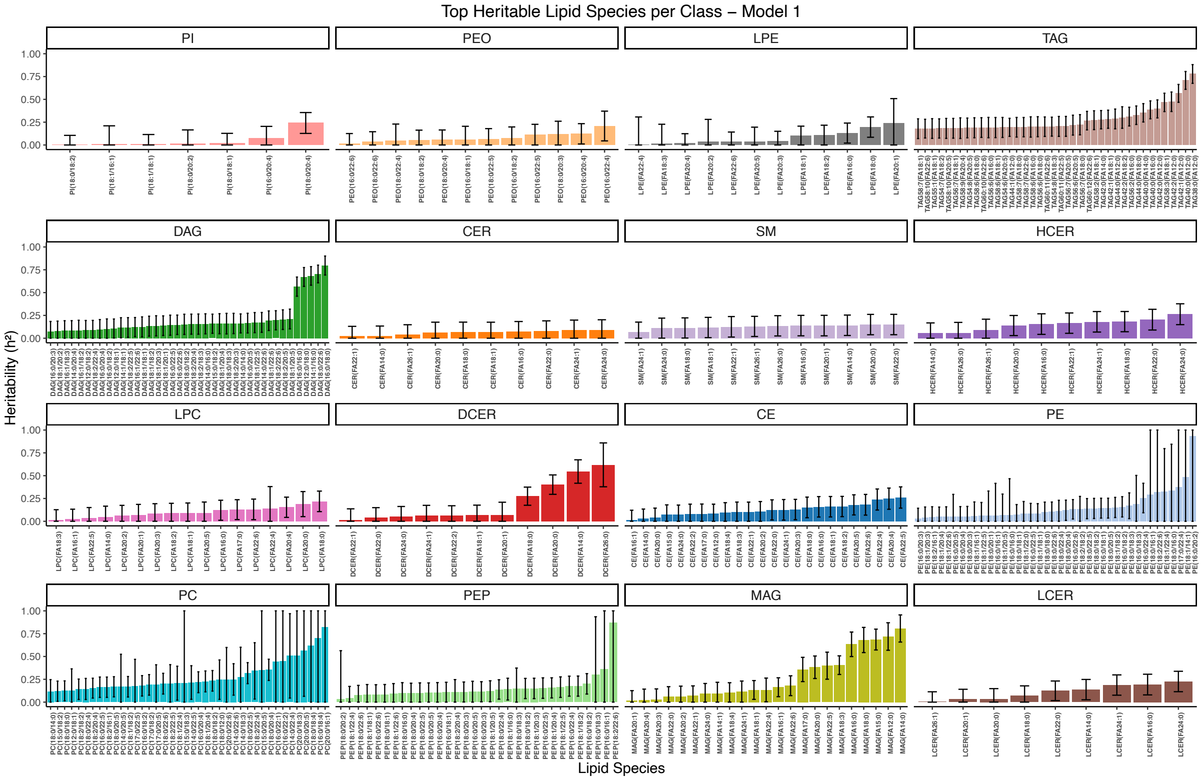

(b)

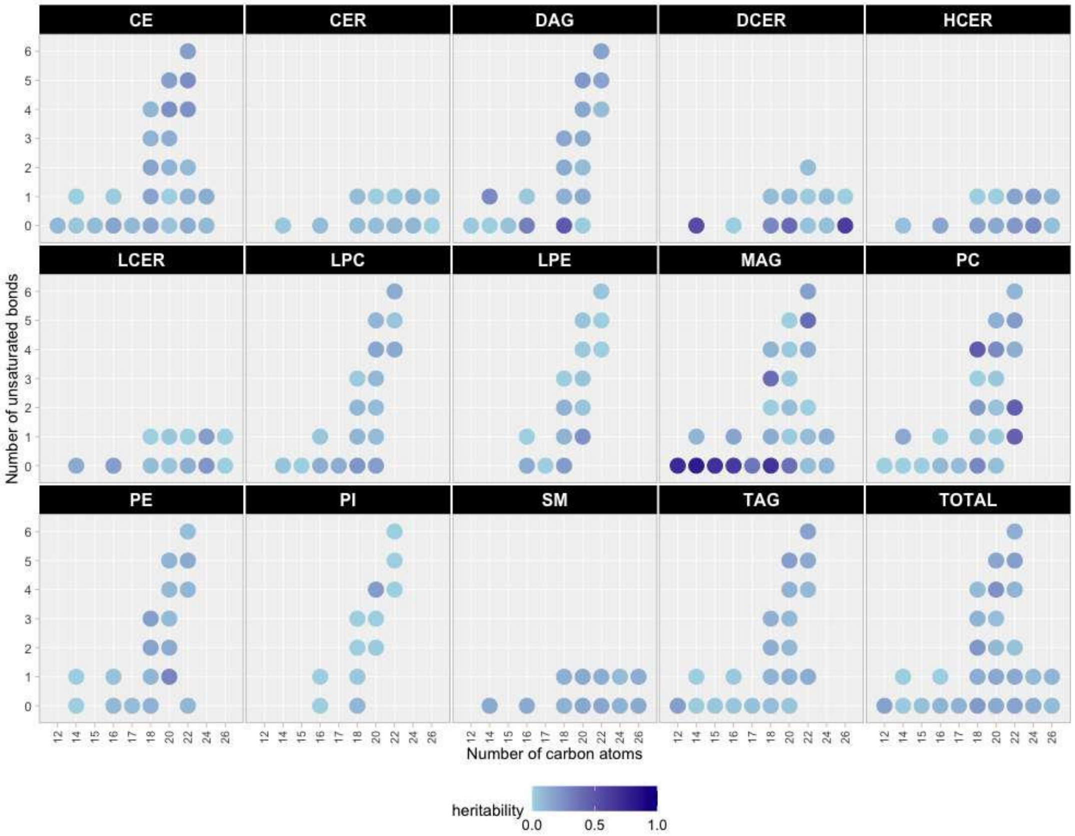
CE
CER
DAG
DCER
HCER
LCER
LPC
LPE
MAG
PC
PE
PI
SM
TAG
TOTAL
Number of unsaturated bonds
Number of carbon atoms
0
1
2
3
4
5
6
12
14
15
16
17
18
20
22
24
26
heritability
0.0
0.5
1.0

Supplementary Figure 6: GWAS replication results, (a) The metabolome-wide significant SNP-lipid pair associations overlapping between the Rhineland Study and the FinnGen cohort for model 1 (adjusted for age, sex, and the first 10 genetic principal components), with replication defined as the association reaching nominal significance in the other cohort, **(b)** The metabolome-wide significant SNP-lipid pair associations overlapping between the Rhineland Study and the EPIC-Potsdam cohort for model 1 (adjusted for age, sex, and the first 10 genetic principal components) and model 2 (additionally adjusted for LDL-C, HDL-C, triglycerides, fasting status and use of lipid-lowering medication), with replication defined as the association reaching nominal significance in the EPIC-Potsdam cohort, **(c)** Scatter plots of SNP effect sizes (β) in the Rhineland study versus replication cohorts (FinnGen, EPIC Potsdam and Busselton Health Study) for model 1, **(d)** Scatter plots of SNP effect sizes (β) in the Rhineland study versus replication cohorts (EPIC Potsdam and Busselton Health Study) for model 2, *Abbreviations: HDL-C = high-density lipoprotein cholesterol, LDL-C = low-density lipoprotein cholesterol*

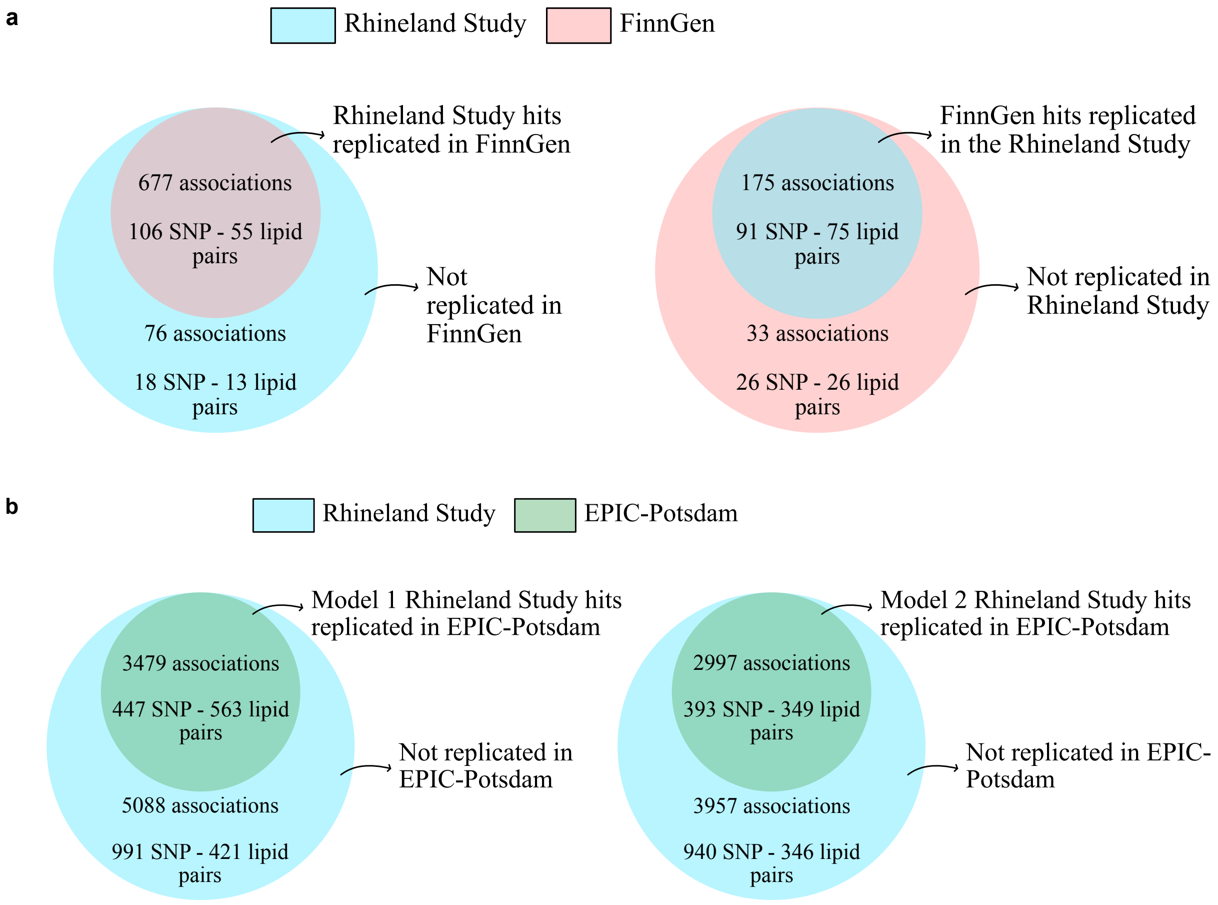

c

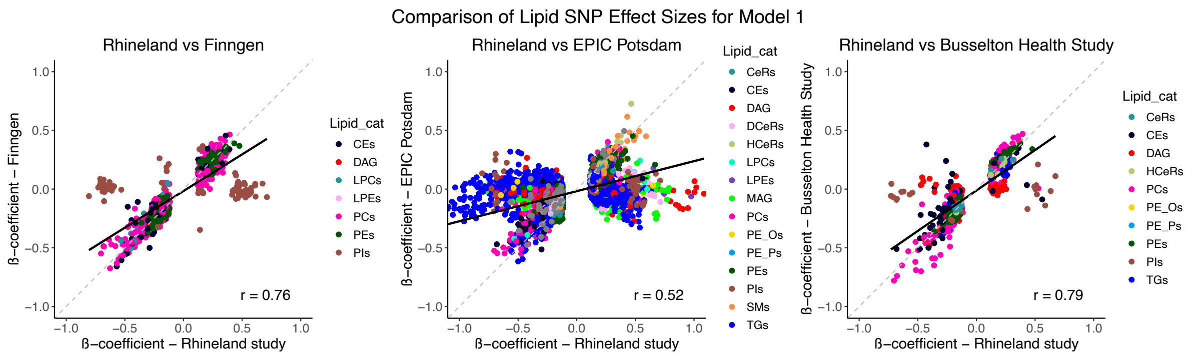
Comparison of Lipid SNP Effect Sizes for Model 1
Rhineland vs Finngen
ß−coefficient − Finngen
ß−coefficient − Rhineland study
r = 0.76
Lipid_cat
CEs
DAG
LPCs
LPEs
PCs
PEs
PIs
Rhineland vs EPIC Potsdam
ß−coefficient − EPIC Potsdam
ß−coefficient − Rhineland study
r = 0.52
Lipid_cat
CeRs
CEs
DAG
DCeRs
HCeRs
LPCs
LPEs
MAG
PCs
PE_Os
PE_Ps
PEs
PIs
SMs
TGs
Rhineland vs Busselton Health Study
ß−coefficient − Busselton Health Study
ß−coefficient − Rhineland study
r = 0.79
Lipid_cat
CeRs
CEs
DAG
HCeRs
PCs
PE_Os
PE_Ps
PEs
PIs
TGs

d

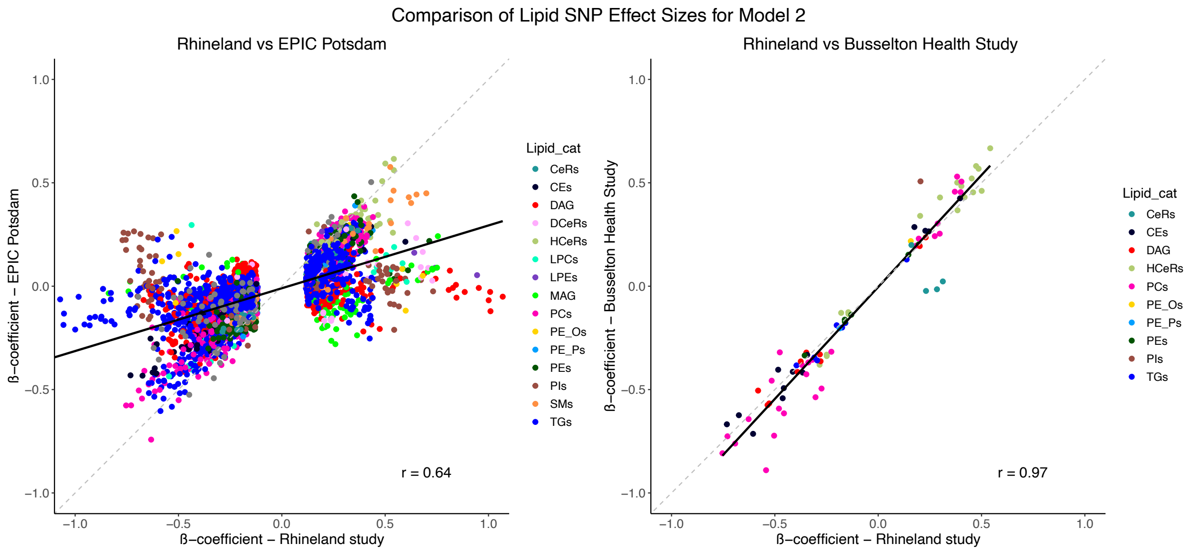
Comparison of Lipid SNP Effect Sizes for Model 2
Rhineland vs EPIC Potsdam
ß−coefficient − EPIC Potsdam
ß−coefficient − Rhineland study
r = 0.64
Lipid_cat
CeRs
CEs
DAG
DCeRs
HCeRs
LPCs
LPEs
MAG
PCs
PE_Os
PE_Ps
PEs
PIs
SMs
TGs
Rhineland vs Busselton Health Study
ß−coefficient − Busselton Health Study
ß−coefficient − Rhineland study
r = 0.97
Lipid_cat
CeRs
CEs
DAG
HCeRs
PCs
PE_Os
PE_Ps
PEs
PIs
TGs
