# Supplementary material for: Population-based genome-wide association study of plasma complex lipid species
Source: Nat Commun. 2026 May 2;17:3984. doi: 10.1038/s41467-026-72542-1 (PMC13135507; doi:10.1038/s41467-026-72542-1)
Supplement: Supplementary file 2 — Description of Additional Supplementary Files [file 41467_2026_72542_MOESM2_ESM.pdf]

Description of Additional Supplementary Files

Supplementary Data 1: Model 1: Genomic loci associated with lipid species

Supplementary Data 2: Overview of the number of metabolome-wide significant independent SNPs and genomic loci associated with faHy acid (FA) composite measures (n= 267) by the number of carbons

Supplementary Data 3: Overview of the number of metabolome-wide significant independent SNPs and genomic loci with faHy acid (FA) composite measures (n= 267) by the number of double bonds

Supplementary Data 4: Model 1: Genomic loci associated with faHy acid composites

Supplementary Data 5: Genetic correlation of lipid species and fatty acid composite measures

Supplementary Data 6: Model 2: Genomic loci associated with lipid species

Supplementary Data 7: Model 2: Genomic loci associated with faHy acid composites

Supplementary Data 8: Comparison of model 1 and model 2 for top-significant SNPs in lipid species and fatty acid composite measures

Supplementary Data 9: SNP heritability of the lipid species and fatty acid composite measures

Supplementary Data 10: Model 1 & Model 2 : ProGEM framework analysis for lipid species

Supplementary Data 11: Model 1 & Model 2: ProGEM framework analysis for fatty acid composite measures

Supplementary Data 12: Look-up analysis of FinnGen GWAS results in the Rhineland Study

Supplementary Data 13: Overlap of lipid species in Rhineland study with validation cohorts (FinnGen and EPIC-Potsdam)

Supplementary Data 14: Model 1: Genomic loci associated with lipid species in meta-GWAS

Supplementary Data 15: Model 2: Genomic loci associated with lipid species in meta-GWAS

Supplementary Data 16: Model 1 & Model 2: ProGEM framework analysis for lipid species in meta-GWAS

Supplementary Data 17: Overview of disease based PheWas lookups

Supplementary Data 18: Colocalisation analysis with diseases

Supplementary Data 19: Twosample MR with diseases

Supplementary Data 20: One-sample and Two sample MR of primary candidate genes from GTEx in whole blood associations with lipid species

Supplementary Data 21: Harmonization of SNP instruments between gene expression (exposure) and lipid species (outcome) prior to Forward two-sample Mendelian randomization analysis

Supplementary Data 22: Overview of primary candidate genes associations with lipid species and lead SNPs and mediation analysis

Supplementary Data 23: Overlap of Mediation analysis and one-sample MR

Supplementary Data 24: Missingness per lipid species

Supplementary Data 25: Harmonization of SNP instruments prior to Forward/Reverse two-sample Mendelian randomization analyses
